# CsmR controls both, motility and cell shape, in *Haloferax volcanii*

**DOI:** 10.1371/journal.pgen.1012198

**Published:** 2026-06-12

**Authors:** Phillip Nußbaum, Felix Grünberger, Felix Neuschütz, Kevin Chou, Shamphavi Sivabalasarma, Alexander Eulitz, Anna-Lena Sailer, Katharina Vogl, Marten Exterkate, Wei He, Anita Marchfelder, Dina Grohmann, Sonja-Verena Albers

**Affiliations:** 1 Molecular Biology of Archaea, Faculty of Biology, University of Freiburg, Freiburg, Germany; 2 Institute of Biochemistry Genetics and Microbiology, Institute of Microbiology and Archaea Centre, Single-Molecule Biochemistry Lab & Biochemistry Centre Regensburg, University of Regensburg, Regensburg, Germany; 3 Spemann Graduate School of Biology and Medicine, University of Freiburg, Freiburg, Germany; 4 Molecular Biology and Biotechnology of Prokaryotes, University of Ulm, Ulm, Germany; 5 Department of Membrane Biogenesis and Lipidomics, Institute of Biochemistry, Heinrich Heine University Düsseldorf, Düsseldorf, Germany; 6 CIBSS- Centre for Integrative Biological Signaling Studies, Faculty of Biology, University of Freiburg, Freiburg, Germany; The University of Texas Southwestern Medical Center, UNITED STATES OF AMERICA

## Abstract

Archaea rely on motility and morphological plasticity to navigate their environments, yet the transcriptional regulation of these processes remains poorly understood. In *Haloferax volcanii*, archaellum-dependent motility is transcriptionally regulated, but an EarA-like central regulator for transcription of archaellum genes that is found in other Euryarchaeota like *Methanococcus maripaludis* or *Pyrococcus furiosus*, is absent. Here, we identify CsmR as a transcriptional regulator that controls archaellum biogenesis and cell-shape transitions in *H. volcanii*. Deletion of *csmR* abolished detectable motility, whereas overexpression increased motility and promoted a sustained rod-like morphology. Comparative transcriptomics defined a CsmR-associated regulon that includes archaellum and chemotaxis genes as well as cell-shape determinants (e.g., Sph3 and RdfA), and ChIP-seq identified promoter-proximal binding sites consistent with direct transcriptional control. Furthermore, *csmR* and *cirA*, a KaiC-like regulator*,* share extensive transcriptional overlap, with CirA potentially fine-tuning CsmR-mediated regulation through post-translational modification. These findings establish CsmR as a key regulator of archaellum gene expression and cell shape regulation in *Haloferax volcanii*, suggesting that haloarchaea coordinate these fundamental processes through an unidentified transcriptional network. Moreover, Northern blotting and cell shape observation suggest that transcription factor RosR is involved in the regulation of an regulatory RNA that shares extensive overlap with the *cirA* gene, possibly fine-tuning the effect of CirA on the regulation of the archaellum cluster and the rod shape determinants *sph3* and *rdfA*. Understanding this interplay provides new insights into archaeal adaptability and may reveal broader regulatory principles in prokaryotic cell biology.

## Author summary

In this study, we explore how archaea regulate their movement and shape. Many archaea use tiny, rotating structures called archaella to swim, and in some species, archaella gene expression are controlled by a well-known transcriptional regulator called EarA. However, in *Haloferax volcanii*, this regulator is absent, suggesting that a different mechanism is at play. We identified a new transcriptional regulator, called CsmR, which plays a key role in both controlling archaella production and coordinating changes in cell shape. When we removed CsmR, the cells could no longer move and also failed to switch between different cell shapes, while increasing its levels made the cells overly motile and locked in a rod-like morphology. By analyzing gene-expression changes and regulatory patterns, we found that CsmR likely interacts with another protein, CirA, to fine-tune movement and shape changes in response to environmental conditions. These findings reveal a previously unknown link between motility and morphology in archaea, offering new insights into how microbes adapt to their surroundings.

## 1. Introduction

The ability of microorganisms for cellular locomotion is a key feature to evade unfavorable environmental conditions such as nutrient limitation, changes in pH or temperature. The most widespread form of cellular movement is swimming motility, in which cells use a rotating appendage to generate force and propel themselves through liquid media. Depending on the phylogenetic group, different cellular machineries have evolved, including cilia in Eukaryotes, flagella in Bacteria, and archaella in Archaea [1]. The archaellum shares homology with archaeal and bacterial type-IV pili with respect to the proteins involved and in their mode of assembly [2,3]. Across archaeal phyla, the archaellum is composed of different sets of proteins that share a conserved core machinery [4]. Those core components include the polytopic membrane protein ArlJ [5], the assembly ATPase ArlI [6], the ATP binding protein ArlH [7,8], stator proteins ArlF/ArlG [9] and the filament forming archaellins [10]. Typically, all archaellum-related genes are clustered together in a single operon [4,10,11]. In contrast to the bacterial flagellum, which is powered by the proton motive force [12], archaellum rotation is driven by ATP hydrolysis via ArlI [6,13]. Since ATP is essential for many cellular processes, this energy-intensive mode of locomotion must be tightly controlled. In Thermoproteota diverse transcriptional factors that control the expression of archaellum components have been described. In the thermophilic archaeon *Sulfolobus acidocaldarius,* several positive and negative regulators have been studied, showing that phosphorylation plays a major role in archaellum regulation [4]. The key components of this system collectively referred as archaellum regulatory network (Arn) include the Forkhead associated (FHA) -domain containing protein ArnA and a von Willebrand factor A domain containing protein called ArnB [14]. Both proteins undergo phosphorylation at their C-terminus by the kinase ArnC [14,15]. Additionally, ArnB is phosphorylated by a second kinase, ArnD [15]. Phosphorylation of ArnA and ArnB promotes their interaction and the formation of a higher oligomeric complex [16]. This complex negatively affects archaellum gene expression [14], possibly by binding the *arlB* or *arlX* promotor region, as observed for the ArnA homolog of *Sulfolobus tokodai*, ST0829 [17]. Other components of the archaellum regulatory network include the paralogous transmembrane proteins ArnR and ArnR1, which contain helix-turn-helix motifs in their N-terminal region [18]. The previously mentioned kinase ArnC and a third membrane-associated kinase ArnS have been shown to phosphorylate ArnR and ArnR1 [18,19]. Phosphorylation of both proteins had a positive effect on the activation of the *arlB* promotor, but the second promotor of the archaellum operon, upstream of *arlX,* remained unaffected by ArnR/R1 [18,20]. High upregulation of the archaellum cluster in *S. acidocaldarius* is seen in the late stationary phase of growth or under starvation conditions [21].

In Euryarchaeota a single transcription regulator of the archaellum cluster has been identified so far, named euryarchaeal archaellum regulator A (EarA) [22]. EarA was first characterized in the methanogenic archaeon *Methanococcus maripaludis* where deletion led to non-archaellated cells due to significantly reduced expression of one of the filament-forming subunits of the archaellum, *arlB2*. Electrophoretic mobility shift assays showed that EarA binds, via its winged helix-turn-helix motif, to a region upstream of the archaellum promoter [23]. Interestingly, a spontaneous mutation in the B recognition element (BRE) of the *arlB2* promoter restored archaellation and motility in the *earA* deletion strain. These BRE mutations generated a strong promoter motif, probably bypassing the need for EarA to recruit the transcription factor B (TFB) to the archaellum promoter [24]. These findings suggest that EarA acts as a positive regulator for archaellum expression. Indeed, *earA* overexpression in *Pyrococcus furiosus* resulted in heavily archaellated cells [25].

While EarA has been established as the primary archaellum regulator in many Euryarchaeota, its absence in Haloarchaeota suggests that motility regulation in this group follows a distinct molecular mechanism [22]. Instead of relying on a single dedicated activator, archaellation in halophilic archaea appears to be influenced by environmental factors, cell shape transitions, and potentially alternative regulatory networks. In *Haloarcula marismortui* the salt concentration of the medium influences the composition of the archaellum filament [26], while in *Haloferax volcanii* lower growth temperatures induced strong upregulation of archaellins A1 [27]. Additionally, the ability of *H. volcanii* to swim is closely linked to its cell shape. During early exponential growth *H. volcanii* cells are rod-shaped and motile. As growth progresses, the cells transition from this rod- to a plate-shaped cell morphology in which cells are not motile [28,29]. Plate shaped cells lack archaellum filaments but retain assembled archaellum motors [28]. This transition is not merely a passive consequence of growth phase but is actively mediated by cell-cell communication: the rod-to-plate switch is triggered by a secreted small molecule that accumulates as population density increases, representing the first characterized quorum sensing system in archaea [30]. Cell shape transition of *H. volcanii* is controlled by several proteins. One of those factors is CetZ1, a tubulin homolog that is essential for the formation and maintenance of motile rod-shaped *H. volcanii* cells [31]. A recent study identified three additional proteins involved in the morphological plasticity of *H. volcanii*. Rod-Determination Factor A (RdfA) is essential for the rod-shaped morphology, as its deletion leads to predominantly plate-shaped cells. Conversely, Disk-Determining Factor A (DdfA) and Sph3 are critical for maintaining a plate-shaped morphology, with its deletion resulting in exclusively rod-shaped cells. The third protein, Volactin, an actin homolog, contributes to plate formation through dynamic polymerization and depolymerization [32]. Moreover, a KaiC homolog in *H volcanii* named CirA was shown to impact cell shape and motility. The deletion strain of *cirA* stayed rod-shaped during all growth stages resulting in increased motility compared to the wildtype [33]. KaiC-like proteins are characterized by their ATPase and autophosphorylation activities which in cyanobacteria underpin circadian clock function through cycles of phosphorylation and dephosphorylation [34,35]. In archaea and bacteria KaiC homologs have been implicated in post-translational regulatory mechanisms including modulation of protein-protein interactions and transcription factor activity through phosphorylation-based switches [36,37]. Notably, the archaellum motor component ArlH which is essential for archaellum assembly and function [7,8] is itself a KaiC homolog, demonstrating that KaiC-like proteins are directly integrated into the archaellum regulatory machinery. The biochemical activities of CirA in *H. volcanii* have not yet been characterized but its homology to KaiC family proteins together with the established role of ArlH in archaellum motor function raises the possibility that CirA may influence the activity of regulatory partners through post translational mechanism such as phosphorylation based switches, potentially modulating transcription factor activity in a manner analogous to the phosphorylation dependent archaellum regulators described in *Sulfolobus acidocaldarius* [14,16].

Here, we identify CsmR (Cell shape and motility regulator) as a novel transcriptional regulator of archaellation in *H. volcanii*. Through genetic, transcriptomic, and structural analyses, we demonstrate that CsmR is essential for motility and additionally plays a critical role in coordinating cell shape transitions. Unlike previously described archaellum regulators, CsmR belongs to the Lrp/AsnC family of transcription factors, a widely distributed regulator group that in other systems has been associated with the integration of environmental and metabolic signals, though no such direct sensing role has been demonstrated for CsmR in this study. Our findings reveal that CsmR directly regulates expression of the entire archaellum gene cluster as well as the genes for chemotaxis, cell shape regulation and likely acts in the same regulatory network as CirA. Since archaellum driven motility represents an adaptive behavior that enables cells to move away from unfavorable conditions and given that the rod to plate transition controlled by CsmR is triggered by environmental cues such as increasing population densities and quorum sensing signals, we propose that CsmR provides a new connection between motility and cell morphology in archaea. These discoveries offer fresh insight into archaeal regulatory networks and establish a new paradigm for archaellum control beyond EarA-dependent systems in Euryarchaeota.

## 2. Results

### 2.1 CsmR is a novel potential regulator of archaellation in *H. volcanii*, distinct from EarA

The gene encoding CsmR (*hvo_1209*), a small 13 kDa protein, is located upstream of the archaellin genes *arlA1* and *arlA2* in the *H. volcanii* genome. The presence of a predicted winged-helix DNA-binding domain suggested that similar to the previously identified positive regulators EarA in *M. maripaludis* and *P. furiosus* [22,25], this protein would be involved in the regulation of the archaellum cluster expression in *H. volcanii*. However, homology searches revealed no sequence or structural similarity between CsmR and EarA, suggesting a distinct regulatory mechanism in *H. volcanii*. Phylogenetic distribution analysis showed that *csmR* is predominantly found in Halobacteria and some members of Thaumarchaeota (S1A Fig). Moreover, gene cooccurrence investigation showed that *csmR* always cooccurs with the archaellin genes *arlA1* and *arlA2* in Halobacteria but not in Thaumarchaeota hinting to another regulatory function in the latter phylum than motility control (S1B Fig). Structural modeling classified CsmR within the Lrp/AsnC family of transcriptional regulators, known for their roles in metabolic and stress-response pathways in archaea and bacteria [38,39]. The highest prediction of confidence of the AlphaFold 3 model supported dimer formation (pTM 0.79), consistent with the dimeric nature of Lrp/AsnC regulators. The predicted dimerization interface of CsmR is mediated by a β-sandwich fold, further aligning it with Lrp/AsnC regulators rather than the previously characterized archaellum regulators (S2 Fig).

### 2.2 Deletion of *csmR* results in the formation of elongated cells and a complete loss of motility whilst overexpression renders cells to be hypermotile and constantly rod-shaped

**Fig 1:**
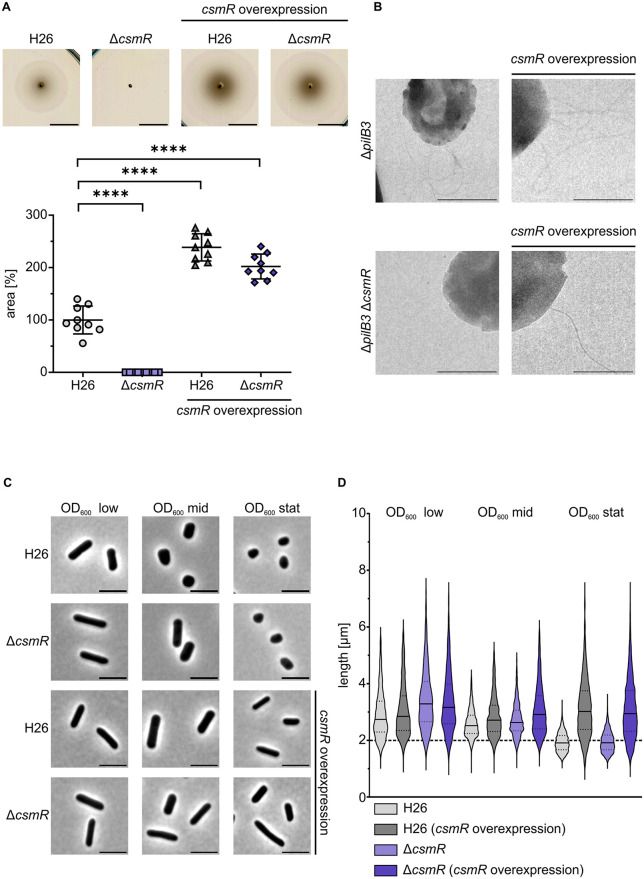
Deletion of *csmR* leads to a complete loss of motility whilst overproduction results in hypermotility. **(A)** Motility assay comparing wildtype H26 with the *csmR* deletion strain (transformed with pTA1392) and both strains overexpressing *csmR* (transformed with pSVA13922). Exemplary motility halos of all strains are shown. The area of the motility halos was measured and normalized to the average area of wildtype halos showing a significant reduction (p ≤ 0.0001) in cell locomotion of the *csmR* deletion strain compared to H26 and a significant (p ≤ 0.0001) increase of motility by *csmR* overproduction. Samples were measured in biological and technical triplicates and all nine single data points per strain were plotted. The middle line indicates the mean and the upper and lower line the standard deviation. Scale bar 2 cm. **(B)** Transmission electron microscopy images of the *pilB3* + pTA1392 and the *pilB3 csmR* + pTA1392 double deletion strain (left) and of both strains overexpressing *csmR* from pSVA13922 (right). The filament of the archaellum is not assembled in the double deletion strain whilst overexpression of *csmR* resulted in assembly of an archaellum filament in the double deletion strain. Scale bar 1 µm. **(C)** Cell shape analysis of the wildtype H26 + pTA1392 and the *csmR* + pTA1392 deletion strain at low, mid and stationary OD_600_ and under *csmR* overexpression condition. Both strains show a transition from rod- to plate-shaped cells. In contrast both overexpression strains stay rod-shaped over the whole growth cycle and do not transition from rod- to plate shaped cells. Scale bar 4 µm. **(D)** Cell shape was analyzed using MicrobeJ and the summarized results of three independent biological replicates per strain and OD_600_ value plotted as violin-plots. The median is indicated by the middle black line, dotted lines indicate the first and third quartile. For each condition more than 1000 cells were analyzed. The dotted line indicates the length below which cells are considered plate shaped.

To investigate the function of the potential archaellum regulator in *Haloferax volcanii* we generated a marker less deletion strain of *csmR,* that grows like the H26 control (S3A Fig) and assessed a potential effect on cell locomotion via motility plates. For all experiments strains, if not mentioned differently, were transformed with plasmid pTA1392 to complement the uracil auxotrophy promoting more reliable cell growth and a more homogeneous cell shape. In comparison to the control strain H26 the *csmR* deletion strain fully lost the ability to swim on semi solid agar plates (Fig 1A) indicating that *csmR* controls archaellum expression. Indeed, transmission electron microscopy of a Δ*pilB3*Δ*csmR* strain showed cells completely devoid of archaellum filaments (Fig 1B), whilst the Δ*pilB3* control strain showed archaellation. Since the discrimination between pili and archaellum filaments due to their homology is difficult, a deletion strain of the pilin assembly ATPase PilB3 [40] of *H. volcanii* was used to obtain cells that only have archaella as cell appendages. Uncropped TEM images are provided in the supplementary information (S4 Fig).

Several studies showed that motility of *H. volcanii* strictly depends on its cell shape [28]. *Haloferax volcanii* is pleomorphic being rod shaped and motile in lag- growth phase whilst during the exponential growth phase cells undergo a morphologic transition to plate shaped cells that are not motile anymore even though the archaellum motor is still present [28]. To exclude the option that the *csmR* deletion strain is immotile due to cell shape effects, we imaged cells of the deletion strain at different growth stages and compared them to strain H26 (early (OD_600_ of 0.02), mid (OD_600_ of 0.2), and stationary growth (OD_600_ of 2.0)). Both strains showed the typical cell shape of *H. volcanii* during growth, with rod-shaped cells at early growth stages and a transition to plate-shaped cells when they reach the stationary phase. However, Δ*csmR* cells were more elongated during growth than H26 cells hinting to a regulatory function of CsmR not only in cell locomotion but also cell shape determination (Fig 1C and 1D). To exclude a potential effect on cell shape and motility of the investigated strains due to the transformation with pTA1392 experiments were also conducted in absence of the plasmid with the exact same result as with plasmid (S3B and S3C Fig).

Overexpression of the euryarchaeal archaellum regulator (EarA) in other Euryarchaeota led to drastically increased archaellum formation [25]. To test if overexpression of CsmR in *H. volcanii* has a similar effect, H26 and the *csmR* deletion strain were transformed with a plasmid that harbored *csmR* under the control of a strong xylose inducible promoter [41]. Assessment of the optimal inducer concentration showed that the *csmR* deletion phenotype, in regard to motility, is already complemented without addition of xylose. Derived from motility assays under induced and non-induced conditions, we assume that under non-induced conditions, CsmR synthesis is very low (S5A Fig) suggesting that relatively low CsmR concentration already have an impact on the cell. The more the inducer concentration was increased the more strains transformed with the overexpression plasmid showed motility on the plates (S5B and S3C Figs). To obtain maximum expression of *csmR* 12 mM xylose was used for induction. In the Δ*csmR* strain overexpression complemented the non-motile phenotype of the deletion strain not only back to wildtype levels but caused hypermotility (Fig 1A). The same was observed when *csmR* was overexpressed in H26, causing motility halos more than twice the size of the motility halo of the control strain. Increased cellular protein concentration of EarA in *P. furiosus* cells lead to hyperarchaellation [25]. However, in contrast to *P. furiosus*, Δ*pilB3 H. volcanii* cells transformed with the *csmR* overexpression plasmid showed no hyperarchaellation phenotype. The Δ*pilB3*Δ*csmR* double deletion strain assembled archaellum filaments upon *csmR* overexpression, as observed with the transmission electron microscope (Fig 1B); however, the number of archaella was comparable to the control strains with or without *csmR* overexpression plasmid (Fig 1B).

As mentioned before, cell motility of *H. volcanii* strongly depends on its cell shape. Whilst the control strain H26 and the *csmR* deletion strain transition from rod to a plate-like shape during growth, both strains are locked in a rod-shaped appearance upon *csmR* overexpression (Fig 1C and 1D). Therefore, CsmR not only seems to affect archaellum expression but possibly also regulates genes that are involved in rod formation and/or maintenance.

### 2.3 Cell shape transition does not alter membrane lipid composition

To ascertain if morphological changes impact the composition of membrane lipid types, the abundance of several lipid species present in *H. volcanii* was assessed during different growth phases (S6 Fig). Overall, only small changes were observed, which predominantly involve the lower abundant lipid species PA (phosphatidic acid), PE (phosphatidylethanolamine), MGD (monogalactosyldiacylglycerol), as well as the cardiolipins. The only major abundant lipid species that seems to be mildly influenced by the growth stage is PG (phosphatidylglycerol). This might be explained by the fact that PG serves as a substrate for all cardiolipins, which in turn seem to be slightly upregulated at later growth stages, which is a common phenomenon observed in prokaryotes. Next, analysis of the lipid tail distribution of the four most abundant lipid species in *H. volcanii* was performed (S7 Fig). C40:0 (i.e., two times a phytanyl chain) is the predominantly observed form, which is in accordance with the literature [42]. Other lipid tail configurations do exist, in which both the level of desaturation as well as the carbon chain length can differ. In general, there seems to be a trend that with increasing optical densities, there is a decrease in saturated lipid tails, except for PG. Again, this might be explained by the fact that unsaturated PG serves a substrate for cardiolipins, which is more prominently produced during later growth stages. Altogether though, the overall changes in lipid composition (headgroup as well as tail configuration) are minor. Hence, it is unlikely that changes in cell morphology during growth are caused by an altered lipid composition.

### 2.4 *CsmR* deletion induces widespread upregulation of archaellum and chemotaxis genes

**Fig 2:**
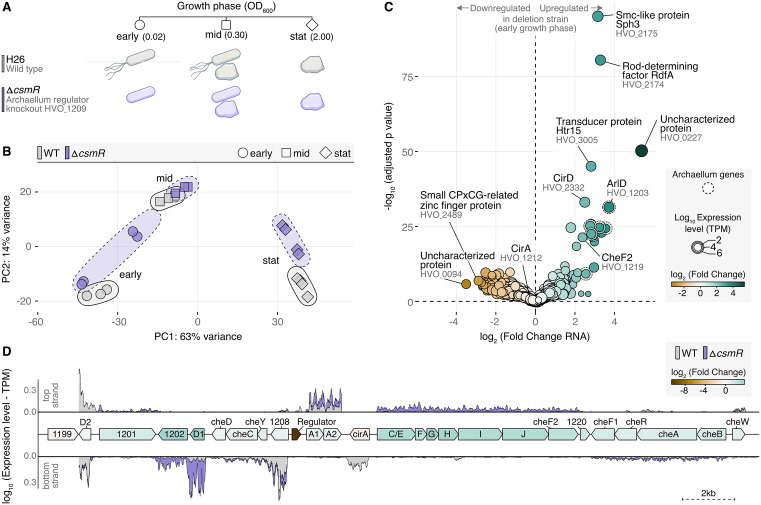
Deletion of *csmR* leads to upregulation of archaellum and chemotaxis genes. **(A)** Schematic of the experimental design, comparing the *Haloferax volcanii* wild type strain H26 and a deletion strain of the potential archaellum regulator CsmR, across different growth phases. Note that during normal growth conditions, *Haloferax* exhibits pleiomorphism, transitioning from rod-shaped, motile cells to sessile plate-like cells. **(B)** Principal component analysis from wild type (WT, grey) and ∆*csmR* (archaellum regulator, purple) strains, based on total read counts after outlier removal. Different growth phases are indicated by different cell shapes, illustrating the primary source of transcriptomic variance. **(C)** Volcano plot showing differential gene expression in low OD samples. Genes are represented as circles, with log_2_-fold changes color-coded from dark brown (downregulated in deletion strain) to dark green (upregulated in deletion strain), expression levels indicated by circle size and archaellum-related genes highlighted with dashed outlines. **(D)** Mean coverages of strand-specific, TPM (transcripts per million)-normalized reads are shown for wild type (grey) and *csmR* deletion strain (purple). The middle tracks display archaellum and chemotaxis-related genes, according to their relative gene size (scale bar depicted). Genes are color-coded based on log_2_-fold changes from dark brown (downregulated in deletion strain) to dark green (upregulated in deletion strain) under low-OD conditions and strand orientation indicated by rectangle direction.

To further investigate how CsmR regulates motility and cell shape transitions, we analyzed its impact at the transcriptional level. We performed RNA-sequencing on both wild type and ∆*csmR* strains under three distinct growth conditions to: (1) assess the overall transcriptional effect of *csmR* deletion and uncover potentially associated regulatory networks and (2) determine whether these effects vary under conditions where cells typically lack appendages and adopt a plate-like morphology (Fig 2A).

Principal component analysis revealed that the growth phases of the harvested cultures were the primary driver of transcriptomic variation, while the deletion of *csmR* had only a smaller impact. This supports the hypothesis that CsmR functions as a dedicated regulator of archaellation rather than on a global scale (Fig 2B). To further investigate gene-specific effects, we performed differential gene expression analysis across the growth phases (early (OD_600_ of 0.02), mid (OD_600_ of 0.2), and stationary growth (OD_600_ of 2.0)). In early exponential growth, ∆*csmR* cells showed significant upregulation of shape-relevant genes, including *sph3* (*hvo_2175*) and r*dfA* (*hvo_2174*), as well as chemotaxis-associated genes (Fig 2C). Despite the complete loss of motility, RNA-seq analysis revealed a significant upregulation of archaellum genes in ∆*csmR* (Fig 2C)*. H. volcanii* features a unique gene arrangement, where archaellation and chemotaxis genes are interspersed and transcribed in different orientations. Despite this organization, ∆*csmR* exhibited consistent upregulation across entire transcriptional units, including ArlA1/A2, ArlC/E/F/G/G/I/J/CheF2, CheB/A/R/F1, CheY/C/D, and ArlD/HVO_1202 (Fig 2D). This contrasts with the expected results, given the strain’s impaired swimming ability, and deviates from regulatory patterns observed in other euryarchaeal archaellum regulators [24,25]. Rather than implying that CsmR acts as a direct transcriptional repressor of these genes, this paradoxical upregulation suggests a more complex regulatory mechanism in which CsmR may function as a regulator whose absence indirectly derepresses archaellum gene expression, possibly through dysregulation of counterbalancing regulators.

### 2.5 CsmR deletion leads to persistent activation of motility and signalling pathways across growth phases

**Fig 3:**
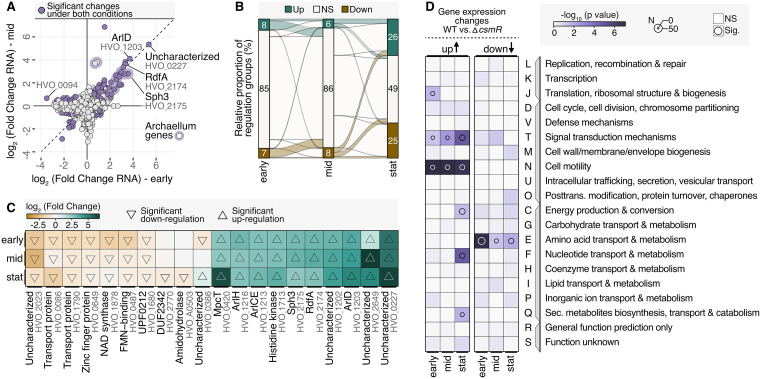
C*smR* deletion leads to a shared functional response across growth conditions. **(A)** Scatter plot showing the log_2_-fold changes in gene expression between early and mid-exponential samples for the ∆*csmR* strain relative to the wild type. Archaellum genes (dashed outline, light pink) and other genes that are consistently regulated under both conditions are highlighted. Genes with significant changes (adjusted *P*-value < 0.05) under both conditions are colored in pink. **(B)** Flow diagram illustrating the relative number and interconnectedness of genes between all growth conditions (x-axis) for each regulatory group (upregulated: green, downregulated: brown, rest: off-white). **(C)** Heatmap displaying the top 10 genes with the highest mean log_2_-fold changes across all conditions. Significance (adjusted *P*-value < 0.05) is indicated by triangles, with log_2_-fold changes color-coded from dark brown (downregulated in deletion strain) to dark green (upregulated in deletion strain). **(D)** Gene set enrichment analysis of archaeal clusters of orthologous groups (arCOGs) across all three growth conditions. Color intensity (white to dark purple) indicates **-**log10(raw overrepresentation P-value), while circles denote categories with raw overrepresentation P-value < 0.05. Circle size reflects the total number of differentially regulated genes assigned to each category. These enrichment results are intended as exploratory.

To assess the impact of the *csmR* deletion across different growth conditions, we next examined differential gene expression in mid and stationary samples. Pearson’s correlation analysis (R = 0.27) revealed a weak positive correlation between log₂-fold changes in early and exponential (mid) growth phases, indicating that only a subset of genes was consistently up- or downregulated across both conditions (Fig 3A). Notably, several of the most upregulated genes in early growth, particularly those linked to archaellation and cell shape regulation, remained upregulated in the exponential phase. Overall, during early phase, 8% of genes were upregulated and 7% downregulated, with similar proportions in the exponential phase (6% upregulated, 8% downregulated), though there was minimal overlap between these gene sets (Fig 3B). In the stationary phase, where cells adopt a different morphology, gene regulation was significantly more pronounced, with 26% of the genes upregulated and 25% downregulated. Notably, archaellation and chemotaxis-associated genes remained consistently upregulated across all growth phases (Fig 3C). However, no clear functional pattern among downregulated genes could be detected. To further investigate the biological significance of these transcriptional changes, we performed functional enrichment analysis using archaeal clusters of orthologous groups (arCOGs). This analysis revealed overrepresentation trends in groups N (cell motility) and T (signal transduction mechanisms) among upregulated genes across all growth phases. Additionally, categories Q (secondary metabolites biosynthesis, transport, & catabolism), F (nucleotide transport & metabolism), and C (energy production & conversion) were enriched in stationary phase, while category E (amino acid transport & metabolism) was consistently underrepresented among downregulated genes across all conditions (Fig 3D).

### 2.6 CsmR directly binds promoter regions of motility-associated genes

**Fig 4:**
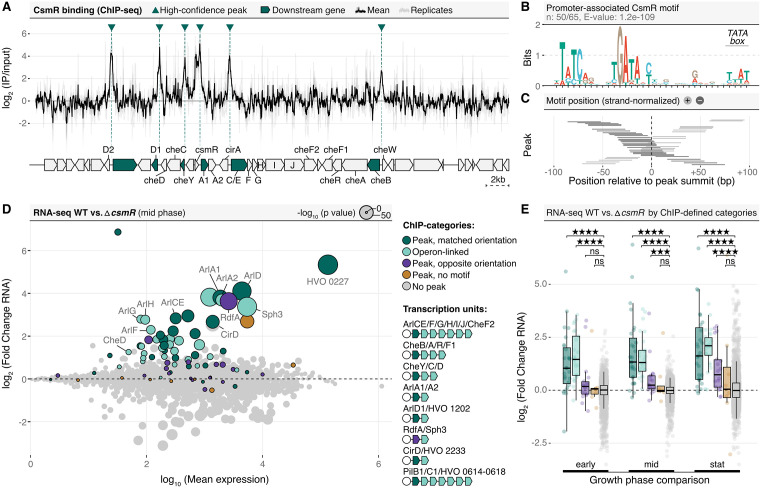
CsmR binds promoter regions of motility-associated genes and is linked to transcriptional changes. **(A)** Mean log₂(ChIP/Input) signal across the *arl* operon region showing CsmR enrichment at promoter-proximal sites. Individual replicate ratios are shown in grey. Peak summits are indicated by vertical lines, and gene annotations are shown below according to genomic orientation. **(B)** Sequence logo of the CsmR binding motif identified by *de novo* motif analysis of peak-associated regions. The semi-palindromic consensus sequence TATCA(N₄)TGATA is enriched upstream of target genes and positioned relative to the archaeal TATA box. **(C)** Strand-normalized positions of motif matches relative to ChIP-seq peak summits, showing that the CsmR motif occurs in a defined position near the centre of enriched regions. **(D)** Integration of ChIP-seq and RNA-seq data. Differential gene expression in the ∆*csmR* strain is shown as log₂ fold change versus expression level. Genes associated with CsmR peaks are highlighted and grouped based on binding characteristics (motif presence, strand orientation, and promoter distance). **(E)** Comparison of log₂ fold changes across gene classes. Genes associated with motif-containing, promoter-proximal, strand-consistent CsmR peaks show the strongest upregulation in the ∆*csmR* strain, followed by genes within the same transcriptional units. Statistical significance was assessed using Wilcoxon rank-sum tests.

To distinguish between direct and indirect regulatory effects and to identify genomic binding sites of the potential global regulator CsmR, we performed chromatin immunoprecipitation followed by sequencing (ChIP-seq). Distance analysis of peak summits relative to annotated gene start positions revealed a strong enrichment of CsmR binding sites in close proximity to gene starts, consistent with promoter-proximal binding (S8A Fig). In total, 65 genes were associated with high-confidence CsmR peaks, with the majority of sites located in intergenic regions (41 intergenic vs. 24 intragenic). Inspection of individual loci revealed pronounced CsmR enrichment at promoter regions of genes associated with archaellation and chemotaxis, including the arl operon (Figs 4A and S8B).

*De novo* motif analysis identified a ~ 34 bp promoter-proximal sequence containing a semi-palindromic consensus motif, TATCA(N₄)TGATA, present in 50 of 65 peaks. Interestingly, adjacent Canonical archaeal promoter elements like the TATA box are located adjacent to the CsmR binding site (Fig 4B). Comparison of the core CsmR motif to known transcription factor binding sites did not reveal significant similarity to previously described Lrp/AsnC motifs. After accounting for motif orientation, these sequences showed a consistent positional relationship relative to peak summits (Fig 4C).

Integration of ChIP-seq and transcriptome data showed that genes strongly upregulated in the ∆*csmR* mutant were predominantly associated with promoter-proximal CsmR peaks or belonged to transcriptional units containing such peaks (Fig 4D). Genes with motif-containing, strand-consistent peaks at promoter-compatible distances exhibited the strongest upregulation, followed by operon-linked genes, whereas genes without detectable binding showed little or no change (Fig 4E). These differences were statistically significant across all tested conditions. Direct CsmR targets showed consistent growth-phase-dependent expression patterns, with similar trends in wild type and ∆*csmR* strains, while CsmR transcript levels remained stable across all conditions, suggesting that its activity is not primarily controlled at the transcriptional level (S9 Fig).

Following the identification of the CsmR binding sites by ChIP-seq, we further investigated its structural features to assess whether they are consistent with DNA-binding and with membership in the Lrp/AsnC family of transcription factors. Structure-guided comparisons with characterized Lrp/AsnC family members, including *E. coli* Lrp/AsnC and *B. subtilis* LrpC, showed that the strongest structural conservation is confined to the predicted N-terminal DNA-binding region of CsmR, whereas the remaining part of the protein is more divergent (S10 Fig). Thus, rather than displaying close full-length similarity to canonical Lrp/AsnC regulators, CsmR appears to retain a conserved DNA-binding core within an otherwise divergent architecture. In addition, Foldseek identified the *P. furiosus* Phr regulator (PDB ID: 2P4W) as a structurally related hit, further supporting similarity in the overall fold and arrangement of the predicted DNA-binding region (S11 Fig). Consistently electrostatic surface mapping identified a positively charged surface patch in this region, compatible with a DNA binding interface (S12 Fig). A multiple sequence alignment (MSA) across haloarchaeal homologs further showed that this N-terminal region is the most conserved part of the protein and contains several conserved basic residues, supporting its role in direct transcriptional regulation (S13 Fig). Analysis with the Protein Structure Transformer (PeSTo) tool [43] likewise predicted a DNA binding interface in this conserved and positively charged region (S14 Fig). In contrast, the remainder of CsmR showed substantially lower sequence conservation, consistent with a more divergent family associated region that may contribute to oligomerization or regulatory protein interactions rather than sequence specific DNA recognition (S14 Fig).

### 2.7 CsmR and CirA co-regulate archaellation and chemotaxis through overlapping but distinct pathways

**Fig 5:**
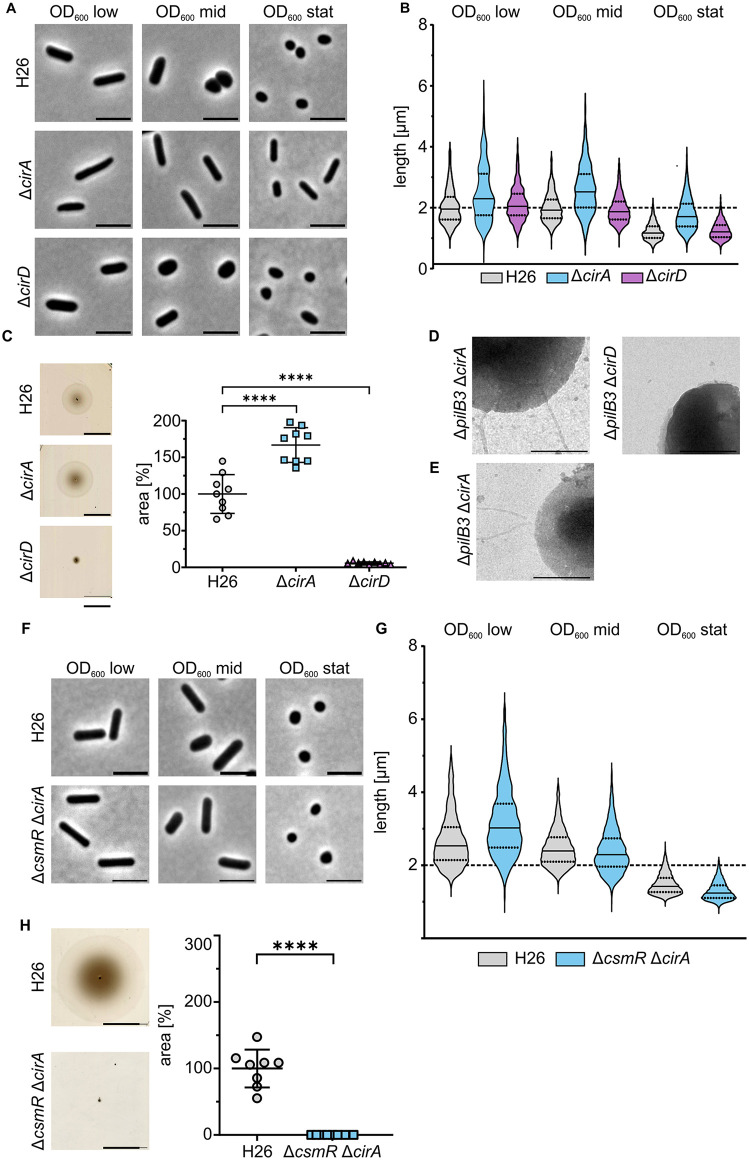
CirA and CirD impact cell locomotion of *H. volcanii* but the *csmR* deletion phenotype is dominant over the Δ*cirA* phenotype. **(A)** Cell shape analysis of the wildtype H26 + pTA1392, the *cirA* + pTA1392 and *cirD* + pTA1392 deletion strain at low, mid and stationary OD_600_. The *cirA* deletion strain stayed rod-shaped over the whole growth cycle and did not transition from rod- to plate shaped cells as the control strain or the *cirD* deletion strain. Scale bar 4 µm. **(B)** Cell shape was analyzed using MicrobeJ and the summarized results of three independent biological replicates per strain and OD_600_ value plotted as violin-plots. The median is indicated by the middle black line, dotted lines indicate the first and third quartile. For each condition more than 1000 cells were analyzed. The dotted line indicates the length below which cells are considered plate shaped. **(C)** Motility assay comparing wildtype H26 + pTA1392 with the *cirA* + pTA1392 and *cirD* + pTA1392 deletion strain. Exemplary motility halos of the three strains are shown. The area of the motility halos was measured and normalized to the average area of wildtype halos showing a significant reduction (p ≤ 0.0001) in cell locomotion of the *cirD* deletion strain compared to H26. In contrast the deletion strain of *cirA* showed increased (p ≤ 0.0001) motility compared to the wildtype. Samples were measured in biological and technical triplicates and all nine single data points per strain were plotted. The middle line indicates the mean and the upper and lower line the standard deviation. Scale bar 2 cm. **(D)** Transmission electron microscopy images of the *pilB3 cirA* and the *pilB3 cirD* double deletion strains showing that the *cirA* deletion strain is archaellated while the *cirD* deletion strain is not. Scale bar1 µm. **(E)** Transmission electron microscopy images of the *pilB3 cirA* double deletion strain at the stationary growth phase being still archaellated. Scale bar 1 µm. **(F)** Cell shape analysis of the wild type H26 + pTA1392 and the *csmR cirA* + pTA1392 double deletion strain at low, mid and stationary OD_600_. Scale bar 4 µm. **(G)** Cell shape was analyzed using MicrobeJ and the summarized results of three independent biological replicates per strain and OD_600_ value plotted as violin-plots. The median is indicated by the middle black line, dotted lines indicate the first and third quartile. For each condition more than 1000 cells were analyzed. The dotted line indicates the length below which cells are considered plate shaped. **(H)** Motility assay comparing wild type H26 + pTA1392 with the *csmR cirA* + pTA1392 double deletion strain. Exemplary motility halos of both strains are shown. The area of the motility halos was measured and normalized to the average area of wild type halos showing significantly (p ≤ 0.0001) decreased motility in the double deletion strain compared to the H26 control. Samples were measured in biological and technical triplicates and all single data points per strain were plotted. The middle line indicates the mean and the upper and lower line the standard deviation. Scale bar 2 cm.

Given that *csmR* deletion resulted in widespread transcriptional upregulation of archaellation and chemotaxis genes, we next sought to identify additional regulatory factors that may interact with or modulate CsmR function. In particular, *cir* genes (*cirA*/ *cirD*) emerged as key candidates, given their established role in archaella regulation and the observed transcriptional shifts in Δ*csmR*. To further explore these connections, we examined *cir* gene expression patterns in Δ*csmR.* In the wild type, transcript levels for *cirA* and *cirD* were comparable across growth phases. However, in the ∆*csmR* strain, *cirD* exhibited consistent upregulation across all phases, while *cirA* showed reduced expression in early and stationary phases but remained unchanged during mid-exponential conditions (S15 Fig). To further analyze their roles, we generated ∆*cirD* and ∆*cirA* deletion strains. Phenotypically, ∆*cirD* resembled ∆*csmR,* with non-motile cells and shape dependency, while ∆*cirA* displayed hypermotility as previously described [33] and a persistent rod-like morphology across all growth phases (Fig 5A–5C). Additionally, transmission electron microscopy revealed archaellation of the *cirA* deletion strain even at stationary growth phase in contrast to the *cirD* deletion strain that was not archaellated at all (Fig 5D and 5E). Notably, a double deletion strain of ∆*csmR* ∆*cirA* resulted in a phenotype identical to ∆*csmR* alone, suggesting that CirA acts upstream or in parallel to CsmR in motility regulation (Fig 5F–5H). With respect to cell shape the double deletion of *csmR cirD* showed no difference to the wild type. However, the ∆*csmR* ∆*cirD* strain showed slight motility (S16A–S16C Fig). This was surprising as both single deletions were fully non-motile. Despite these morphological differences in ∆*cirA*, growth phase remained the primary driver of transcriptomic variance across all strains (S17A and S17B Fig). However, ∆*cirA* exhibited significantly more deregulated genes during early and mid-exponential growth, whereas ∆*csmR* and ∆*cirD* displayed stronger transcriptomic shifts during stationary phase (S18 Fig). Gene set enrichment analysis revealed that ∆*cirA* and ∆*csmR* share similar functional effects, particularly among upregulated genes during early and mid-exponential phases (S19 Fig). However, in stationary phase, ∆*csmR* more closely resembled ∆*cirD*, except for motility-related genes, which were not enriched in ∆*csmR*. Focusing on CsmR targets identified through ChIP-seq, we found that ∆*cirA* deletion led to strong upregulation on the respective genes, whereas ∆*cirD* had minimal impact (S20 Fig). While overall expression correlations between the strains were moderate, CsmR target genes were among the most differentially expressed genes in both ∆*cirA* and ∆*csmR* strains (S17C Fig). Notably, despite high correlation between ∆*csmR* and ∆*cirD* during stationary phase, chemotaxis and archaellin genes showed distinct regulation (S21A Fig). Using publicly available Δ*rosR* transcriptomic data, we did not observe transcriptional changes associated with archaellation that correlated with the patterns seen for Δ*csmR*, Δ*cirA*, or Δ*cirD* (S21B and S12C Fig). To validate these findings, we analyzed the data independently of the previously identified promoter CsmR targets and detected 49 genes significantly upregulated in ∆*csmR* and ∆*cirA* across all growth phases (padj < 0.1) (S17D Fig). STRING network analysis revealed a highly interconnected cluster of archaella assembly, chemotaxis, and signaling genes (S17E Fig). This core included well-characterized genes such as ArlA, ArlB, ArlC, and CheF, alongside transcriptional regulators like TFB and CirA. Interestingly, pilin genes were upregulated in ∆*csmR* and ∆*cirA*, suggesting a compensatory shift towards type IV pili-related functions, such as surface adhesion, in response to disrupted archaella regulation. Glycoproteins and signaling proteins were also linked to this network, reinforcing the influence of both CsmR and CirA on chemotaxis and cellular signaling. Notably, CirD-regulated genes were absent, highlighting its distinct and phase-specific role.

### 2.8 Posttranscriptional regulation of *cirA* by the RNA *hvo_1211s*

Since CirA appears to be prominently involved in archaellum and cell shape regulation we had a closer look at its genetic neighborhood. Very recently, the transcriptional regulator RosR was shown by ChIP-seq to bind to the 3’ end of the *arlA2* gene that is located downstream of *cirA* [44]. Interestingly, the cells in the Δ*rosR* mutant were also non-motile ([44], S22A and S22B Fig) most likely due to the disability to form rod-shaped cells (S22C–S22E Fig) that is essential for *H. volcanii* to swim. Northern blot analysis of RNA isolated from the *rosR* deletion strain showed enriched *cirA* RNA levels compared to the wildtype (S23A and S23B Fig). This might explain the disability of Δ*rosR* cells to form rod shaped cells that are motile since CirA is involved in the formation of plate-shaped cells. Analysis of transcription start site data at OD_600_ of 0.8 under standard growth conditions [45] revealed a transcription start site downstream of the 3’ end of *arlA2* and the RosR binding site. Northern blot analysis confirmed the expression of an RNA with a size of about 420 nt (S23C Fig). We term this hitherto unannotated gene *hvo_1211s*. With its 420 nt the gene reaches from the intergenic region between *arlA2* and *cirA* into the 3’ end of *cirA* (S24 Fig). Since the *hvo_1211s* RNA is complementary to almost half of the *cirA* RNA it has the potential to act as an anti-sense RNA to regulate *cirA* translation or mRNA stability. To test this hypothesis, we deleted the part of the *hvo_1211s* RNA in the intergenic region leaving *cirA* intact.

**Fig 6:**
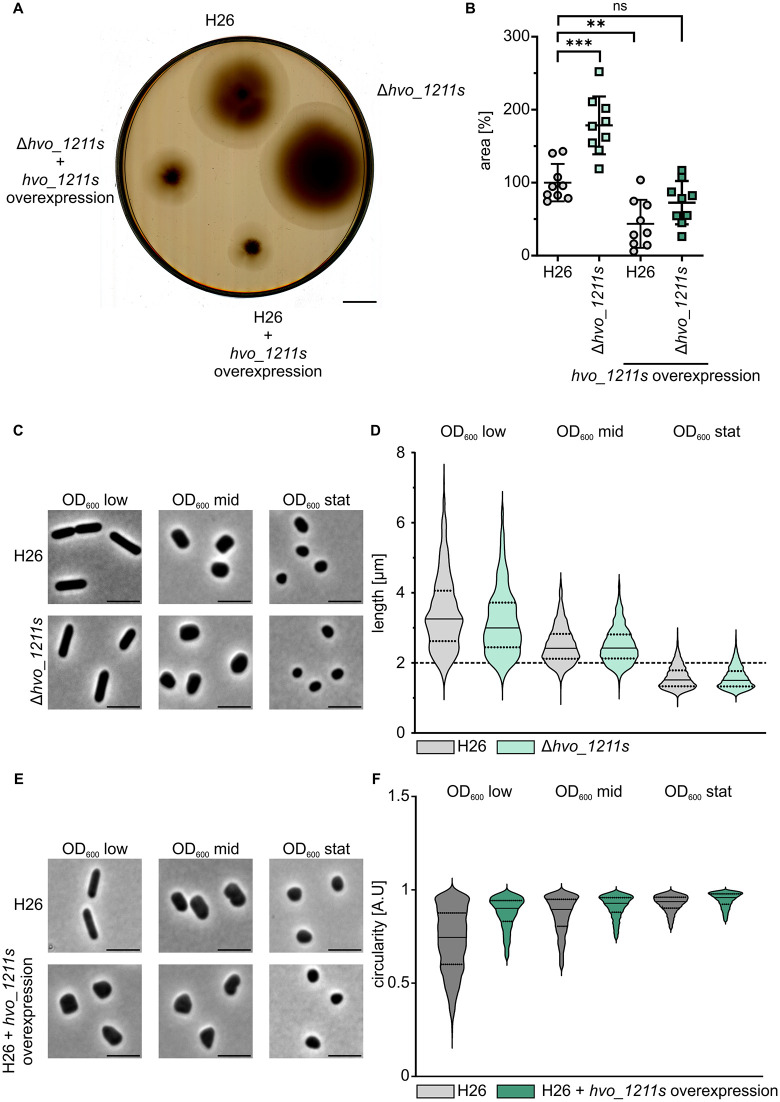
Motility of *H. volcanii* is suppressed by a regulatory RNA. **(A)** Motility assay comparing wild type H26 with the *hvo_1211s* deletion strain either transformed with pTA1392 or a plasmid for overexpression of *hvo_1211s*. An exemplary motility plate is shown. Scale bar 2 cm **(B)** The area of the motility halos was measured and normalized to the average area of wild type halos showing significantly (p ≤ 0.05) increased motility in the *hvo_1211s* deletion strain compared to the H26 control. Overproduction of *hvo_1211s* suppresses motility in H26 significantly compared to the wild type control (p ≤ 0.05) and fully complements the hypermotility phenotype of the Δ*hvo_1211s* strain back to wild type levels. Samples were measured in biological and technical triplicates and all single data points per strain were plotted. The middle line indicates the mean and the upper and lower line the standard deviation. **(C)** Cell shape analysis of the wild type H26 + pTA1392 and the *hvo_1211s* + pTA1392 deletion strain at low, mid and stationary OD_600_. The *hvo_1211s* deletion strain showed the same phenotype as the control strain. Scale bar 4 µm. **(D)** Cell shape was analyzed using MicrobeJ and the summarized results of three independent biological replicates per strain and OD_600_ value plotted as violin-plots. The median is indicated by the middle black line, dotted lines indicate the first and third quartile. For each condition more than 1000 cells were analyzed. The dotted line indicates the length below which cells are considered plate shaped. **(E)** Cell shape analysis of H26 with and without an *hvo_1211s* overexpression plasmid at low, mid and stationary OD_600_. Overexpression of *hvo_1211s* inhibits rod formation of the cells. Scale bar 4 µm. **(F)** Cell circularity was analyzed using MicrobeJ and the summarized results of three independent biological replicates per strain and OD_600_ value plotted as violin-plots. The median is indicated by the middle black line, dotted lines indicate the first and third quartile. For each condition more than 1000 cells were analyzed.

Similar to ∆*cirA,* partial deletion of *hvo_1211s* showed a hypermotile phenotype compared to the wild type (Fig 6A and 6B). However, unlike in the *cirA* deletion strain cell shape transition during growth was not affected (Fig 6C and 6D). Overexpression of the full length *hvo_1211s* blocked rod formation during early growth and hampered motility in the wildtype and partially in the *hvo_1211s* deletion strain (Fig 6E and 6F), similar to the *rosR* deletion strain (S22 Fig). Hence, the strain carrying a deletion of *rosR* and the strain with *hvo_1211s* overexpression show a comparable phenotype suggesting an inhibitory function of RosR on *hvo_1211s* transcription that in turn might have a promoting effect on *cirA* transcript abundancy as detected by Northern blotting (S23A and S23B Fig). Additionally, to exclude that the hypermotility phenotype of ∆*cirA* is a result of the partial deletion of *hvo_1211s,* we generated a partial deletion of *cirA* leaving *hvo_1211s* full sequence intact. The partial *cirA* deletion showed the same phenotype as the full *cirA* knock-out. Cells were rod-shaped throughout the complete growth cycle and hypermotile (S25A–S25D Fig). This implies that the cause for the hypermotility of Δ*cirA* is its constant rod-shaped cell shape whilst the cause of the hypermotility of Δ*hvo_1211s* might be a direct impact on the archaellum operon since the deletion strain kept its cell shape like the wild type. Taken together, it is possible that *hvo_1211s* might act as a regulatory RNA that impacts archaellum genes thereby adding an additional layer of post-transcriptional regulation on cell shape and motility.

## 3. Discussion

### 3.1 Summary

This study identifies CsmR as a novel transcriptional regulator integrating motility and cell shape control in *H. volcanii*. Our findings establish CsmR as essential for archaellation, connecting its function to both the transcriptional regulation of motility genes and the morphological transitions during growth. Unlike previously described archaellum regulators, which typically activate archaellation under favorable conditions [24,25], CsmR deletion leads to a paradoxical upregulation of archaellum and chemotaxis genes, despite the complete loss of motility. This paradox is best explained by the integration of ChIP-seq, transcriptomic and genetic data, which show that CsmR binds directly to promotor regions of archaellum-associated genes, while the increased transcript levels observed in Δ*csmR* most likely reflect regulatory network effects involving the CirA axis rather than direct transcriptional repression by CsmR itself. Importantly, while motility and cell shape are functionally linked in *H. volcanii* under standard growth conditions, archaellation and cell morphology are not obligatorily coupled processes across haloarchaea. Reports from other species demonstrate that round or plate-shaped cells can retain archaellum filaments [46] and the relationship between cell shape and archaellation described in this study should therefore be understood as reflecting the specific biology of *H. volcanii* rather than a general principle of haloarchaeal motility.

### 3.2 Transcriptional control and evolutionary context

A defining feature of *H. volcanii* archaellum regulation is its dispersed operon organization, where motility and chemotaxis genes are interspersed and transcribed in opposing directions [10]. This contrasts with the clustered archaellum loci found in most Euryarchaeota, likely necessitating a complex regulatory framework. The extensive overlap of CsmR and CirA regulons supports a model in which CsmR acts as a central regulator, while CirA fine-tunes motility responses. The deletion of *cirA* results in hypermotility and a prolonged rod morphology ([33], our data) whereas *csmR* deletion eliminates motility despite transcriptional upregulation of archaellum genes. The *csmR*/*cirA* double deletion has the same phenotype as the ∆*csmR* mutant, reinforcing the hypothesis that CsmR occupies a dominant regulatory position, with CirA acting as a modulator rather than an independent transcriptional activator.

These findings parallel the multi-layered regulatory strategies observed in Thermoproteota, such as *S. acidocaldarius*, where motility control involves both transcriptional and post-translational mechanisms. In *S. acidocaldarius*, archaellation is regulated by ArnA and ArnB, which are modulated by phosphorylation, enabling rapid environmental responsiveness [14,16]. The potential for post translational regulation within the *H. volcanii* archaellum regulatory network is further supported by the observation that KaiC-like proteins are directly integrated into the archaellum machinery itself: ArlH, an essential archaellum motor component [7,8] is a KaiC-like protein whose ATPase and autophosphorylation activities are critical for motor assembly and function. This establishes a precedent for KaiC family proteins acting as regulatory switches within the archaellum system specifically. CirA, also a KaiC homolog, may therefore exert analogous post translational control over CsmR function, potentially through phosphorylation of the unstructured C-terminal region of CsmR, reminiscent of the phosphorylation-based regulation of archaellum regulators ArnA and ArnB in *Sulfolobus* [14–16]. Under this model CirA would function not merely as a transcriptional modulator but as post translational switch that fine tunes CsmR activity in response to growth phase signals adding an additional layer of regulation to the CsmR/ CirA axis beyond what is captured at the transcriptional level alone.

Additionally, the structural similarity of CsmR to Lrp/AsnC-type regulators - widely conserved in archaea and bacteria - suggests evolutionary parallels with bacterial motility regulators, which integrate transcriptional control with metabolic adaptations [47,48]. This is supported by a recent study already linking transcriptional regulation to metabolic control in *H volcanii*, revealing an interplay between cellular development and central metabolism. In this study TrmB and TbsP were identified as a key transcriptional regulators that influence motility and biofilm formation by controlling the glucose metabolism through co-regulation of *gapII* expression [49]. Furthermore, bacterial motility control involves both transcriptional and post-transcriptional regulation through flagellar-specific sigma factors, small RNAs, and kinases ensuring precise control over flagellum assembly, sometimes even morphology-dependent [50–55]. The identification of *hvo_1211s* includes a regulatory RNA in the archaellum and cell shape regulatory network of *H. volcanii*. Through its overlap with *cirA* - initially thought to act as an anti-sense RNA for *cirA* blocking its translation - our data suggest that it rather supports *cirA* expression resulting in increased levels of CirA that lead to the formation of plate shaped cells and the reduction of motility that is tightly coupled to cell shape. Thereby *hvo_1211s* might shield the *cirA* mRNA from endonucleolytic cleavage stabilizing the 3’ end of its target by interaction with their complementary parts supporting mRNA stability, a mechanism described for a regulatory *sRNA* in the archaeon *Methanosarcina mazei* as well [56].

Several environmental factors are known to influence cell shape and motility in *H. volcanii* and related haloarchaea, though their molecular intersection with the CsmR regulatory network remains to be established. Lower growth temperatures induce upregulation of archaellins in *H. volcanii* [27], suggesting that temperature-dependent signals feed into the archaellum regulatory network at some level. Salt concentration has been shown to influence archaellum filament composition in *Haloarcula marismortui* [26], pointing to osmotic conditions as an additional environmental input. Most directly relevant to the present study, the rod to plate transition in *H. volcanii* is triggered by increasing population density through a quorum sensing mechanism [30], which - as discussed above - likely intersects with the CsmR/CirA axis at the level of shared downstream targets. How temperature, nutrient availability and osmotic signals are sensed and transmitted to CsmR or its regulatory partners remains unknown but the Lrp/AsnC structural classification of CsmR is notable in this context, as members of this family in other organism are known to integrate metabolic and environmental signals into transcriptional responses [38,39].

The co-regulation between motility and cell shape *in H. volcanii* further emphasizes the importance of CsmR. The persistent rod morphology of Δ*cirA* cells and the normal shape transitions of Δ*csmR* cells indicate that CirA suppresses CsmR-driven motility and shape-determining pathways. This is consistent with the broader picture of morphological control in *H. volcanii*, where dedicated shape determining factors such as RdfA, which drives rod formation, and Sph3, which promotes transition to plate morphology [32], act within regulatory landscape that CsmR appears to interface with as evidence by the upregulation of *rdfA* and *sph3* in Δ*csmR* cells, observed in our transcriptomic data. The coregulation of these shape determinants alongside archaellum genes further supports the view that CsmR coordinates morphology and motility as coupled outputs rather than independent processes in this organism. The upregulation of pilin genes in both Δ*csmR* and Δ*cirA* mutants suggests regulatory crosstalk between archaella and type IV pili, potentially allowing cells to compensate for disrupted motility by shifting toward surface-associated behaviors [33,57]. Similar compensatory mechanisms have been observed in bacteria, where flagellar defects trigger alternative motility or adhesion pathways [58]. Additionally, ArlI and ArlJ have been implicated in pilus-dependent motility, hinting at deeper connections between archaellation and type IV pilin systems [33]. The ability of ArlI and ArlJ mutations to suppress pilin-related motility defects suggests a compensatory mechanism that fine-tunes appendage function, potentially through regulatory crosstalk with CsmR and CirA. Understanding how these systems interconnect will be critical in defining the full regulatory architecture of *H. volcanii* motility.

### 3.3 Model: CsmR as a central regulator of archaellation

The central paradox of this study is that deletion of CsmR abolishes motility while simultaneously increasing transcript levels of archaellum-associated genes. Integration of ChIP-seq, transcriptomic and genetic data suggests that CsmR binds directly to promoter regions of key motility genes, whereas the elevated expression observed in Δ*csmR* is more likely explained by indirect regulatory effects within the broader CsmR/CirA network.

Our findings suggest that CsmR serves as a central regulatory node within the archaellum regulatory hierarchy. The integration of ChIP-seq and RNA-seq data allows us to directly address whether these transcriptional changes reflect direct derepression of CsmR target genes or indirect compensatory responses arising from disrupted motility or signaling. Genes associated with motif-containing, strand-consistent, promotor proximal CsmR peaks, including the entire archaellum operon and chemotaxis associated genes, showed the strongest and most consistent upregulation in Δ*csmR* across all growth phases, establishing them as high confidence direct transcriptional targets. In contrast genes lacking detectable CsmR binding showed little or no expression change arguing against a broad compensatory response as the primary driver of the observed transcriptional upregulation. We therefore conclude that the transcriptional changes observed in the *csmR* deletion predominantly reflect direct derepression of CsmR bound promotors rather than indirect effects of disrupted motility or downstream signaling. Based on the position of the CsmR binding motif upstream of BRE and TATA promoter elements in CsmR-targets, it can be inferred that CsmR acts as a positive regulator under standard early growth conditions, potentially enhancing TFB recruitment to ensure proper transcription of all necessary components for archaellum assembly. This positive regulation is counter-balanced by CirA, which functions as a strong local repressor to fine-tune the system for phase-specific regulation.

**Fig 7:**
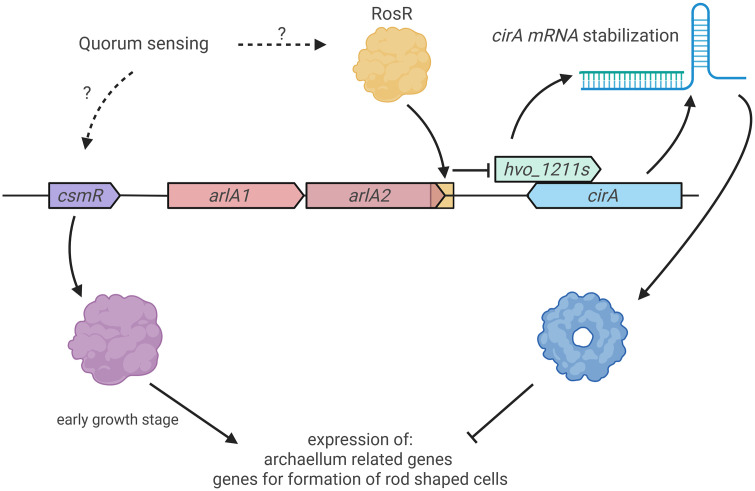
Archaellum and cell shape regulation in *Haloferax volcanii.* CsmR (purple) acts as central regulatory node within the archaellum regulatory network working as positive regulator for cell locomotion during early-stage growth when cells are motile and rod shaped. CirA (blue) counterbalances the positive regulation of CsmR during early growth, fine tuning archaellum expression and rod shape formation. CirA biosynthesis is supported by the RNA transcript *hvo_1211s* (green) stabilizing the *cirA* mRNA by interaction through their homologous regions. *Hvo_1211s* is regulated by RosR (yellow) that binds to the 3’-end of *arlA2* (yellow box) blocking transcription of *hvo_1211s*. The rod to plate transition in *H. volcanii* is triggered by a quorum sensing signal [30]. A functional intersection between the CsmR/CirA axis and the quorum sensing output pathway is proposed based on phenotypic overlap, though direct molecular evidence for this connection remains to be established. (Created in BioRender. Albers, S. (2026) https://BioRender.com/7gsc8mr).

Deletion of CirA results in hypermotility and upregulation of overlapping regulons, suggesting that CirA prevents overactivation of motility pathways by CsmR. In the absence of CsmR, CirA expression is reduced, which might derepress its targets further. We additionally propose that CirA or downstream effectors of the CsmR/ CirA regulatory axis may act at a post-transcriptional or post-translational level to block functional archaellum filament assembly in the absence of CsmR, preventing motility despite elevated transcript levels. Under this model the transcriptional upregulation of archaellum genes in Δ*csmR* reflects a decoupling of transcriptional output from assembly competence which is normally ensured by the coordinated activity of CsmR and CirA together. The ∆*csmR* ∆*cirA* double mutant phenotype, resembling ∆*csmR* alone, supports the idea that CsmR occupies a dominant position in the regulatory hierarchy, while CirA functions as a subordinate regulator. Overexpression of CsmR in this model overrides CirA’s repressive influence, resulting in archaellated constantly rod-shaped strains. *CirA*’s mRNA is influenced by the regulatory RNA *hvo_1211s* stabilizing it and *hvo_1211s* in turn is negatively regulated by RosR binding upstream of its promotor region fine tuning the interplay between cell shape transition and motility (Fig 7).

While Δ*csmR* cells retain the ability to undergo the rod to plate transition during growth, they display a consistent shape phenotype: cells are more elongated than wildtype cells at early and mid-growth phases indicating that even in the absence of CsmR the shape of rod phase cells is affected. This demonstrates that CsmR does contribute to cell shape determination in the knockout background, albeit in a subtler manner than the complete shape lock observed under overexpression conditions. We propose that this asymmetry reflects the architecture of the underlying regulatory network. In the absence of CsmR, the CirA/ *hvo_1211s*/ RosR axis remains intact and capable of driving the rod to plate transition in response to increasing population density, thus preserving the overall shape transition program despite the loss of CsmR. The elongated rod morphology of Δ*csmR* cells during early and mid-growth likely reflects dysregulation of shape determination factors, as supported by the upregulation of *rdfA* and *sph3* observed in our transcriptomic data at these growth stages. The upregulation of the rod promoting factor RdfA in particular may explain the more elongated rod morphology, as increased RdfA activity could drive exaggerated rod formation before the transition to plate shape is triggered. Under CsmR overexpression, elevated CsmR activity sustains expression of rod promoting factors and simultaneously overrides CirA mediated repression, locking cells in rod morphology regardless of growth phase and cell density. Taken together, these observations reveal a dosage dependent role for CsmR in cell shape determination: at normal expression levels CsmR fine tunes the dimensions of rod-shaped cells during early growth, while at elevated levels it overrides the shape transition machinery entirely. CsmR therefore contributes to cell shape regulation primarily through its influence on the balance of rod- and plate-promoting factors within the broader CsmR/ CirA regulatory network, rather than as a dedicated morphological regulator. Regarding the intersection between the CsmR/ CirA network and the quorum sensing system, we note that the rod to plate transition in *H. volcanii* is triggered by quorum sensing signals that accumulate with increasing population density [30]. The observation that CsmR overexpression and Δ*cirA* both result in persistent rod morphology across all growth phases, phenocopying conditions under which quorum sensing driven shape transitions are prevented, suggests functional intersection between the CsmR/ CirA regulatory axis and the quorum sensing output pathway. However, we emphasize that no direct molecular connection between CsmR, CirA or RosR and the quorum sensing machinery has been demonstrated in this study and the nature of this potential intersection remains speculative.

This hierarchical model is consistent with growth-phase-specific regulation, that is sensed by a recently described two-component system [59], enabling flexible fine-tuning of activation and repression. It also explains the stage-dependent repression of archaella-related genes, such as Sph3 and RdfA, which contribute to shape-dependent changes. The potential layered regulation by the TFB variant (HVO_0226), CsmR, CirA, *hvo_1211s*, RosR and two additional transcriptional regulators (HVO_A0465, HVO_2507) highlights the complexity of transcriptional control required for precise coordination of cell shape determination motility and archaellum assembly in *H. volcanii*.

### 3.4 Future perspectives and open questions

Our findings establish CsmR as a central transcriptional regulator coordinating motility and cell shape transitions in *H. volcanii*, yet critical questions remain. The identification of promotor-proximal ChIP-seq peaks together with a conserved consensus motif strongly supports direct DNA binding and transcriptional regulation, although the precise regulatory consequences of this binding remains to be fully resolved. Whether CsmR functions exclusively as a transcriptional activator, as a context-dependent dual-function regulator or through indirect mechanisms that vary across growth phases remains an important open question that will require further investigation. The interplay between CsmR and CirA suggests a layered regulatory system, raising the possibility that CirA modulates CsmR post-translationally, perhaps through phosphorylation. Given that phosphorylation-based archaellum regulation is well-documented in Thermoproteota, a similar mechanism in *H. volcanii* would suggest convergent strategies across archaeal lineages [60]. Beyond transcriptional control, the environmental factors governing CsmR activity remain unclear. Archaellation in *H. volcanii* is influenced by temperature and nutrient availability, suggesting that CsmR integrates external signals to balance motility and morphological transitions [27]. The connection between CsmR and other transcriptional regulators, including alternative TFBs and RosR, should be explored to map its broader regulatory network. Evolutionarily, the structural similarity between CsmR and bacterial Lrp/AsnC regulators highlights potential conserved functions linking motility and metabolism. Moreover, quorum sensing in *H. volcanii,* controlling cell morphology and motility by a small secreted molecule has recently been discovered [30]. However, the molecular mechanism by which this quorum sensing signal intersects with the transcriptional network described here, including CsmR, CirA and RosR, remains unknown and represents a priority for future investigation. The upregulation of pilin genes in Δ*csmR* and Δ*cirA* mutants further suggests regulatory crosstalk between archaella and type IV pili systems, pointing to a more integrated control of cell appendages.

By defining CsmR’s role within the archaeal transcriptional landscape, this study provides a foundation for future work on regulatory networks governing motility, environmental sensing, and cellular architecture, with broader implications for the evolution of prokaryotic transcriptional control.

## 4. Materials and methods

### 4.1 Growth and media

*Escherichia coli* strains for cloning were grown in LB-medium [61] at 37°C. If necessary, LB-medium was supplemented with 100 µg/ml Ampicillin.

For growth of *Haloferax volcanii* either non-selective YPC-medium or selective Ca-medium [62] was used. For cell shape analysis, CA-medium was supplemented with a trace element solution [29] to improve cell shape homogeneity. Cultures smaller than 5 ml were grown in 15 ml tubes while rotating. Larger cultures for microscopy were grown in 20 ml medium in 100 ml flasks under constant agitation at 120 rpm. Growth temperature for *H.* volcanii was 45°C. Plates were prepared as described before [62]. Motility plates were exactly prepared like described by Patro et al, [63].

For growth quantification of the csmR deletion strain cells were grown in 20 ml Cab medium and growth was continuously measured using the cell growth quantifier (CGQ, Software (CGQuant 7.4)) from Aquila Biolabs GmbH. Cell were grown at 45 °C in biological triplicates and H26 was used as control.

Archaeal and bacterial strains used in this study are listed (S1 Table).

### 4.2 Cloning

For plasmid construction all inserts were amplified using the PhusionHigh-Fidelity DNA polymerase obtained from New England Biolabs (NEB) following the manufactures protocol. Plasmids were either assembled via restriction enzyme-based cloning or in vivo ligation [64]. Enzymes for cloning were also obtained from NEB and the manufacturers protocol was followed. For in vivo ligation respective plasmids were linearized via polymerase chain reaction and subsequently cleaned by passing the linearized plasmids through an agarose gel. Approximately 50 ng linearized plasmid was mixed with app. 100 ng insert, the mixture transferred to 50 µl chemically competent *E. coli* and cells were then stored on ice for 30 min. Next, cells were heat shocked at 42°C for 1 min, 450 µl LB-medium was added and the cells incubated for 1.5 h at 37°C under constant shaking. Afterwards the whole cell suspension was plated on LB-plates supplemented with 100 µg/ml Ampicillin and the plates incubated overnight at 37°C. Colonies were transferred to 5 ml LB-medium supplemented with Ampicillin, grown over night and screened via colony PCR for successful plasmid generation the next day. Possible correct assembled plasmids were isolated using the Nucleospin Plasmid Easy Pure Kit (Macherey-Nagel) and send for sequencing. Used plasmids and primers are listed (S2 and S3 Tables).

### 4.3 Transformation in *Haloferax volcanii*

For the genetic modification of *Haloferax volcanii*, cells were transformed using polyethylene glycol [62]. Respective *H. volcanii* strains were grown in 10 ml YPC medium over-night at 45°C under constant rotation. The next day cells were harvested and resuspended in 2 ml buffered spheroplasting solution (1 M NaCl, 27 mM KCl, 50 mM Tris HCl pH 8.5, 15% sucrose (sterile filtered)), transferred to a round bottomed 2 ml reaction tube and centrifuged for 8 min at 3380 g. Supernatant was discarded and the pellet resuspended in 600 µl buffered spheroplasting solution. For one transformation 200 µl of the cell suspension was used. To generate spheroplasts 50 mM ethylenediaminetetraacetic acid (EDTA, pH 8) was added, the tube inverted and incubated for 10 min at room temperature. During incubation 1 µg of demethylated plasmid was mixed with 5 µl EDTA (0.5 M, pH 8) and then filled up to a total volume of 30 µl with unbuffered spheroplasting solution (1 M NaCl, 27 mM KCl, 15% sucrose (sterile filtered)). After 10 min the DNA mixture was added to the tube, mixed by inversion and incubated for 5 min. Subsequently 250 µl 60% polyethylene glycol 600 (diluted in unbuffered spheroplasting solution) was added to the cells and the tube was shaken horizontally for mixing. Thirty minutes later 1.5 ml spheroplast dilution solution (23% salt water, 15% sucrose, 3.75 mM CaCl_2_ (sterile filtered)) was added to the transformants, incubated for 2 min following centrifugation at 3380 g for 8 min. The supernatant was discarded and 1 ml regeneration solution (18% salt water, 1 x YPC-solution, 15% sucrose, 3.75 mM CaCl_2_ (sterile filtered)) added whilst the cell pellet was scratched off the wall. Transformants were then incubated at 45°C for 1.5 h without any agitation. Subsequently, cells were resuspended by tapping the tube and incubated for another 3 h at 45°C under constant rotation. The regenerated transformants were then harvested at 3380 g for 8 min, the supernatant discarded and the cell pellet resuspended in 1 ml transformant dilution solution (18% salt water, 15% sucrose, 3.75 mM CaCl_2_ (sterile filtered)). 100 µl of the resuspended cells were plated on selective Ca-plates and plates incubated in an airtight container at 45°C until colonies were visible.

### 4.4 Gene deletion in *Haloferax volcanii*

For the generation of deletion strains knock-out plasmids (S2 Table) of the genes to be knocked-out were transformed in the respective background strains as described above. Once colonies were visible after transformation, one colony per strain was transferred into 5 ml of non-selective YPC medium and grown over night at 45°C under constant rotation. The next day the growing culture was diluted back 1:500 into fresh 5 ml YPC-medium and grown over night. This process was repeated to a total of three times. Subsequently three dilutions of the growing culture, starting from 10^-2^, were plated on selective Ca-plates supplemented with 50 µg/ml 5-FOA and 10 µg/ml Uracil. Plates were incubated in an airtight container at 45°C until colonies were visible. To screen possible deletion strains 100 colonies per knock-out attempt were streaked on a YPC-plate and grown for two days at 45°C. Colony PCR was then used to screen for successful deletions using primers indicated in S3 Table.

### 4.5 Light microscopy

In order to examine the effect of the absence or overexpression of *csmR* on the cell shape of *Haloferax volcanii* cells, phase-contrast microscopy was used. Control strain H26 and the *csmR* deletion strain HTQ289 were either transformed with an “empty” expression plasmid (pTA1392) to complement the auxotrophic marker used in the genetic system or with the *csmR* overexpression plasmid pSVA13922. For microscopy, precultures were inoculated in 5ml Cab-medium and grown overnight at 45°C under constant rotation. The next day cells were diluted back into 20 ml fresh Cab-medium and grown to specific optical densities (OD_600_) under constant shaking at 45°C. When cells reached the desired ODs 5 µl cell culture was spotted on 1% agarose pads dissolved in 18% salt water. For cell shape analysis Fiji (Version 2.14.0) [65]plug-in MicrobeJ (Version 5.13l) [66]was used. Outliers in the cell shape datasets were identified by the ROUT method (Q = 1%) and removed. Cells of the *cirA, cirD, rosR,* regulatory *RNA* deletion strains as well as the double deletion strains of *csmR cirA* and *csmR cirD* were exactly prepared as the *csmR* single deletion strain.

Statistical comparison of cell shape parameters between the control and mutant populations were performed using Welch’s two-sample t-test which accounts for unequal variance and unequal sample sizes. Effect sizes were quantified using Cohen’s d.

Due to the large sample sizes employed in this study, Welch’s t-test yields extremely high statistical power, rendering virtually any difference between populations statistically significant even when the magnitude of that difference is negligible. Under such conditions, p-values alone are insufficient to assess the biological relevance of observed differences [67,68]. Accordingly, significance annotations have been omitted from cell shape plots to avoid overstating the biological importance of statistically significant but practically trivial differences. Instead, effect sizes (Cohen’s d) are reported alongside p-values in S4 Table to provide a comprehensive and transparent account of both, the statistical significance and the practical magnitude of each comparison. Effect sizes were interpreted according to conventional thresholds: d < 0.2 as negligible, 0.2 ≤ d ≤ 0.5 as small, 0.5 ≤ d ≤ 0.8 as medium and d ≥ 0.8 as large [69].

Summarized statistical analysis are shown in S4 Table.

### 4.6 Motility plates

To assess the effect of the different deletion strains on the ability of cell locomotion semi solid Ca-agar (0.3% w/v) plates were prepared as described above. Control strain H26 and the deletion strains were either transformed with pTA1392 or the indicated plasmids for overexpression of *csmR* or the *sRNA*. One colony of each transformed strain was inoculated in 5 ml Cab-medium and grown over night at 45°C under constant rotation. The next day the OD_600_ of the growing cultures was set to 0.2 by dilution with Ca-medium. Sterile toothpicks were used for inoculation of the motility plates. Plates that harbored strains containing the overexpression plasmid were supplemented with 12 mM Xylose, if not stated differently, to induce expression. Inoculated plates were airtight packed into plastic bags and incubated at 45°C for 3–4 days. Subsequently plates were scanned and the area of motility halos measured using Fiji. Statistical analysis was done using an unpaired Welch’s t-test.

### 4.7 Electron microscopy

Since the *csmR* deletion strain is non-motile electron microscopy was used to assess if archaella were present in the mutant strain. To ensure that the observed cell appendages on *Haloferax volcanii* were solely archaella a *pilB3* deletion strain as control and a *pilB3 csmR* double deletion strain was used. Both strains contained plasmid pTA1392 and were grown in 5 ml Ca-medium at 45°C, rotating. The next day the cultures were diluted back into 20 ml Ca-medium and grown to an optical density of 0.01 at 45°C under constant shaking. Cells were harvested and resuspended to a theoretical OD_600_ of 20 in Ca-medium. Next, 5 µl of cells were spotted on a glow discharged carbon coated copper grid (Plano GmbH, Wetzlar, Germany) and incubated for 10 sec. Excess liquid was removed using blotting paper and the cells were stained by addition of 2% uranyl acetate (w/v). Imaging was done with Hitachi HT7800 operated at 100 kV, equipped with an EMSIS Xarosa 20-megapixel CMOS camera. The effect of *cirA* and *cirD* deletion on archaellum formation was also assessed in a *pilB3* deletion background as described for the *csmR* deletion. Since the *cirA* deletion strain showed hypermotility, the *cirA pilB3* deletion strain was also imaged during stationary phase to see if archaella were still assembled. Each strain was imaged in biological triplicates.

### 4.8 Lipid extraction

Lipid were extracted from 3-14 mg freeze-dried *Haloferax volcanii* cells (variation caused by high salt levels) by applying an acidic Bligh and Dyer protocol, containing 5% trichloroacetic acid (TCA), as described elsewhere [70,71]. In short, freeze-dried cells were resuspended in 2:1:0.8 (v/v) MeOH:CHCl_3_:5% TCA, spiked with 10 µg of the internal standard n-Dodecyl β-maltoside (DDM), vortexed and sonicated for 60 min. Phase separation was obtained by addition of CHCl_3_ and 5% TCA. After centrifugation (2500 x g, 5 min at RT), the bottom phase was collected. To ensure total lipid extraction this procedure was repeated two times more. To remove TCA contaminations, the recovered bottom-phase was washed by adding 1:1:0.9 (v/v) MeOH:CHCl_3_:MQ. After centrifugation (2500 x g, 5 min at RT), the bottom phase was collected, dried under a nitrogen (N_2_) stream, and resuspended in 50 µl MeOH.

### 4.9 LC-MS analysis of lipids

Samples were analyzed using an Agilent Technologies 1290 Infinity high-performance liquid chromatography (HPLC) system, coupled to a heated electrospray ionization–mass spectrometer (Thermo Q Exactive Plus; Thermo Fisher Scientific). 5 µl was injected into an ACQUITY UPLC CSH C18 1.7 µm Column, 2.1 × 150 mm (Waters Chromatography Ireland Ltd) operating at 55 °C with a flow rate of 300 µl/min. Separation of the compounds was achieved by a changing gradient of eluent A (5 mM ammonium formate in water/acetonitrile 40:60, v/v) and eluent B (5 mM ammonium formate in acetonitrile/2-propanol, 10:90, v/v). The following linear gradient was applied: 1) 5% eluent B for 2.5 min, 2) a gradient from 5% to 90% eluent B over 36.5 min, 3) holding for 3 min, 4) returning to 5% eluent B in 0.5 min, 5) and holding for 8 min. The column effluent was injected directly into the Thermo Q Exactive Plus operating in positive and negative ion mode. Settings for positive mode: Spray Voltage: 3.20 |kV|, Capillary temperature: 230 °C, S-lens RF level: 50.0, sheath gas flow: 30, auxiliary gas flow: 5. Settings for negative mode: Spray Voltage: 3.20 |kV|, Capillary temperature: 230 °C, S-lens RF level: 50.0, sheath gas flow: 30, auxiliary gas flow: 20, sweep gas flow: 3, Aux gas heater temperature: 380 °C.

Spectral data constituting total ion counts were analyzed using the MacCoss Lab Software: Skyline. The following transition settings were used: Scan range: 133.4-2000 m/z, MS1 filtering: Orbitrap, resolving power 70,000 at m/z 400. MS/MS filtering: DDA, Orbitrap, resolving power: 17,500 at m/z 400. Total ion counts for extracted lipid species (S5 Table) were initially corrected for both the internal standard DDM (m/z 509.30 [M-H]− or m/z 528.34 [M + NH4]+) and the OD (due to high salt concentrations the freeze-dried mass could not be used). To further improve sample comparability, lipid species were normalized for total lipid ion count per sample and subsequently plotted on the y-axis.

### 4.10 RNA sequencing for differential gene expression analysis

#### 4.10.1 RNA extraction.

To obtain a comprehensive overview of the effect of the deletion of *csmR, cirA* and *cirD* on the transcriptome, RNA sequencing was used. RNA extraction, library preparation and sequencing has been done as described in [72] with minor modifications. Briefly, RNA was isolated from the deletion strains in lag- (OD_600_ 0.02), exponential- (OD_600_ 0.2), and stationary growth-phase (OD_600_ 2), H26 was used as a control. All strains were transformed with plasmid pTA1392 to avoid the addition of uracil to the selective CA-medium used for growth. To yield enough cells in lag-phase one-liter cultures were grown in 5 L flasks, cells for the exponential phase were grown in 20 ml cultures in 100 ml flasks and cells in stationary phase were grown in 3 ml cultures in 15 ml tubes, ensuring comparable aeration and oxygen availability across all growth phases. In total per strain and growth phase five replicates were grown. Once cells reached the wanted optical density cells were harvested and resuspended to a theoretical OD_600_ of 5. A maximum of two milliliters of resuspended cells were then transferred two a new reaction tube and pelleted. For RNA isolation the RNeasy Plus Mini Kit from QIAGEN was used. Pellets were resuspended in 600 µl RLT-plus buffer and RNA was isolated according to the manufacturers protocol. RNA was eluted in 31 µl RNAse free water (Roth) flash frozen in liquid nitrogen and stored at -80 °C.

#### 4.10.2 Library preparation and sequencing.

RNA quality was assessed using a Bioanalyzer, and only with an RNA integrity number (RIN) of 8.5 or higher were selected for further processing. Prior to library preparation, RNA was treated with Turbo DNase (Ambion, 1 unit) following the manufacturer’s protocol to eliminate any residual DNA. Four independent biological replicates were prepared for each experimental condition. To remove ribosomal RNA, 2 µg of input RNA was depleted using a ribopool specific to *H. volcanii* (siTOOLs) according to the manufacturer’s instructions.

Library preparation and RNA-sequencing followed the Illumina “Stranded mRNA Prep Ligation” Reference Guide, the Illumina NextSeq 2000 Sequencing System Guide (Illumina, Inc., San Diego, CA, USA), and the KAPA Library Quantification Kit - Illumina/ABI Prism (Roche Sequencing Solutions, Inc., Pleasanton, CA, USA). In brief, omitting the initial mRNA purification step with oligo(dT) magnetic beads, approximately 5 ng of rRNA depleted archaeal RNA was fragmented to an average insert size of 200–400 bases using divalent cations under elevated temperature (94°C for 8 minutes). Next, the cleaved RNA fragments were reverse transcribed into first strand complementary DNA (cDNA) using reverse transcriptase and random hexamer primers. Thereby Actinomycin D was added to allow RNA-dependent synthesis and to improve strand specificity by preventing spurious DNA-dependent synthesis. Blunt-ended second strand cDNA was synthesized using DNA Polymerase I, RNase H and dUTP nucleotides. The incorporation of dUTP, in place of dTTP, quenches the second strand during the later PCR amplification, because the polymerase does not incorporate past this nucleotide. The resulting cDNA fragments were adenylated at the 3’ ends and the pre-index anchors were ligated. Finally, DNA libraries were created using a 15 cycles PCR to selectively amplify the anchor-ligated DNA fragments and to add the unique dual indexing (i7 and I5) adapters. The bead purified libraries were quantified using the KAPA Library Quantification Kit. Equimolar amounts of each library were sequenced on an Illumina NextSeq 2000 instrument controlled by the NextSeq 2000 Control Software (NCS) v1. 5.0.42699, using one 50 cycles P3 Flow Cell with the dual index, single-read (SR) run parameters. Image analysis and base calling were done by the Real Time Analysis Software (RTA) v3.10.30. The resulting.cbcl files were converted into.fastq files with the bcl2fastq v2.20 software. Library preparation and RNA-sequencing were performed at the Genomics Core Facility “KFB - Center of Excellence for Fluorescent Bioanalytics” (University of Regensburg, Regensburg, Germany; www.kfb-regensburg.de).

#### 4.10.3 Differential gene expression analysis.

Raw sequencing reads in FASTQ format were processed for quality control and adapter trimming using fastp (v0.23.4) with the parameters --cut_front --cut_tail -q 30, ensuring the removal of low-quality bases. Filtered reads were then aligned to the *Haloferax volcanii* DS2 reference genome (GCF_000025685.1) using Bowtie2 (v2.5.3) with default settings. Sequence alignment files in SAM format were converted to BAM using SAMtools (v1.19.2) for downstream analysis.

Gene-level counts were obtained using feature Counts (part of the RSubread package v2.20.0) with a custom GTF file (gff filtered for entries labeled as gene). Differential expression analysis was performed using DESeq2 (v1.46.0) following Bioconductors guidelines. Principal component analysis (PCA) was applied to variance-stabilized data to assess overall data structure and identify outliers. Replicates identified as outliers (wild type low OD replicate 4, wild type mid OD replicate 1, ∆*csmR* mid OD replicate 4, ∆*cirA* mid OD replicate 3, ∆*cirA* stat OD replicate 1) were removed based on visual inspection. Comparative differential expression analysis was conducted between wild type and deletion strains across different growth conditions to identify growth-dependent transcriptional changes.

#### 4.10.4 Functional enrichment analysis based on arCOG classification.

To investigate the functional characteristics of differentially expressed genes, we performed a functional enrichment analysis using the Archaeal Clusters of Orthologous Genes (arCOG) classification, as previously described [72,73]. Briefly, arCOGs for *H. volcanii* were retrieved from [74], and gene set enrichment analysis was conducted using the goseq package (v1.58.0) in R. For each growth condition, a background gene set was generated from all detected genes. Overrepresentation *P*-values for arCOG categories were calculated separately for up- and downregulated genes based on RNA-seq-defined gene sets. Because this analysis was used to summarize broad functional trends, we report raw overrepresentation P-values and interpret these results as exploratory.

#### 4.10.5 Additional functional and structural analysis.

To explore functional relationships among differentially expressed genes, protein-protein interaction networks were analyzed using the STRING database [75].

Structural predictions were conducted using the AlphaFold Server with AlphaFold 3 model [76]. The predicted structure (CIF file) was downloaded and visualized in ChimeraX [77].

To identify structural homologs, Foldseek [78] was used, revealing 2P4W (https://doi.org/10.2210/pdb2P4W/pdb) as the closest match [79]. The AlphaFold-predicted structure and 2P4W were aligned using least-squares fitting in ChimeraX (match command).

Electrostatic surface potential was calculated and visualized using Coulombic Surface Coloring in ChimeraX.

To assess sequence conservation, a BLAST search was performed against the non-redundant (nr) database using HVO_1209 as the query sequence. Multiple sequence alignment (MSA) of all hits was generated using Clustal Omega [80] and visualized in ChimeraX, with sequences colored by conservation.

### 4.11 Chromatin immunoprecipitation sequencing (ChIP-seq)

#### 4.11.1 ChIP-seq sample preparation.

Chromatin immunoprecipitation was performed essentially as described previously with modifications for *Haloferax volcanii*. Cells expressing HA-tagged CsmR were grown to mid-exponential phase (OD₆₀₀ ≈ 0.2) in HEPES-buffered Ca-medium and harvested by centrifugation. Cells were resuspended in high-salt buffer (18% salt water) and crosslinked with 1% formaldehyde for 25 min at room temperature with agitation. Crosslinking was quenched by addition of glycine (final concentration 0.625 M), followed by multiple washes in ice-cold buffer. Cells were lysed using detergent (0.1% DDM) and chromatin was fragmented by sonication (50% amplitude, 30 s on/ 15 s off cycles, total 12 min) to obtain DNA fragments of approximately 200–500 bp. After removal of cell debris, the cleared lysate was used for immunoprecipitation. CsmR-DNA complexes were enriched using anti-HA magnetic beads (Pierce) and incubated for 1 h at room temperature with mixing. Beads were washed extensively and DNA–protein complexes were eluted. Crosslinks were reversed by incubation at 95°C, followed by RNase A and Proteinase K treatment. DNA was purified using a silica column-based cleanup kit (Zymo Research) and stored at −80°C.

#### 4.11.2 Library preparation and sequencing.

ChIP-seq libraries were prepared and sequenced at the Genomics Core Facility (KFB, University of Regensburg). Libraries were quantified using the KAPA Library Quantification Kit and pooled equimolarly. Sequencing was performed on an Illumina NextSeq 2000 platform using single-end 100 bp reads. Base calling was carried out using Illumina Real Time Analysis software, and FASTQ files were generated using bcl2fastq.

#### 4.11.3 Read processing and alignment.

Raw sequencing reads were quality-checked using FastQC (v0.12.1) and MultiQC (v1.33). Adapter trimming and quality filtering were performed using fastp (v1.3.0) with a minimum read length of 20 bp and a quality threshold of Q20. Reads were aligned to the *H. volcanii* reference genome (GCA_000025685.1) using Bowtie2 (v2.5.5) with the “--very-sensitive” preset. Alignments were filtered to retain primary mapped reads with a mapping quality ≥10. BAM files were sorted and indexed using SAMtools (v1.23.1). Normalized genome coverage tracks were generated using deepTools (v3.5.6, bamCoverage) with CPM normalization and a bin size of 1 bp.

#### 4.11.4 Peak calling.

Peaks were identified using MACS3 (v3.0.4) with matched input controls. Peak calling was performed for individual biological replicates as well as pooled samples using the following parameters: no model building (--nomodel), extension size of 250 bp, genome size of 4 Mb, and retention of all duplicate reads (--keep-dup all). A consensus peak set was generated by merging peaks from individual replicates using BEDTools (v2.31.1).

#### 4.11.5 ChIP-seq downstream analysis.

Peak files generated by MACS3 were imported into R (v4.5.1) and processed using GenomicRanges (v1.60.0) and tidyverse (v2.0.0) packages. High-confidence peaks were defined based on enrichment and significance thresholds (log10 q-value ≥ 5 and fold enrichment ≥ 2). Reproducibility across biological replicates was assessed by overlap analysis, and only peaks supported in all three replicates were retained. Redundant peaks were removed by selecting unique summit positions ranked by q-value and fold enrichment. For motif discovery, genomic sequences spanning ±100 bp around peak summits were extracted from the *H. volcanii* reference genome and used as input for MEME Suite (v5.5.9). Motif analysis was performed in zoops mode with reverse-complement search enabled and a genome-derived Markov background model. The predominant motif was used for downstream analyses. Motif occurrences were further evaluated using FIMO (v5.5.9), and similarity to known transcription factor binding sites was assessed using Tomtom. Peak-associated genes were assigned based on genomic proximity to annotated gene features imported from the reference GFF annotation. For each peak summit, overlapping genes were identified, or alternatively the nearest upstream and downstream genes were assigned. Distances between peak summits and gene start sites were calculated in a strand-aware manner to infer potential regulatory relationships. To integrate ChIP-seq and RNA-seq data, peaks were classified based on motif presence and orientation relative to the associated gene. Peaks with motif orientation matching the gene strand and located within a promoter-proximal window were defined as direct targets (Peak, matched orientation). Additional categories included peaks with motifs on the opposite strand (Peak, opposite orientation), peaks without detectable motifs (Peak, no motif), and operon-linked targets. Differences in gene expression between these groups were assessed using RNA-seq data, and statistical comparisons were performed using Wilcoxon rank-sum tests.

### 4.12 Northern blot analysis

Total RNA was prepared from *H. volcanii* strain as described for the transcriptome analysis. Cells were harvested at exponential phase (OD_650_ = 0.5) and at stationary phase (OD_650_ = 1.1-1.4). In addition, total RNA was isolated using the RNeasy Plus Kit (Quiagen) from the following strains: three biological replicates of H26 and ∆r*osR*, in each case at an OD_600_ of 0.02 and of 0.2. From all different RNA preparations, 10 µg each were separated on 1.5% agarose-gel, which were subsequently transferred to a nylon membrane (Biodyne A, PALL). After transfer, the membrane was hybridized with specific oligonucleotides (primer sequences are listed in S3 Table), which were radioactively labelled with [γ-^32^P]-ATP via polynucleotide kinase treatment to detect the *hvo_1211s*, *cirA* or *5S* transcript. The signals were recorded by an autoradiography film. For quantification of the *cirA* signal, an additional imaging plate (FujiFilm) was used for recording and the intensities were analysed using the ImageLab Software (Bio-Rad). The *cirA* signal was normalized with the *5S* signal, as a loading control.

## Supporting information

S1 Table Strains used in this study. (PDF)

S2 Table Plasmids used in this study. (PDF)

S3 Table Primers used in this study. (PDF)

S4 Table Statistics on cell shape analysis. Statistical comparison of cell length parameters between control and mutant populations. Results of Welch’s t-test and Cohen’s d effect size. Effect size interpretation: negligible (d < 0.2), small (0.2-0.5), medium (0.5-0.8), large (> 0.8).
(PDF)

S5 Table Detailed information of annotated lipids, including: lipid type, group, name, molecular formula, adduct type, theoretical m/z, and the mass error of the annotation. (PDF)

S6 Table RNA-seq library processing and mapping statistics. Summary of RNA-seq read processing statistics for all libraries included in the transcriptome analysis. The table reports raw read numbers, reads retained after quality trimming, reads mapped to the *Haloferax volcanii* reference genome, mapping efficiency, and sample metadata including strain, growth phase, and biological replicate.
(XLSX)

S7 Table Differential gene expression analysis of *H. volcanii* deletion strains across growth phases. DESeq2 differential expression results for pairwise comparisons of ∆*csmR*, ∆*cirA*, and ∆*cirD* strains against the wild-type H26 background at matched growth phases. Log2 fold changes indicate expression changes in the deletion strain relative to wild type. Statistical significance is reported as nominal p-values and Benjamini–Hochberg adjusted p-values.
(XLSX)

S8 Table arCOG enrichment analysis of differentially expressed genes. Functional enrichment analysis of upregulated and downregulated genes based on Archaeal Clusters of Orthologous Groups annotations. Enrichment was calculated separately for each strain and growth phase comparison, including category size, number of differentially expressed genes per category, expected gene counts, overrepresentation and underrepresentation p-values, and observed-to-expected deviations.
(XLSX)

S9 Table Normalized RNA-seq expression values across strains and growth phases. Size-factor-normalized RNA-seq count data used for comparative visualization of gene expression across wild type and deletion strains. Values are provided for matched growth phases and were used for downstream expression profiling of selected genes and regulatory groups.
(XLSX)

S10 Table CsmR motif occurrences detected in ChIP-seq peak-associated sequences. Motif occurrences identified from sequences centered on high-confidence CsmR ChIP-seq peak summits. The table reports the associated peak identifier, motif occurrence significance, motif sequence, inferred motif orientation, and position of the motif relative to the ChIP-seq peak summit.
(XLSX)

S11 Table High-confidence reproducible CsmR ChIP-seq peaks. High-confidence CsmR ChIP-seq peak set obtained from MACS3 peak calling and replicate-support filtering. Peaks were retained based on ChIP enrichment, statistical significance, and support across biological replicates. The table includes genomic coordinates, summit positions, peak width, pileup signal, fold enrichment, transformed p- and q-values, and replicate support.
(XLSX)

S12 Table Annotated CsmR ChIP-seq peaks and RNA-seq integration categories. Annotation of high-confidence CsmR ChIP-seq peaks with associated genomic features and RNA-seq integration groups. The table reports peak-level enrichment statistics, motif information, associated genes, strand-aware distances between peak summits and gene starts, genomic peak–gene relationships, and target categories used for integration of CsmR binding with differential gene expression.
(XLSX)

S1 Fig Phylogenetic distribution of *csmR* and its cooccurrence with archaellin genes amongst archaea. (A) The sunburst chart in depicts the taxonomic distribution of *csmR*-encoding species across the domain Archaea. Concentric rings represent finer taxonomic levels successively, moving outward from the domain level (center) through phylum, class, and order. Data were retrieved from the EggNOG database [81]. The angular size of each sector is proportional to the number of species within that taxon harboring a *csmR* ortholog. Colors distinguish major archaeal lineages: pink/red shading denotes Euryarchaeota (predominantly Halobacteria), and green shading denotes the TACK superphylum (predominantly Thaumarchaeota/Nitrosopumilaceae). Percentage values on the outermost ring indicate the fraction of all *csmR*-containing species assigned to each order. The predominance of red/pink sectors reflects that *csmR* is found almost exclusively in Halobacteria, with a secondary occurrence in Thaumarchaeota (Nitrosopumilaceae, 11%). (B) Shows a gene co-occurrence plot of *csmR* with the archaellin genes *arlA1* and *arlA2* based on the STRING database [82] (Version 12.0). The phylogenetic tree on the left (based on the STRING taxonomy) shows major archaeal lineages with Bacteria and Eukaryota as outgroups. Colored squares at internal tree nodes indicate taxonomic groupings according to colors in (A). The gene locus diagram (top right) illustrates the genomic neighborhood of *csmR* in *H. volcanii*: *csmR* (purple arrow), *hvo_1208* (yellow arrow), and *arlA1* and *arlA2* (red arrows). For each taxon row, the colored squares to the right of the tree show the predicted functional co-occurrence score (as computed by STRING) for each gene relative to *csmR*: dark red indicates a high confidence score, pale pink a lower confidence score, and an empty cell indicates no predicted co-occurrence. Squares split diagonally with a black triangle indicate that the gene is present in the STRING reference genome(s) for that taxon, with the colored portion still reflecting the co-occurrence score. The two gray columns on the far right demarcate the higher-level taxonomic groupings “Archaea” and “Euryarchaeota” within the STRING taxonomy and serve as a visual reference for the broader phylogenetic context. Together, the co-occurrence pattern shows that *csmR* most strongly co-occurs with *arlA1* and *arlA2* in Halobacteria, consistent with a predicted role in regulating archaellin-based motility.
(TIF)

S2 Fig AlphaFold3 structural prediction classifies CsmR as a Lrp/AsnC family regulator. AlphaFold3 structural prediction of a CsmR dimer, shown in two orientations. The confidence of the model is color-coded from high (blue) to low (yellow/red) based on the pLDDT score (per-residue measure of local confidence). The core structure is defined by a β-sandwich fold, which is highlighted and characteristic of the Lrp/AsnC family of transcriptional regulators. The per-residue confidence score is plotted below the structure.
(TIFF)

S3 Fig Phenotypic characterization of the *csmR* deletion strain compared to wildtype H26. (A) Growth curve of H26 and ΔcsmR over a period of 60 h. Each strain was grown in triplicate and the mean per triplicated is plotted together with its standard deviation. (B) Motility assay comparing wild type H26 with the Δ*csmR*. Exemplary motility halos of both strains are shown. The area of the motility halos was measured and normalized to the average area of wild type halos showing significantly (p ≤ 0.0001) decreased motility in the deletion strain compared to the H26 control. Samples were measured in biological and technical triplicates and all single data points per strain were plotted. The middle line indicates the mean and the upper and lower line the standard deviation. Scale bar 2 cm. (C) Cell shape analysis of the wild type H26 and the *csmR* deletion strain at low, mid and stationary OD_600_. Scale bar 4 µm. (D) Cell shape was analyzed using MicrobeJ and the summarized results of three independent biological replicates per strain and OD_600_ value plotted as violin-plots. The median is indicated by the middle black line, dotted lines indicate the first and third quartile. For each condition more than 1000 cells were analyzed. The dotted line indicates the length below which cells are considered plate shaped.
(TIF)

S4 Fig Uncropped transmission electron microscopy images. Images showing the whole cell TEM images of the different strains used. Arrows indicate the cell poles that were focused on in the main figures.
(TIF)

S5 Fig Complementation of the Δ*csmR* phenotype by expression of *csmR* from plasmid under different xylose concentrations as inducer. (A) 0 mM xylose: Motility assay comparing wildtype H26 with the *csmR* deletion strain (containing pTA1392) and both strains transformed with the *csmR* overexpression plasmid pSVA13922. An exemplary motility plate is shown. The area of the motility halos was measured and normalized to the average area of wildtype halos showing a significant reduction (p ≤ 0.0001) in cell locomotion of the *csmR* deletion strain compared to H26. Between H26, H26 + pSVA13922 and Δ*csmR* + pSVA13922 there is no difference in motility showing that the overexpression plasmid already complements the deletion phenotype back to wildtype levels even without inducer. (B) 4 mM xylose: Motility assay comparing the same strains as in (A). An exemplary motility plate is shown. Increased inducer concentration led to significantly increased (p ≤ 0.0001) motility of the strains transformed with the *csmR* expression plasmid compared to H26. (C) 8 mM xylose: Motility assay comparing the same strains as in (A). An exemplary motility plate is shown. Further increment of the inducer led to even higher motility of the pSVA13922 containing strains. All samples were measured in biological and technical triplicates and all single data points per strain were plotted. The middle line indicates the mean and the upper and lower line the standard deviation. Scale bar 2 cm.
(TIF)

S6 Fig Lipid species abundance throughout *H. volcanii* growth stages. Lipid species are presented as normalized ion counts. N = 4. Presented lipid species: phosphatidylglycerophosphate methyl ester (Me-PGP), phosphatidylglycerol (PG), phosphatidic acid (PA), phosphatidylethanolamine (PE), sulfo-digalactosyldiacylglycerol (S-DGD), digalactosyldiacylglycerol (DGD), monogalactosyldiacylglycerol (MGD), disulfo-digalactosyldiacylglycerol (2S-DGD), BPG (bi-phosphatidylglycerol)/aCL (archaeal cardiolipin), S-Gly-AHH (sulphated glycosylaminohexanehexaol [83,84]).
(TIF)

S7 Fig Lipid tail distribution of the four most abundant lipid species in *H. volcanii*, during different growth stages. (A) archaeol, (B) phosphatidylglycerophosphate methyl ester (Me-PGP), (C) sulfo-digalactosyldiacylglycerol (S-DGD) and (D) phosphatidylglycerol (PG). Lipid species are presented as normalized ion counts. N = 4.
(TIF)

S8 Fig Genome-wide distribution and representative profiles of CsmR ChIP-seq peaks. (A) Distribution of distances between CsmR ChIP-seq peak summits and annotated gene start sites. Negative values indicate upstream (promoter-proximal) localization. (B) Genome browser view showing CsmR ChIP-seq signal (three biological replicates) and corresponding input controls across a representative genomic region. High-confidence peaks are indicated by dashed lines, and associated downstream genes are marked. Gene annotations are shown below.
(TIF)

S9 Fig Growth-dependent expression profiles of genes associated with CsmR ChIP-seq peaks containing the consensus motif. Gene expression is shown as log₁₀(mean TPM + 1) across growth phases (early, mid, stationary) for wild-type (H26) and ∆csmR strains. Each panel represents an individual gene. Lines indicate mean expression across replicates.
(TIF)

S10 Fig Structure-guided PROMALS3D [85] alignment of CsmR with representative Lrp/AsnC family regulators. CsmR was aligned with Pyrococcus furiosus LrpA (1I1G), Escherichia coli AsnC (2 CG4), Bacillus subtilis LrpC (2CFX), and E. coli Lrp (2GQQ) using a structure-guided PROMALS3D alignment. The strongest correspondence is concentrated in the predicted N-terminal DNA-binding region of CsmR, whereas the central/C-terminal part is shorter and more divergent. In addition, the C-terminal extension present in canonical Lrp/AsnC regulators is absent in CsmR. Colored boxes indicate predicted secondary-structure elements in the alignment (orange, α-helices; light blue, β-strands).
(TIF)

S11 Fig Structural alignment of CsmR with the Phr regulator (PDB: 2P4W) using Foldseek. CsmR (purple) and Phr (gray) are shown in two orientations to highlight structural similarity. Despite low sequence identity (21.5%), structural alignment reveals a conserved core fold, particularly in the β-sandwich domain, suggesting a shared architectural framework. The E-values indicate statistically significant similarity.
(TIFF)

S12 Fig Electrostatic surface potential of CsmR. Electrostatic surface representation of CsmR shown in two orientations (top and bottom view). The color gradient represents the electrostatic potential, ranging from negatively charged regions (orange) to positively charged regions (purple).
(TIFF)

S13 Fig Evolutionary conservation of CsmR surface residues. Surface representation of CsmR colored by sequence conservation across halobacterial homologs, based on entropy-based conservation scores from AL2CO. Highly conserved residues are shown in dark purple, while variable regions are in white.
(TIFF)

S14 Fig DNA and protein interaction interface prediction for CsmR. DNA-protein and protein-protein interface prediction of the CsmR dimer retrieved from Alphafold3 prediction. Prediction confidence is shown by a color gradient from blue, no interface, to red for interface.
(TIF)

S15 Fig Comparative expression analysis of *cirA* and *cirD* genes across growth phases in WT and ∆*csmR* strains. (A) Mean transcripts per million (TPM) values of *cirA* and *cirD* across early (circle), mid (square), and stationary (diamond) growth phases in WT (grey) and ∆*csmR* (purple) strains. (B) Log_2_ fold-change in RNA expression (∆csmR vs. WT) for *cir* genes across growth phases. Significant genes are depicted as circles, and non-significant genes as squares. Color indicates specific *cir* genes (*cirA*: light blue, *cirD*: purple).
(TIFF)

S16 Fig Double deletions of *csmR cirD* shows similar phenotypes as the *csmR* single deletion mutant. (A). Cell shape analysis of the wild type H26 + pTA1392 and the *csmR cirD* + pTA1392 double deletion strain at low, mid and stationary OD_600_. Scale bar 4 µm. (B) Cell shape was analyzed using MicrobeJ and the summarized results of three independent biological replicates per strain and OD_600_ value plotted as violin-plots. The median is indicated by the middle black line, dotted lines indicate the first and third quartile. For each condition more than 1000 cells were analyzed. The dotted line indicates the length below which cells are considered plate shaped. (C) Motility assay of H26 + pTA1392 and the *csmR cirD* + pTA1392 double deletion strain. Exemplary motility halos of both strains are shown. The area of the motility halos was measured and normalized to the average area of wild type halos showing significantly (p ≤ 0.0001) decreased motility in the double deletion strain compared to the H26 control. Samples were measured in biological and technical triplicates and all single data points per strain were plotted. The middle line indicates the mean and the upper and lower line the standard deviation. Scale bar 2 cm.
(TIF)

S17 Fig Extensive overlap between CirA and CsmR regulatory networks highlights shared control of archaella-associated genes and motility pathways. (A), Morphological changes in wild type (H26), Δ*csmR*, Δ*cirA*, and Δ*cirD* strains during early, mid, and stationary growth phases. Wild type cells transition between motile rod-shaped and sessile plate-like morphologies, while Δ*csmR* and Δ*cirD* exhibit growth phase-dependent shape changes, and ΔcirA remains rod-shaped across all phases. (B), Principal component analysis (PCA) of transcriptomic data from wild type and deletion strains, showing variance explained by principal components 1 (PC1; 48%) and 2 (PC2; 20%). Biological replicates are color-coded by strain and growth phase (circles: early, squares: mid, diamonds: stationary). (C), Pairwise comparison of fold changes in WT vs. Δ*cirA* and WT vs. Δ*csmR*. Genes associated with CsmR ChIP-seq peaks are highlighted, with motif-containing peaks in matched orientation (dark green) and operon-linked genes (light green). Other genes are shown in grey. (D), UpSet plot showing the overlap of differentially regulated genes (padj < 0.1) across strains (∆*csmR*, ∆*cirA*) and growth phases (early, mid, stationary). Each row corresponds to a specific condition, and connected dots indicate gene sets shared between conditions. Bars represent the number of genes in each intersection. Colors indicate direction of regulation (up, down, or not significant). The analysis highlights a core set of genes consistently upregulated across all conditions. (E), STRING network analysis of overlapping genes, including archaella and chemotaxis components, revealing a highly interconnected regulatory network. High-confidence edges (confidence indicated by line width) indicate functional associations.
(TIF)

S18 Fig Gene regulation across strains and growth phases. Proportions of differentially expressed genes (upregulated, downregulated, or non-significant) in ∆*csmR*, ∆*cirA*, and ∆*cirD* strains compared to wild type across early, mid, and stationary growth phases. Bar segments indicate the percentage of genes in each category, with upregulated genes in green, downregulated genes in brown, and non-significant genes in beige.
(TIFF)

S19 Fig Deletion of *cir* genes reveals functional similarities between cirA and csmR in motility regulation, with distinct patterns in stationary phase. Gene set enrichment analysis of archaeal clusters of orthologous groups (arCOGs) across all deletion strains and growth phases. Circles indicate categories with raw overrepresentation P-value < 0.05, and color intensity reflects -log10(raw overrepresentation P-value). Separate analyses were performed for upregulated (padj < 0.05, log2FC >= 1) and downregulated (padj < 0.05, log2FC <= -1) genes across early, mid, and stationary growth conditions.
(TIFF)

S20 Fig Differential expression of potential csmR regulons across deletion strains. Log2 fold-change analysis (color-coded from brown to green) of genes in Δ*csmR*, Δ*cirA*, and Δ*cirD* strains across early, mid, and stationary phases. Significant upregulation (up triangle) and downregulation (down triangle) are shown. Genes associated with CsmR peaks are highlighted and grouped based on binding characteristics (motif presence, strand orientation, and promoter distance.
(TIF)

S21 Fig Correlation analysis of differential gene expression between deletion strains highlights distinct and shared effects on gene regulation. (A) Scatter plot comparing log2 fold changes (WT vs. *ΔcsmR* and WT vs. *ΔcirD*) during the stationary phase and (B) WT vs. Δ*csmR* and WT vs. Δ*rosR* during the mid-phase. Pearsons’s correlation was calculated based on pairwise complete observations. Genes associated with CsmR ChIP-seq peaks are highlighted, with motif-containing peaks in matched orientation (dark green) and operon-linked genes (light green). Other genes are shown in grey. (C) Heatmap of correlation coefficients for log2 fold changes across strains (Δ*csmR*, Δ*cirA*, Δ*cirD*, Δ*rosR*) and growth phases (early, mid, stationary).
(TIF)

S22 Fig Deletion of *rosR* impacts motility and cell shape. (A) Motility assay comparing wild type H26 + pTA1392 with the *rosR* + pTA1392 deletion strain. Exemplary motility halos of both strains are shown. Scale bar 2 cm. (B) The area of the motility halos was measured and normalized to the average area of wild type halos showing significantly (p ≤ 0.0001) decreased motility of the *rosR* deletion strain compared to H26. Samples were measured in biological and technical triplicates and all single data points per strain were plotted. The middle line indicates the mean and the upper and lower line the standard deviation. (C) Exemplary images of the wild type H26 + pTA1392 and the *rosR* + pTA1392 deletion strain at low, mid and stationary OD_600_. Deletion of *rosR* inhibits rod formation. Scale bar 4 µm. (D) Cell shape was analyzed using MicrobeJ and the summarized results of three independent biological replicates per strain and OD_600_ value plotted as violin-plots. The median is indicated by the middle black line, dotted lines indicate the first and third quartile. The dotted line indicates the length below which cells are considered plate shaped. (E) Cell circularity was analyzed using MicrobeJ and the summarized results of three independent biological replicates per strain and OD_600_ value plotted as violin-plots. The median is indicated by the middle black line, dotted lines indicate the first and third quartile. For each condition more than 1000 cells were analyzed.
(TIF)

S23 Fig Northern blot analysis. (A) Northern blot analysis using an anti-*cirA* probe on RNA extracted from the wild type H26 and the *rosR* deletion strain at low (OD_600_ 0.02) and mid (OD_600_ 0.2) growth. Anti *5S* probe was used as loading control. (B) Quantification of the signal by the *cirA* probe normalized to the *5S* signal. Plotted are the results from at least three independent experiments per strain and growth phase. (C) Detection of the *hvo_1211s* transcript using RNA extracted from H26 on a Northern blot with a size of approximately 400 nucleotides.
(TIF)

S24 Fig Schematic overview of the genetic region between *csmR* (*hvo_1209*) and *cirA* (*hvo_1212*). RosR binding to the 3’ end of *arlA2* is indicated by a yellow box. The removed part of the partial *cirA* deletion is indicated in dark blue.
(TIF)

S25 Fig A partial deletion of *cirA* leaving the *hvo_1211s* intact shows the same phenotype as Δ*cirA.* (A) Motility assay comparing wild type H26 + pTA1392 with the *cirA* + pTA1392 deletion strain and the partial *cirA* deletion strain. An exemplary motility plate is shown. (B) The area of the motility halos was measured and normalized to the average area of wild type halos showing significantly (p ≤ 0.0001) increased motility in the *cirA* and the partial *cirA* deletion strain deletion strain compared to the H26 control. Samples were measured in biological and technical triplicates and all single data points per strain were plotted. The middle line indicates the mean and the upper and lower line the standard deviation. (C) Cell shape analysis of the wild type H26 + pTA1392 and the partial *cirA* + pTA1392 deletion strain at low, mid and stationary OD_600_. The partial *cirA* deletion strain showed the same phenotype as the full Δ*cirA* strain. Cells stayed rod-shaped during the full growth cycle. Scale bar 4 µm. (D) Cell shape was analyzed using MicrobeJ and the summarized results of three independent biological replicates per strain and OD_600_ value plotted as violin-plots. The median is indicated by the middle black line, dotted lines indicate the first and third quartile. For each condition more than 1000 cells were analyzed. The dotted line indicates the length below which cells are considered plate shaped.
(TIF)
