# Supplementary material for: CsmR controls both, motility and cell shape, in Haloferax volcanii
Source: PLoS Genet. 2026 Jun 12;22(6):e1012198. doi: 10.1371/journal.pgen.1012198 (PMC13286277; doi:10.1371/journal.pgen.1012198)
Supplement: S1 Table — (PDF) [file pgen.1012198.s001.pdf]

S1 Table: Strains used in this study

Strain Name	Background strain	Genotype	Source/reference
Haloferax volcanii			
H26	-	Δ*pyrE2*	[1]
H119	DS70	ΔpyrE2, ΔtrpA, ΔleuB	[2]
HTQ277	H26	Δ*pyrE2* Δ*cirA*	This study
HTQ289	H26	Δ*pyrE2* Δ*csmR*	This study
HTQ247	H26	Δ*pyrE2* Δ*pilB3*	[3]
HTQ292	HTQ247	Δ*pyrE2* Δ*pilB3* Δ*csmR*	This study
HTQ293	HTQ289	Δ*pyrE2* Δ*csmR* Δ*cirA*	This study
HTQ296	H26	Δ*pyrE2* Δ*cirD*	This study
HTQ817	HTQ277	Δ*pyrE2* Δ*pilB3* Δ*cirA*	This study
HTQ820	HTQ296	Δ*pyrE2* Δ*pilB3* Δ*cirD*	This study
HTQ1005	HTQ289	Δ*pyrE2* Δ*csmR* Δ*cirD*	This study
HTQ968	H26	Δ*pyrE2* partial Δ*hvo_1211s*	This study
HTQ1009	H26	Δ*pyrE2* partial Δ*cirA*	This study
HTQ966	H26	Δ*pyrE2* Δ*rosR*	This study
Escherichia coli			
NEB® 5-alpha	-	*fhuA2Δ(argF-lacZ)U169 phoA glnV44 Φ80Δ(lacZ)M15 gyrA96 recA1 relA1 endA1 thi-1 hsdR17*	New England Biolabs
dam^{-}/*dcm*$^{-}$ Competent Cells	-	*ara-14 leuB6 fhuA31 lacY1 tsx78 glnV44 galK2 galT22 mcrA dcm-6 hisG4 rfbD1 R(zgb210::Tn10)* Tet^{S} *endA1 rspL136* (Str^{R}) *dam13::Tn9* (Cam^{R}) *xylA-5 mtl-1 thi-1 mcrB1 hsdR2*	New England Biolabs
