# Supplementary material for: CsmR controls both, motility and cell shape, in Haloferax volcanii
Source: PLoS Genet. 2026 Jun 12;22(6):e1012198. doi: 10.1371/journal.pgen.1012198 (PMC13286277; doi:10.1371/journal.pgen.1012198)
Supplement: S2 Table — (PDF) [file pgen.1012198.s002.pdf]

**S2 Table:** Plasmids used in this study

| Plasmids | Description | Primers used | Enzymes used | Source/reference |
|---|---|---|---|---|
| pTA131 | Integrative plasmid with a *pyrE2* selection marker for gene deletions in *H. volcanii* (Amp$^r$) | - | - | [2] |
| pTA1392 | Plasmid for the expression of proteins in *H. volcanii* under control of *p.tnaA* and *pyrE2, hdrB* selection markers. Used to complement uracil auxotrophy (Amp$^r$) | - | - | [4] |
| pSVA5681 | Integrative plasmid for the generation of a *cirA* deletion strain (Amp$^r$) | 11335, 11336; 11337, 11338 | KpnI, XbaI | This study |
| pSVA13742 | Integrative plasmid for the generation of a *csmR* deletion strain (Amp$^r$) | 13704, 13705; 13706, 13707 | in vivo ligation | This study |
| pSVA6082 | Plasmid for the expression of proteins in *H. volcanii* under control of *p.xyl* and *pyrE2, hdrB* selection markers (Amp$^r$) | 13531, 13532 | ApaI, NdeI | This study |
| pSVA13922 | Plasmid for the expression of *csmR* under the control of a xylose promotor (Amp$^r$) | 13828, 14612 | PciI, BamHI (plasmid); NcoI, BamHI (insert) | This study |
| pSVA13767 | Integrative plasmid for the generation of a *cirD* deletion strain (Amp$^r$) | 13780, 13781; 13782, 13783 | in vivo ligation | This study |
| pSVA3999 | Integrative plasmid for the generation of a *pilB3* deletion strain (Amp$^r$) | - | - | [3] |
| pSVA13966 | Plasmid for the expression of *csmR-ha* under the control of a xylose promotor (Amp$^r$) | 15326, 15327 | NdeI, BamHI | This study |

| | | | | |
|---|---|---|---|---|
| pSVA13982 | Integrative plasmid for the generation of a partial *hvo_1211s* deletion strain (Amp$^r$) | 15334, 15335; 15336, 15337 | in vivo ligation | This study |
| pSVA13979 | Integrative plasmid for the generation of a partial *cirA* deletion strain (Amp$^r$) | 15359, 15360; 15361, 15362 | in vivo ligation | This study |
| pSVA13964 | Integrative plasmid for the generation of a *rosR* deletion strain (Amp$^r$) | 15320, 15321; 15322, 15323 | in vivo ligation | This study |
| pSVA13977 | Plasmid for the expression of *hvo_1211s* under the control of a xylose promotor (Amp$^r$) | 15340, 15363 | NdeI, BamHI | This study |
