# Supplementary material for: CsmR controls both, motility and cell shape, in Haloferax volcanii
Source: PLoS Genet. 2026 Jun 12;22(6):e1012198. doi: 10.1371/journal.pgen.1012198 (PMC13286277; doi:10.1371/journal.pgen.1012198)
Supplement: S3 Table — (PDF) [file pgen.1012198.s003.pdf]

S3 Table: Primers used in this study

Primer Number/Name	Sequence 5'→3'	Description
11066	TCTAGAGCGGCCGCCAC	Forward primer for the linearization of pTA131
11067	GGTACCCAATTCGCCCTATAGTG	Reverse primer for the linearization of pTA131
11335	CGGGGTACCTCTGCGAGTTCCTCGCTGTTGTCGTCAC	Forward primer for the amplification of the up-stream region of *hvo_1212* (*cirA*) with KpnI restriction site
11336	GGTTGTGTTGAAACGGGTGTTGGCTGTCGG	Reverse primer for the amplification of the up-stream region of *hvo_1212* (*cirA*)
11337	ACCCGTTTCAACACAACCCGGGTCGGTCCTGAAGGCCGGTGGCCCCGATC	Forward primer for the amplification of the down-stream region of *hvo_1212* (*cirA*) with 15 bp overhang to the *cirA* upstream fragment
11338	TGCTCTAGATCGGCGTCCTCAACAGCATGAACGACC	Reverse primer for the amplification of the down-stream region of *hvo_1212* (*cirA*) with XbaI restriction site
11339	GGGTCGAGATACTCTTCGTCCTCGGCTTCC	Forward primer for screening and sequencing of *cirA* deletion and the partial *cirA* deletion strain
11340	GCGGTGGACATGAACGAGACCTCGATTAAG	Reverse primer for screening and sequencing of *cirA* deletion and the partial *cirA* deletion strain
13531	TTCGGGCCCCCCGTCTTTCGTCTCCCGTCTCC	Forward primer for the amplification of a 1 kb up-stream fragment of *hvo_B0025* with an ApaI restriction site
13532	GAATTCGCATATGCAGTATCCTCATTACCAGCG	Reverse primer for the amplification of a 1 kb up-stream fragment of *hvo_B0025* with an NdeI restriction site
13704	GGCGAATTGGGTACCGAGCGCGGTGGTGAC	Forward primer for the amplification of the up-stream region of *hvo_1209* (*csmR*) with 15 complementary bases to linearized pTA131
13705	CGGAGTCGGAGCGGTAGATGGAGATTTATCAGGACG	Reverse primer for the amplification of the up-stream region of *hvo_1209* (*csmR*) with 15 bp overhang to the *csmR* downstream fragment
13706	GATAAATCTCCATCTACCGCTCCGACTCCG	Forward primer for the amplification of the down-stream region of *hvo_1209* (*csmR*) with 15 bp overhang to the *csmR* upstream fragment

13707	GGCGGCCGCTCTAGAAGGTACTTGACCGTCGTATCTTC	Reverse primer for the amplification of the down-stream region of *hvo_1209* (*csmR*) with 15 complementary bases to linearized pTA131
13708	ATAGGTAACCCGCACCTCCG	Forward primer for screening and sequencing of *csmR* deletion strain
13709	ACGGTGTCGTTGTAGGTGAG	Reverse primer for screening and sequencing of *csmR* deletion strain
13828	CATGCCATGGAGTCTGACAGGACCCTCTC	Forward primer for the amplification of *csmR* with NcoI restriction site (cloned in pSVA6082)
14612	CTAGCTAGCTCAGTCCTGCGTAATCTTCC	Reverse primer for the amplification of *csmR* with BamHI restriction site (cloned in pSVA6082)
13780	GGCGAATTGGGTACCTTGCGGCCTTCGACGAACTC	Forward primer for the amplification of the up-stream region of *hvo_2232* (*cirD*) with 15 complementary bases to linearized pTA131
13781	GGTCGGACACTCGCCCGCC	Reverse primer for the amplification of the up-stream region of *hvo_2232* (*cirD*)
13782	CGGGCGAGTGTCCGACCGGTAACAACAATGTACGAAC	Forward primer for the amplification of the down-stream region of *hvo_2232* (*cirD*) with 15 bp overhang to the *cirD* upstream fragment
13783	GGCGGCCGCTCTAGATGTCGCTCATGCGTCGAGAG	Reverse primer for the amplification of the down-stream region of *hvo_2232* (*cirD*) with 15 complementary bases to linearized pTA131
13784	CCATCGACACTTACGACCCC	Forward primer for screening and sequencing of *cirD* deletion strain
13785	ATAGGCGTCCTCCGAGAGTC	Reverse primer for screening and sequencing of *cirD* deletion strain
8893	GCCGACGAGAGCGACCTGAC	Forward primer for screening and sequencing of *pilB3* deletion strain
8894	CGCGTCGCCATCGTCTGGAG	Reverse primer for screening and sequencing of *pilB3* deletion strain
15320	GGCGAATTGGGTACCTGCGGGATGAAGCCGTAG	Forward primer for the amplification of the up-stream region of *hvo_0730* (*rosR*) with 15 complementary bases to linearized pTA131
15321	GCATATGGAAATGTCATCTGCCTATTTAAACTTTGTC	Reverse primer for the amplification of the up-stream region of *hvo_0730* (*rosR*)
15322	GACATTTCCATATGCTCGGCTGCGTCCACCGCAC	Forward primer for the amplification of the down-stream region of

		hvo_0730 (*rosR*) with 15 bp overhang to the *rosR* upstream fragment
15323	GGCGGCCGCTCTAGATTTCCGAA GCCGAGCGACTG	Reverse primer for the amplification of the down-stream region of *hvo_0730* (*rosR*) with 15 complementary bases to linearized pTA131
15324	TCGCGAAGAACTCGTCAATC	Forward primer for screening and sequencing of *hvo_0730* (*rosR*) deletion strain
15325	TCAAAGAGCGCCGCGTGAAG	Reverse primer for screening and sequencing of *hvo_0730* (*rosR*) deletion strain
15326	GGAATTCCATATGGAGTCTGACA GGACCCTCTC	Forward primer for the amplification of *csmR* with NdeI restriction site (cloned in pSVA6082)
15327	GCGGGATCCTCACGCGTAGTCCG GGACGTCGTACGGGTAGCCGCCG CCGCCGTCCTGCGTAATCTTCCGC C	Reverse primer for the amplification of *csmR* with BamHI restriction site and the HA-taq sequence (cloned in pSVA6082)
15334	GGCGAATTGGGTACCAGAGCAGT CCGTGAACAAGG	Forward primer for the amplification of the up-stream region of *hvo_1211s* with 15 complementary bases to linearized pTA131
15335	GCCGCTCGGCAGTCGACGTCG	Reverse primer for the amplification of the up-stream region of *hvo_1211s*
15336	CGACTGCCGAGCGGCAAAACAG GGAGAGCGCAGCG	Forward primer for the amplification of the down-stream region of the intergenic region between *arlA2* and *cirA* with 15 bp overhang to the *hvo_1211s* upstream fragment
15378	AATGAAAGGAAAGTTTCGGTG	Reverse primer for the amplification of the down-stream region of *hvo_1211s* with 15 complementary bases to linearized pTA131
15379	AACTTTCCTTTCATTAAAACAGGG AGAGCGCAGCG	Forward primer for screening and sequencing of the partial *hvo_1211s* deletion strain
15339	TGGCACGAGTACGTCGAGTC	Reverse primer for screening and sequencing of the partial *hvo_1211s* deletion strain
15340	GGAATTCCATATGCAGCGCTGTC GTTTGTTCGG	Forward primer for the amplification of *hvo_1211s* with NdeI restriction site (cloned in pSVA6082)
15363	CGCGGATCCGACCGACATCGGCC TCGAG	Reverse primer for the amplification of *hvo_1211s* with BamHI restriction site (cloned in pSVA6082)

15359	GGCGAATTGGGTACCGCTGTCGTTTGTTCGGAGTG	Forward primer for the amplification of the up-stream region of *hvo_1212* (*cirA*) with 15 complementary bases to linearized pTA131
15360	CCGAGACCGACATCGGCCTCGAG	Reverse primer for the amplification of the up-stream region of *hvo_1212* (*cirA*)
15361	CGATGTCGGTCTCGGGGTTGTGTTGAAACGGGTG	Forward primer for the amplification of the *hvo_1212* (*cirA*) region where *hvo_1211s* is coded on with 15 bp overhang to the *cirA* upstream fragment
15362	GGCGGCCGCTCTAGACACCGAATCCGCCGAATTCG	Reverse primer for the amplification of the down-stream region of the *hvo_1212* (*cirA*) region where *hvo_1211s* is coded on with 15 complementary bases to linearized pTA131
Anti- *cirA*	CGTCAGCGAGTCGAGGACGAGCCGGTCGTAGTTGG	Probe against *cirA* for Northern blot analysis
Anti - 5S	CGCAGGTGAGCTTAACTTCCGTGTTCGGG	Probe against *5S-rRNA* for Northern blot analysis
Anti - *hvo_1211s*	CCAGCTACTGTCTCCTCGCAGTTCGTCTCGGCTCACCGTCC	Probe against *hvo_1211s* for Northern blot analysis
