# Supplementary material for: CsmR controls both, motility and cell shape, in Haloferax volcanii
Source: PLoS Genet. 2026 Jun 12;22(6):e1012198. doi: 10.1371/journal.pgen.1012198 (PMC13286277; doi:10.1371/journal.pgen.1012198)
Supplement: S4 Table — Statistical comparison of cell length parameters between control and mutant populations. Results of Welch’s t-test and Cohen’s d effect size. Effect size interpretation: negligible (d < 0.2), small (0.2-0.5), medium (0.5-0.8), large (> 0.8). (PDF) [file pgen.1012198.s004.pdf]

S4 Table: Statistics on cell shape analysis

Statistical comparison of cell length parameters between control and mutant populations. Results of Welch's t-test and Cohen's d effect size. Effect size interpretation: negligible (d < 0.2), small (0.2-0.5), medium (0.5-0.8), large (> 0.8).

			**p-value**	**Significance**	**Effect sizes Cohen's |d|**
**cell length Δ*csmR***					
		H26 vs Δ*csmR* OD 0.02	<0.0001	****	-0,564
		H26 vs Δ*csmR* OD 0.2	<0.0001	****	-0,234
		H26 vs Δ*csmR* OD 2	0.1001	-	-0,031
**cell length *csmR* overexpression**					
		H26 vs H26 *csmR* overexpression OD 0.02	<0.0001	****	-0,173
		H26 vs H26 *csmR* overexpression OD 0.2	<0.0001	****	-0,432
		H26 vs H26 *csmR* overexpression OD 2	<0.0001	****	-2,441
		H26 *csmR* overexpression vs Δ*csmR* *csmR* overexpression OD 0.02	<0.0001	****	-0,288
		H26 *csmR* overexpression vs Δ*csmR* *csmR* overexpression overexpression OD 0.2	<0.0001	****	-0,292
		H26 *csmR* overexpression vs Δ*csmR* *csmR* overexpression overexpression OD 2	0.36	-	0,035
**cell length Δ*cirA***					
		H26 vs Δ*cirA* OD 0.02	<0.0001	****	-0,645
		H26 vs Δ*cirA* OD 0.2	<0.0001	****	-0,875
		H26 vs Δ*cirA* OD 2	<0.0001	****	-1,201
**cell length Δ*cirD***					
		H26 vs Δ*cirD* OD 0.02	0.0056	**	-0,171
		H26 vs Δ*cirD* OD 0.2	0.0038	**	0,118
		H26 vs Δ*cirD* OD 2	<0.0001	****	-0,118
**cell length Δ*csmR* Δ*cirA***					

		H26 vs Δ*csmR* Δ*cirA* OD 0.02	<0.0001	****	-0,633
		H26 vs Δ*csmR* Δ*cirA* OD 0.2	<0.0001	****	0,157
		H26 vs Δ*csmR* Δ*cirA* OD 2	<0.0001	****	0,721
**cell length Δ*csmR* Δ*cirD***					
		H26 vs Δ*csmR* Δ*cirD* OD 0.02	<0.0001	****	0,558
		H26 vs Δ*csmR* Δ*cirD* OD 0.2	0.1601	-	0,043
		H26 vs Δ*csmR* Δ*cirD* OD 2	<0.0001	****	0,625
**cell length Δ*rosR***					
		H26 vs Δ*rosR* OD 0.02	<0.0001	****	0,616
		H26 vs Δ*rosR* OD 0.2	0.9141	-	-0,004
		H26 vs Δ*rosR* OD 2	0.4045	-	0,013
**cell circularity Δ*rosR***					
		H26 vs Δ*rosR* OD 0.02	<0.0001	****	-1,357
		H26 vs Δ*rosR* OD 0.2	<0.0001	****	-0,489
		H26 vs Δ*rosR* OD 2	0.0026	**	0,044
**cell length Δ*hvo_1211s***					
		H26 vs Δ*hvo_1211s* OD 0.02	<0.0001	****	0,259
		H26 vs Δ*hvo_1211s* OD 0.2	0.5957	-	0,013
		H26 vs Δ*hvo_1211s* OD 2	0.0428	*	0,035
**cell length *hvo_1211s* overexpression**					
		H26 vs H26 *hvo_1211s* overexpression OD 0.02	<0.0001	****	0,488
		H26 vs H26 *hvo_1211s* overexpression OD 0.2	<0.0001	****	0,148
		H26 vs H26 *hvo_1211s* overexpression OD 2	<0.0001	****	1,094
**cell length partial Δ*cirA***					
		H26 vs partial ΔcirA OD 0.02	<0.0001	****	-0,495

		H26 vs partial cirA ko OD 0.2	<0.0001	****	-0,727
		H26 vs partial cirA ko OD 2	<0.0001	****	-1,728
**cell length Δ*csmR* without plasmid**					
		H26 vs csmR ko OD 0.02	<0.0001	****	-0,959
		H26 vs csmR ko OD 0.2	<0.0001	****	-0,162
		H26 vs csmR ko OD 2	<0.0001	****	-0,067
