# Supplementary material for: CsmR controls both, motility and cell shape, in Haloferax volcanii
Source: PLoS Genet. 2026 Jun 12;22(6):e1012198. doi: 10.1371/journal.pgen.1012198 (PMC13286277; doi:10.1371/journal.pgen.1012198)
Supplement: S5 Table — (PDF) [file pgen.1012198.s005.pdf]

S5 Table: Detailed information of annotated lipids, including: lipid type, group, name, molecular formula, adduct type, theoretical m/z, and the mass error of the annotation.

Lipid type	Lipid group	Lipid name	Formula	Adduct type	Theoretical m/z	Mass error (ppm)
Phospholipids	PA	PA (40:0)	C43H89O6P	[M-H]-	731.6324	1.1
Phospholipids	PE	PE (40:0)	C45H94O6NP	[M-H]-	774.6746	1.1
Phospholipids	Me-PGP	Me-PGP (40:0)	C47H98O11P2	[M-H]-	899.6512	0.8
Phospholipids	Me-PGP	Me-PGP (40:1)	C47H96O11P2	[M-H]-	897.6355	1.0
Phospholipids	Me-PGP	Me-PGP (40:2)	C47H94O11P2	[M-H]-	895.6199	1.1
Phospholipids	Me-PGP	Me-PGP (40:3)	C47H92O11P2	[M-H]-	893.6042	0.8
Phospholipids	Me-PGP	Me-PGP (40:4)	C47H90O11P2	[M-H]-	891.5886	1.2
Phospholipids	Me-PGP	Me-PGP (45:0)	C52H108O11P2	[M-H]-	969.7294	0.9
Phospholipids	Me-PGP	Me-PGP (45:2)	C52H104O11P2	[M-H]-	965.6981	1.0
Phospholipids	PG	PG (40)	C46H95O8P	[M-H]-	805.6692	0.8
Phospholipids	PG	PG (40:1)	C46H93O8P	[M-H]-	803.6535	0.9
Phospholipids	PG	PG (40:2)	C46H91O8P	[M-H]-	801.6379	1.1
Phospholipids	PG	PG (40:3)	C46H89O8P	[M-H]-	799.6222	0.7
Phospholipids	PG	PG (40:4)	C46H87O8P	[M-H]-	797.6066	1.0
Phospholipids	PG	PG (45:0)	C51H105O8P	[M-H]-	875.7474	0.9
Phospholipids	PG	PG (45:2)	C51H101O8P	[M-H]-	871.7161	1.1
Cardiolipin	BPG	BPG (80:0)	C89H182O13P2	[M-H]-	1520.2983	1.0
Cardiolipin	DGD-PA	DGD-PA (80:0)	C98H195O18P	[M-H]-	1690.4008	0.9
Cardiolipin	S-DGD-PA	S-DGD-PA (80:0)	C98H195O21PS	[M-H]-	1770.3576	0.7
Glycolipids	MGD	MGD (40:0)	C49H98O8	[M+HCOO]-	859.7244	1.1
Glycolipids	DGD	DGD (40:0)	C55H108O13	[M+HCOO]-	1021.7772	1.1
Glycolipids	S-DGD	S-DGD (40:0)	C55H108O16S	[M-H]-	1055.7285	0.3
Glycolipids	S-DGD	S-DGD (40:1)	C55H106O16S	[M-H]-	1053.7129	1.8
Glycolipids	S-DGD	S-DGD (40:2)	C55H104O16S	[M-H]-	1051.6972	0.7
Glycolipids	S-DGD	S-DGD (40:3)	C55H102O16S	[M-H]-	1049.6816	1.7
Glycolipids	S-DGD	S-DGD (40:4)	C55H100O16S	[M-H]-	1047.6659	0.8
Glycolipids	S-DGD	S-DGD (45:0)	C60H118O16S	[M-H]-	1125.8068	0.5
Glycolipids	2S-DGD	2S-DGD (40:0)	C55H108O19S2	[M-H]-	1135.6853	0.6
Glycolipids	S-Gly-AHH	S-Gly-AHH (40:0)	C55H111O16NS	[M+NH4]+	1091.7962	0.8
Archaeol	Archaeol	Archaeol (40:0)	C43H88O3	[M+NH4]+	670.7072	0.9
Archaeol	Archaeol	Archaeol (40:1)	C43H86O3	[M+NH4]+	668.6915	1.1
Archaeol	Archaeol	Archaeol (40:2)	C43H84O3	[M+NH4]+	666.6759	0.6
Archaeol	Archaeol	Archaeol (40:3	C43H82O3	[M+NH4]+	664.6602	0.9
Archaeol	Archaeol	Archaeol (40:4)	C43H80O3	[M+NH4]+	662.6446	0.7
Archaeol	Archaeol	Archaeol (45:0)	C48H98O3	[M+NH4]+	740.7854	1.2
Archaeol	Archaeol	Archaeol (45:2)	C48H94O3	[M+NH4]+	736.7541	0.5
