# Supplementary figures and images for: CsmR controls both, motility and cell shape, in Haloferax volcanii
Source: PLoS Genet. 2026 Jun 12;22(6):e1012198. doi: 10.1371/journal.pgen.1012198 (PMC13286277; doi:10.1371/journal.pgen.1012198)

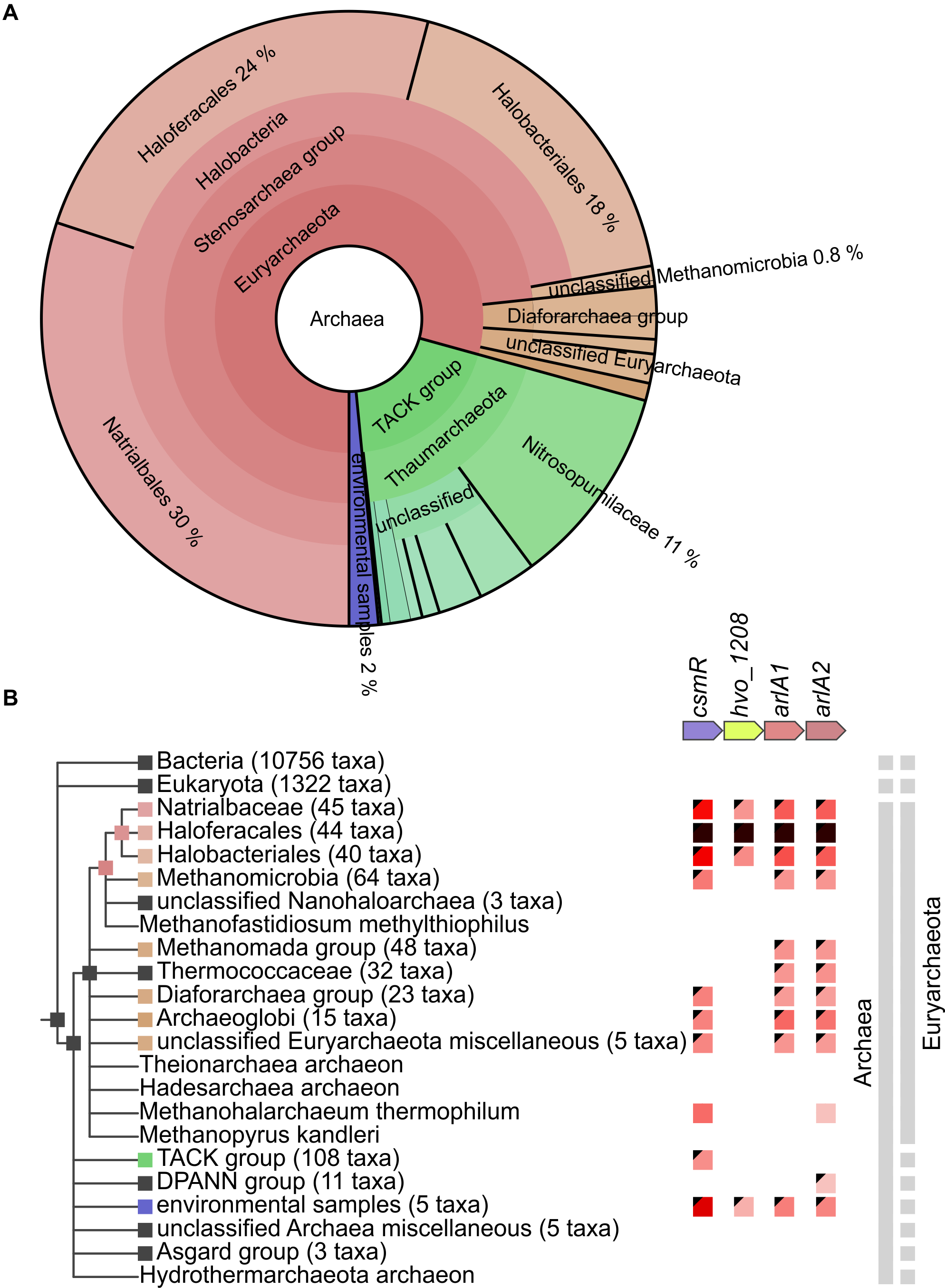

Supplement: S1 Fig — (A) The sunburst chart in depicts the taxonomic distribution of csmR-encoding species across the domain Archaea. Concentric rings represent finer taxonomic levels successively, moving outward from the domain level (center) through phylum, class, and order. Data were retrieved from the EggNOG database [81]. The angular size of each sector is proportional to the number of species within that taxon harboring a csmR ortholog. Colors distinguish major archaeal lineages: pink/red shading denotes Euryarchaeota (predominantly Halobacteria), and green shading denotes the TACK superphylum (predominantly Thaumarchaeota/Nitrosopumilaceae). Percentage values on the outermost ring indicate the fraction of all csmR-containing species assigned to each order. The predominance of red/pink sectors reflects that csmR is found almost exclusively in Halobacteria, with a secondary occurrence in Thaumarchaeota (Nitrosopumilaceae, 11%). (B) Shows a gene co-occurrence plot of csmR with the archaellin genes arlA1 and arlA2 based on the STRING database [82] (Version 12.0). The phylogenetic tree on the left (based on the STRING taxonomy) shows major archaeal lineages with Bacteria and Eukaryota as outgroups. Colored squares at internal tree nodes indicate taxonomic groupings according to colors in (A). The gene locus diagram (top right) illustrates the genomic neighborhood of csmR in H. volcanii: csmR (purple arrow), hvo_1208 (yellow arrow), and arlA1 and arlA2 (red arrows). For each taxon row, the colored squares to the right of the tree show the predicted functional co-occurrence score (as computed by STRING) for each gene relative to csmR: dark red indicates a high confidence score, pale pink a lower confidence score, and an empty cell indicates no predicted co-occurrence. Squares split diagonally with a black triangle indicate that the gene is present in the STRING reference genome(s) for that taxon, with the colored portion still reflecting the co-occurrence score. The two gray columns on [file pgen.1012198.s013.tif]

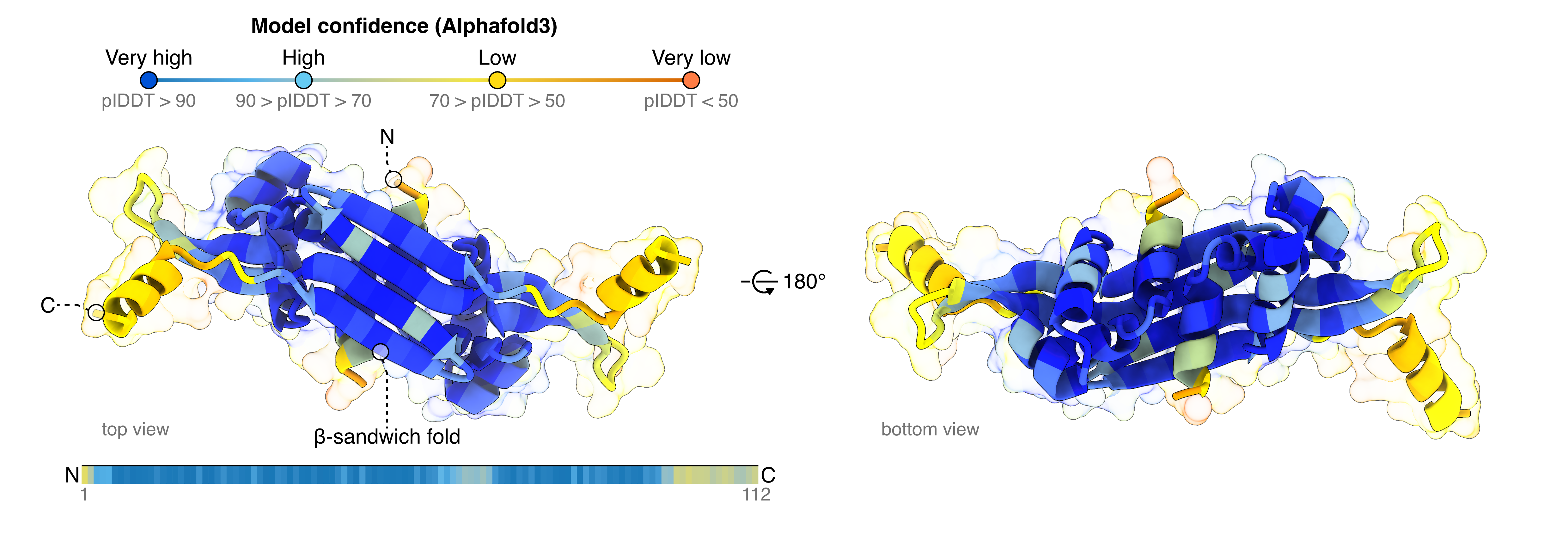

Supplement: S2 Fig — AlphaFold3 structural prediction of a CsmR dimer, shown in two orientations. The confidence of the model is color-coded from high (blue) to low (yellow/red) based on the pLDDT score (per-residue measure of local confidence). The core structure is defined by a β-sandwich fold, which is highlighted and characteristic of the Lrp/AsnC family of transcriptional regulators. The per-residue confidence score is plotted below the structure. (TIFF) [file pgen.1012198.s014.tiff]

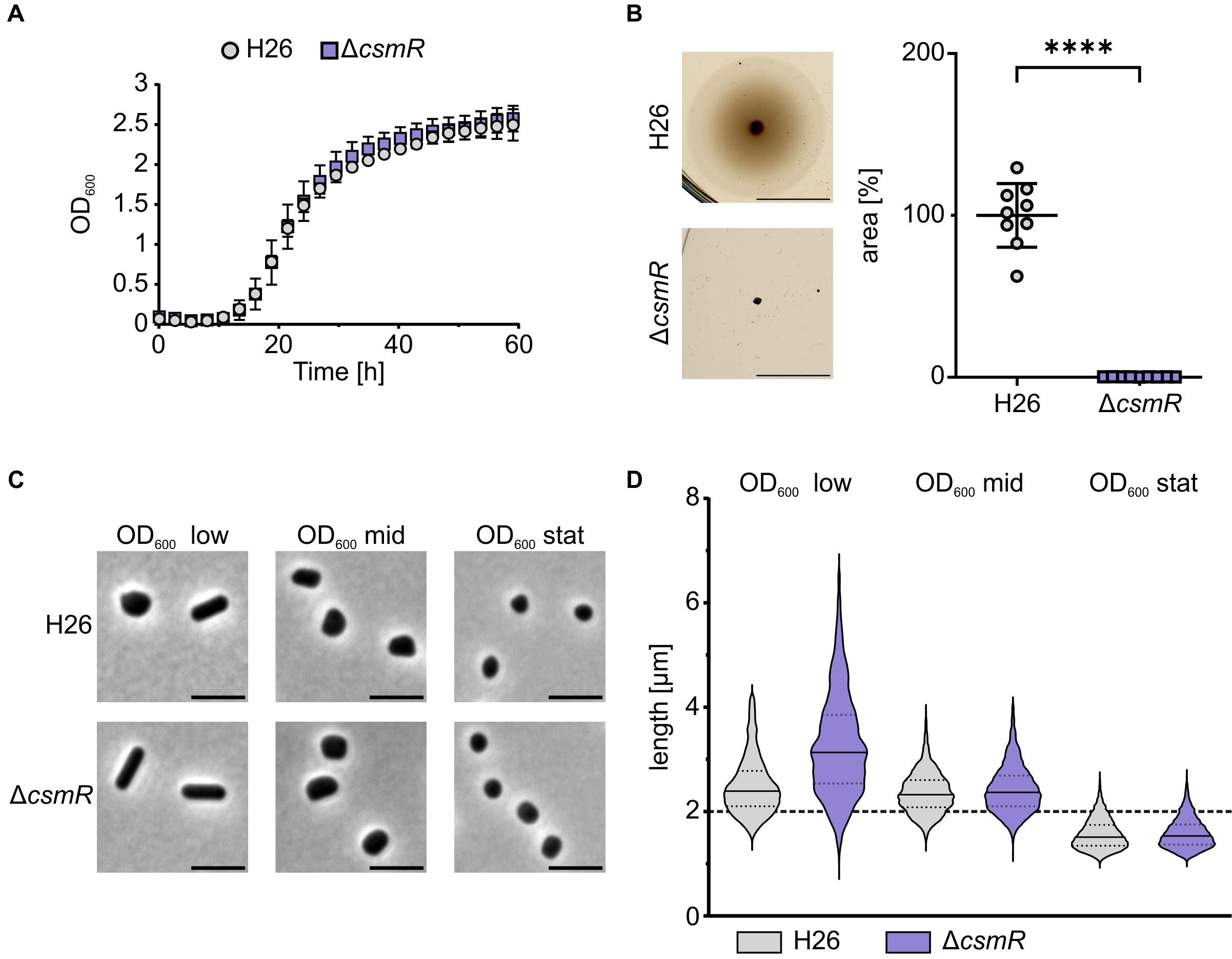

Supplement: S3 Fig — (A) Growth curve of H26 and ΔcsmR over a period of 60 h. Each strain was grown in triplicate and the mean per triplicated is plotted together with its standard deviation. (B) Motility assay comparing wild type H26 with the ΔcsmR. Exemplary motility halos of both strains are shown. The area of the motility halos was measured and normalized to the average area of wild type halos showing significantly (p ≤ 0.0001) decreased motility in the deletion strain compared to the H26 control. Samples were measured in biological and technical triplicates and all single data points per strain were plotted. The middle line indicates the mean and the upper and lower line the standard deviation. Scale bar 2 cm. (C) Cell shape analysis of the wild type H26 and the csmR deletion strain at low, mid and stationary OD600. Scale bar 4 µm. (D) Cell shape was analyzed using MicrobeJ and the summarized results of three independent biological replicates per strain and OD600 value plotted as violin-plots. The median is indicated by the middle black line, dotted lines indicate the first and third quartile. For each condition more than 1000 cells were analyzed. The dotted line indicates the length below which cells are considered plate shaped. (TIF) [file pgen.1012198.s015.tif]

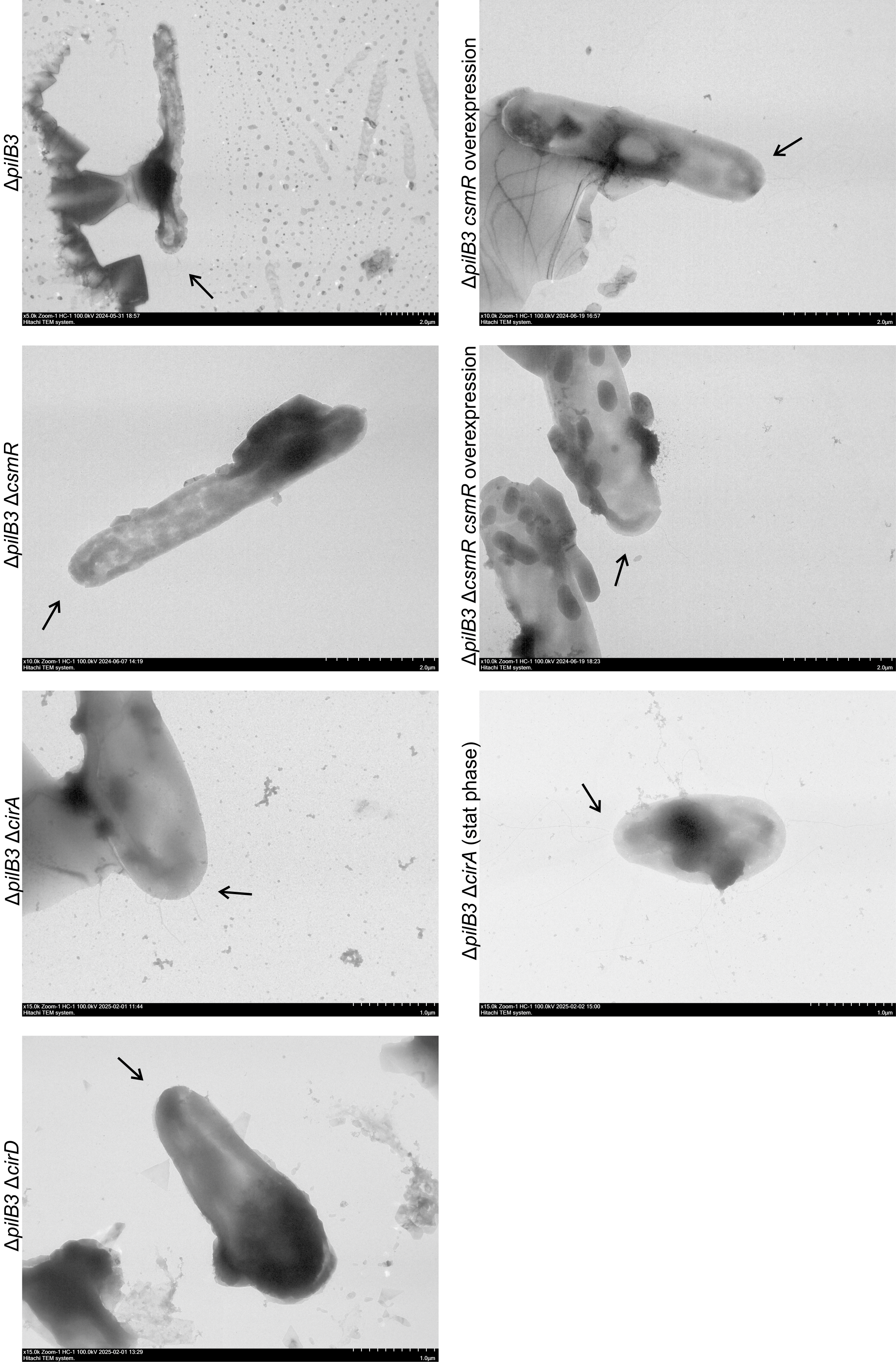

Supplement: S4 Fig — Images showing the whole cell TEM images of the different strains used. Arrows indicate the cell poles that were focused on in the main figures. (TIF) [file pgen.1012198.s016.tif]

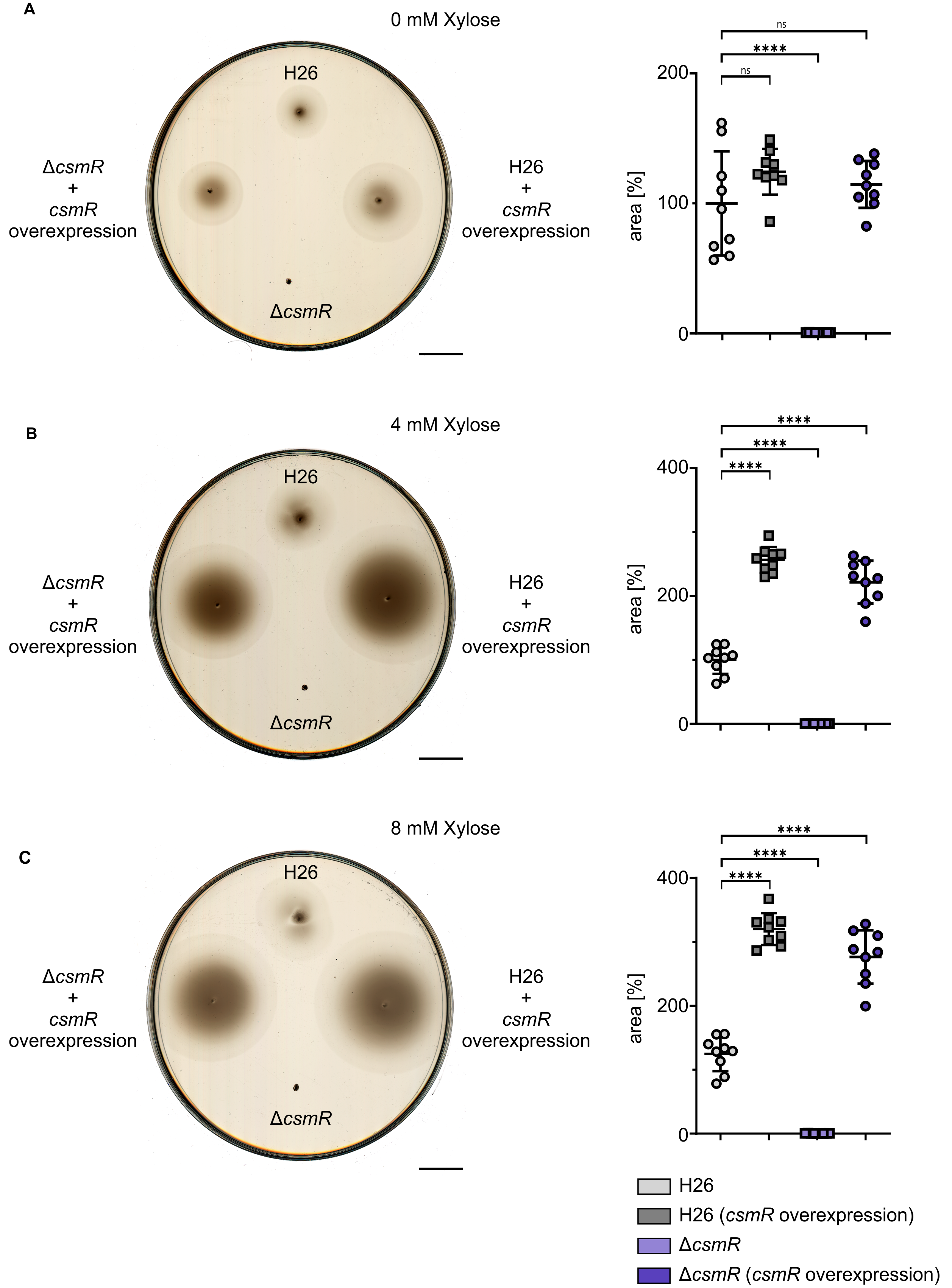

Supplement: S5 Fig — (A) 0 mM xylose: Motility assay comparing wildtype H26 with the csmR deletion strain (containing pTA1392) and both strains transformed with the csmR overexpression plasmid pSVA13922. An exemplary motility plate is shown. The area of the motility halos was measured and normalized to the average area of wildtype halos showing a significant reduction (p ≤ 0.0001) in cell locomotion of the csmR deletion strain compared to H26. Between H26, H26 + pSVA13922 and ΔcsmR + pSVA13922 there is no difference in motility showing that the overexpression plasmid already complements the deletion phenotype back to wildtype levels even without inducer. (B) 4 mM xylose: Motility assay comparing the same strains as in (A). An exemplary motility plate is shown. Increased inducer concentration led to significantly increased (p ≤ 0.0001) motility of the strains transformed with the csmR expression plasmid compared to H26. (C) 8 mM xylose: Motility assay comparing the same strains as in (A). An exemplary motility plate is shown. Further increment of the inducer led to even higher motility of the pSVA13922 containing strains. All samples were measured in biological and technical triplicates and all single data points per strain were plotted. The middle line indicates the mean and the upper and lower line the standard deviation. Scale bar 2 cm. (TIF) [file pgen.1012198.s017.tif]

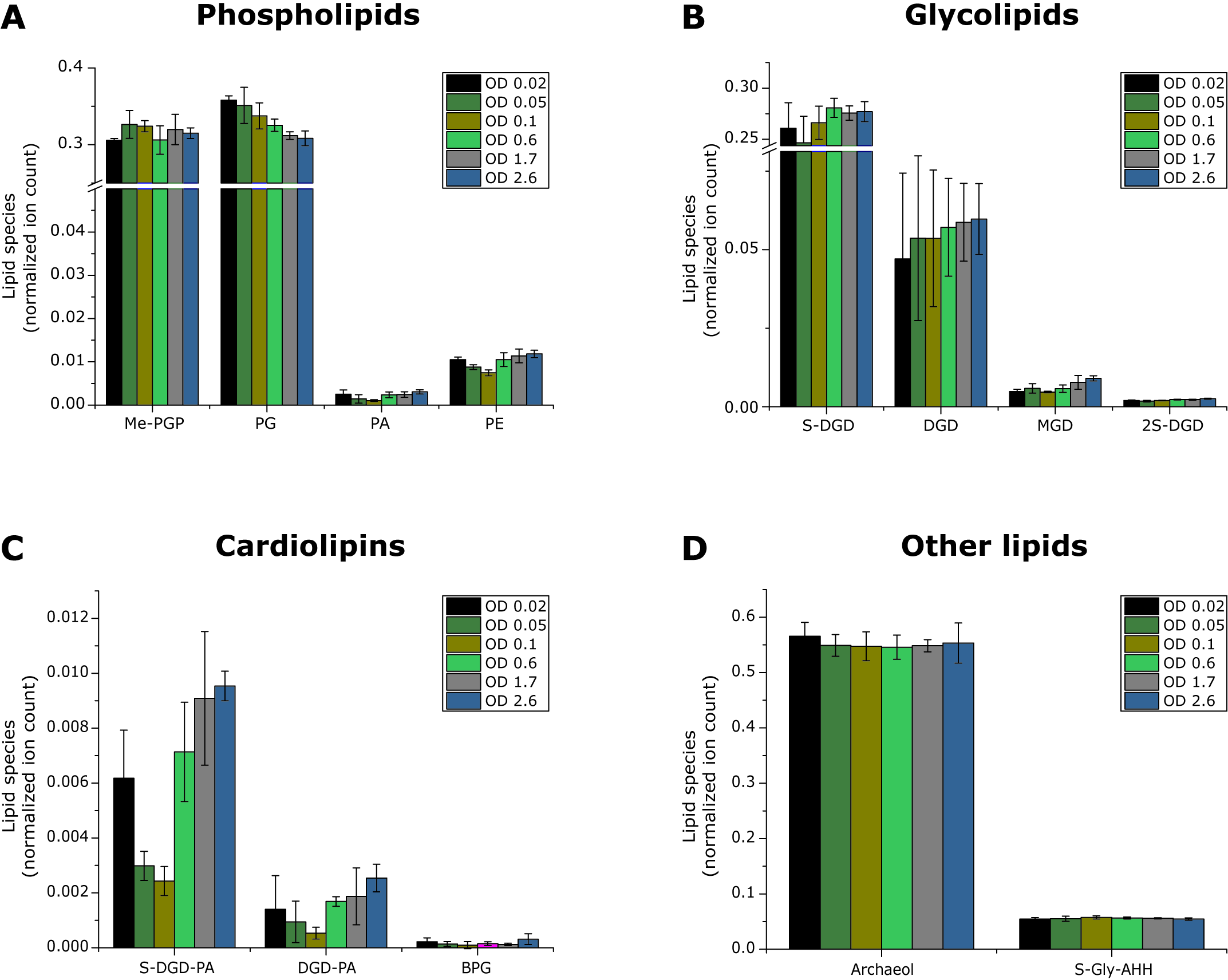

Supplement: S6 Fig — Lipid species are presented as normalized ion counts. N = 4. Presented lipid species: phosphatidylglycerophosphate methyl ester (Me-PGP), phosphatidylglycerol (PG), phosphatidic acid (PA), phosphatidylethanolamine (PE), sulfo-digalactosyldiacylglycerol (S-DGD), digalactosyldiacylglycerol (DGD), monogalactosyldiacylglycerol (MGD), disulfo-digalactosyldiacylglycerol (2S-DGD), BPG (bi-phosphatidylglycerol)/aCL (archaeal cardiolipin), S-Gly-AHH (sulphated glycosylaminohexanehexaol [83,84]). (TIF) [file pgen.1012198.s018.tif]

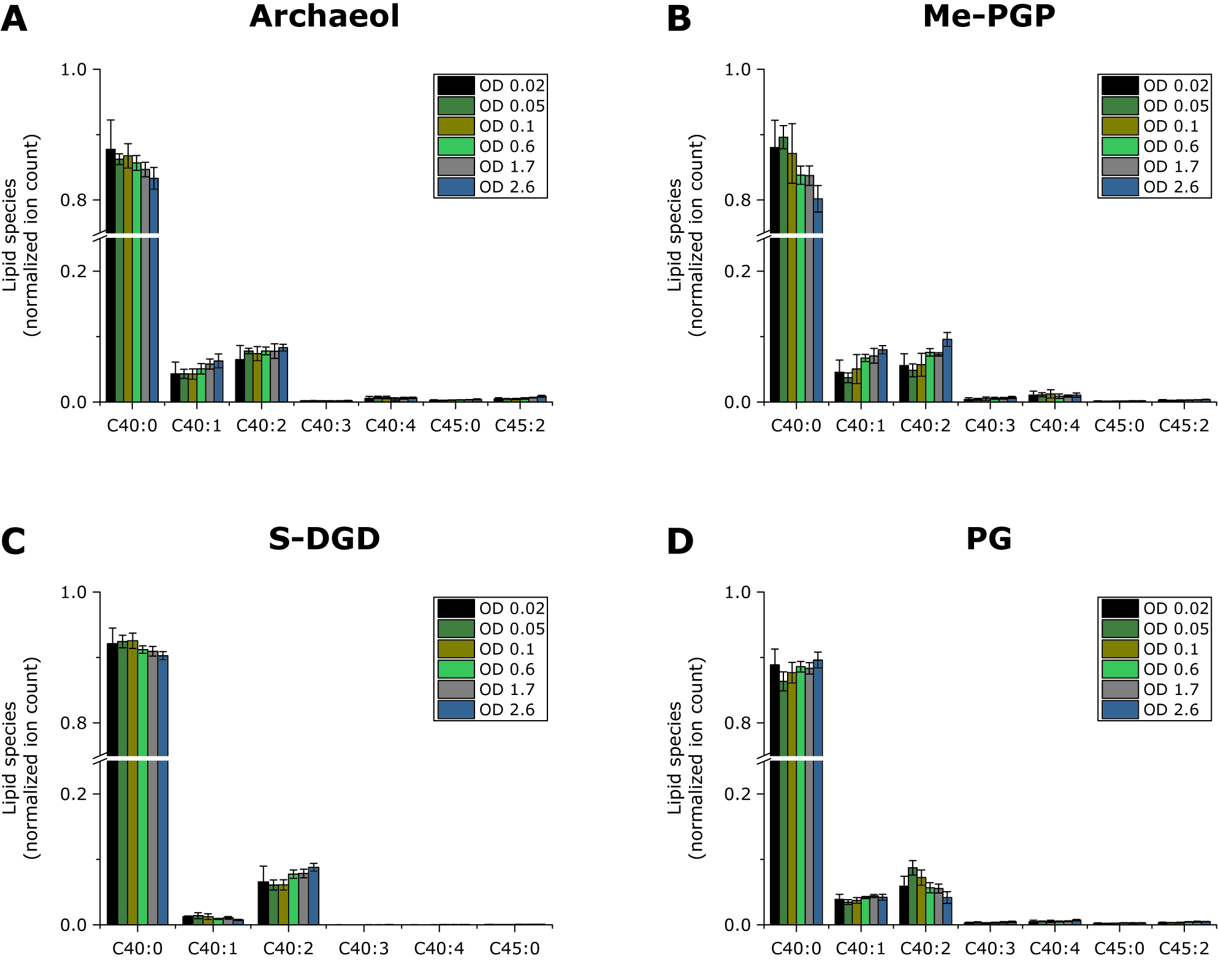

Supplement: S7 Fig — (A) archaeol, (B) phosphatidylglycerophosphate methyl ester (Me-PGP), (C) sulfo-digalactosyldiacylglycerol (S-DGD) and (D) phosphatidylglycerol (PG). Lipid species are presented as normalized ion counts. N = 4. (TIF) [file pgen.1012198.s019.tif]

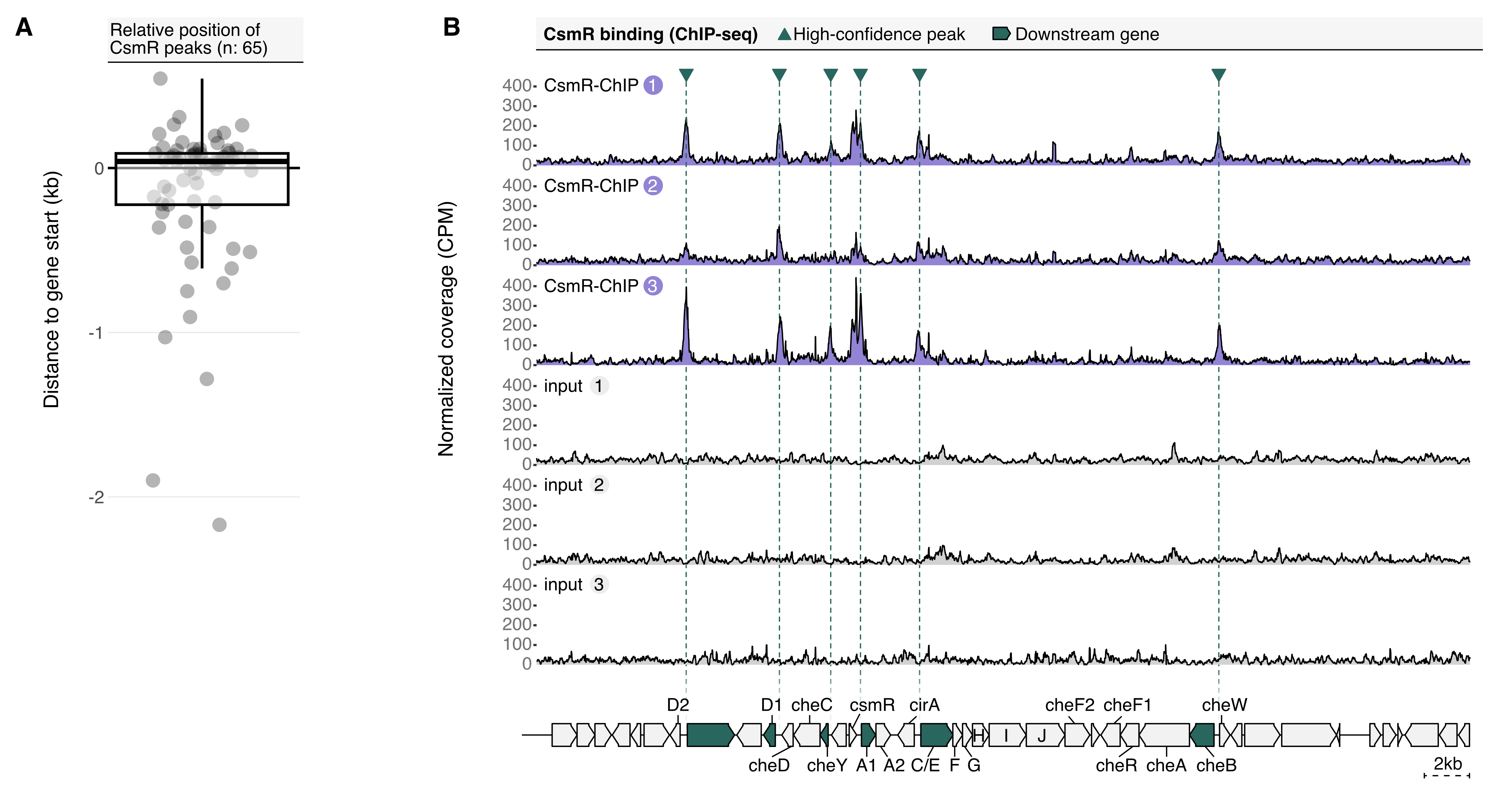

Supplement: S8 Fig — (A) Distribution of distances between CsmR ChIP-seq peak summits and annotated gene start sites. Negative values indicate upstream (promoter-proximal) localization. (B) Genome browser view showing CsmR ChIP-seq signal (three biological replicates) and corresponding input controls across a representative genomic region. High-confidence peaks are indicated by dashed lines, and associated downstream genes are marked. Gene annotations are shown below. (TIF) [file pgen.1012198.s020.tif]

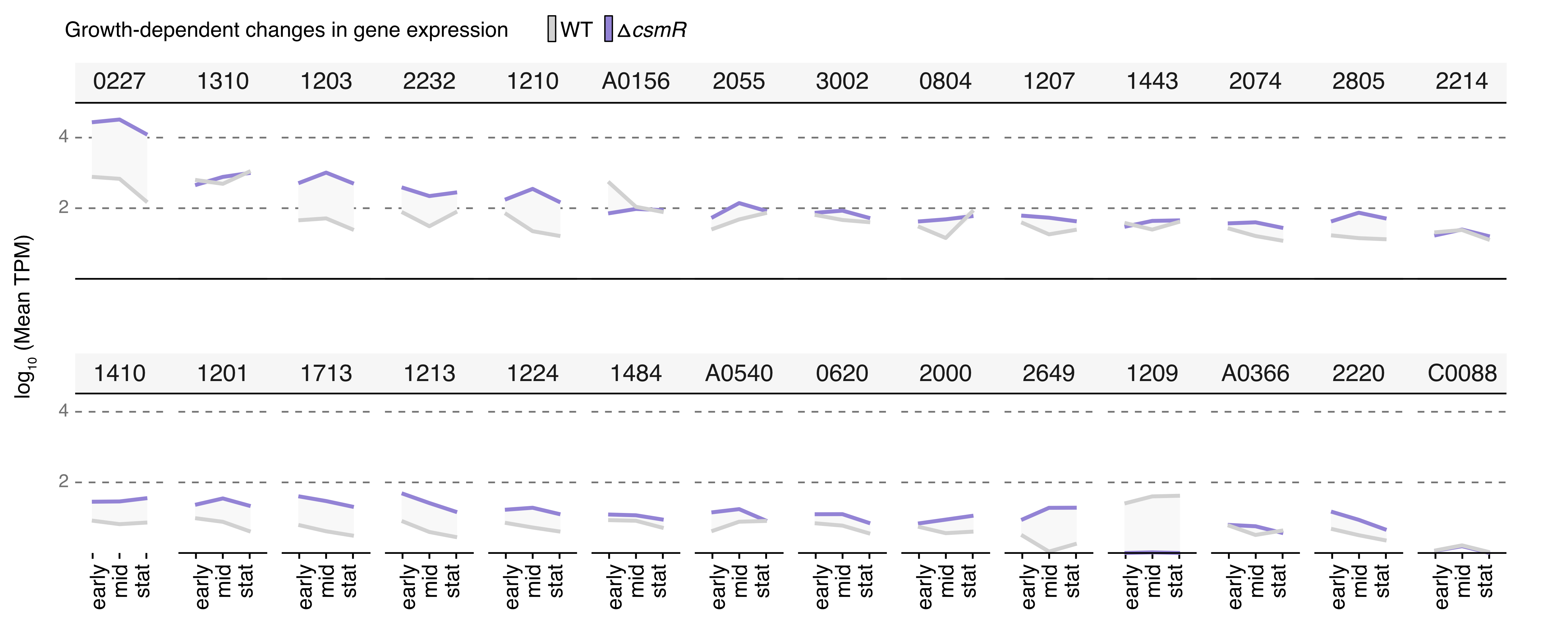

Supplement: S9 Fig — Gene expression is shown as log₁₀(mean TPM + 1) across growth phases (early, mid, stationary) for wild-type (H26) and ∆csmR strains. Each panel represents an individual gene. Lines indicate mean expression across replicates. (TIF) [file pgen.1012198.s021.tif]

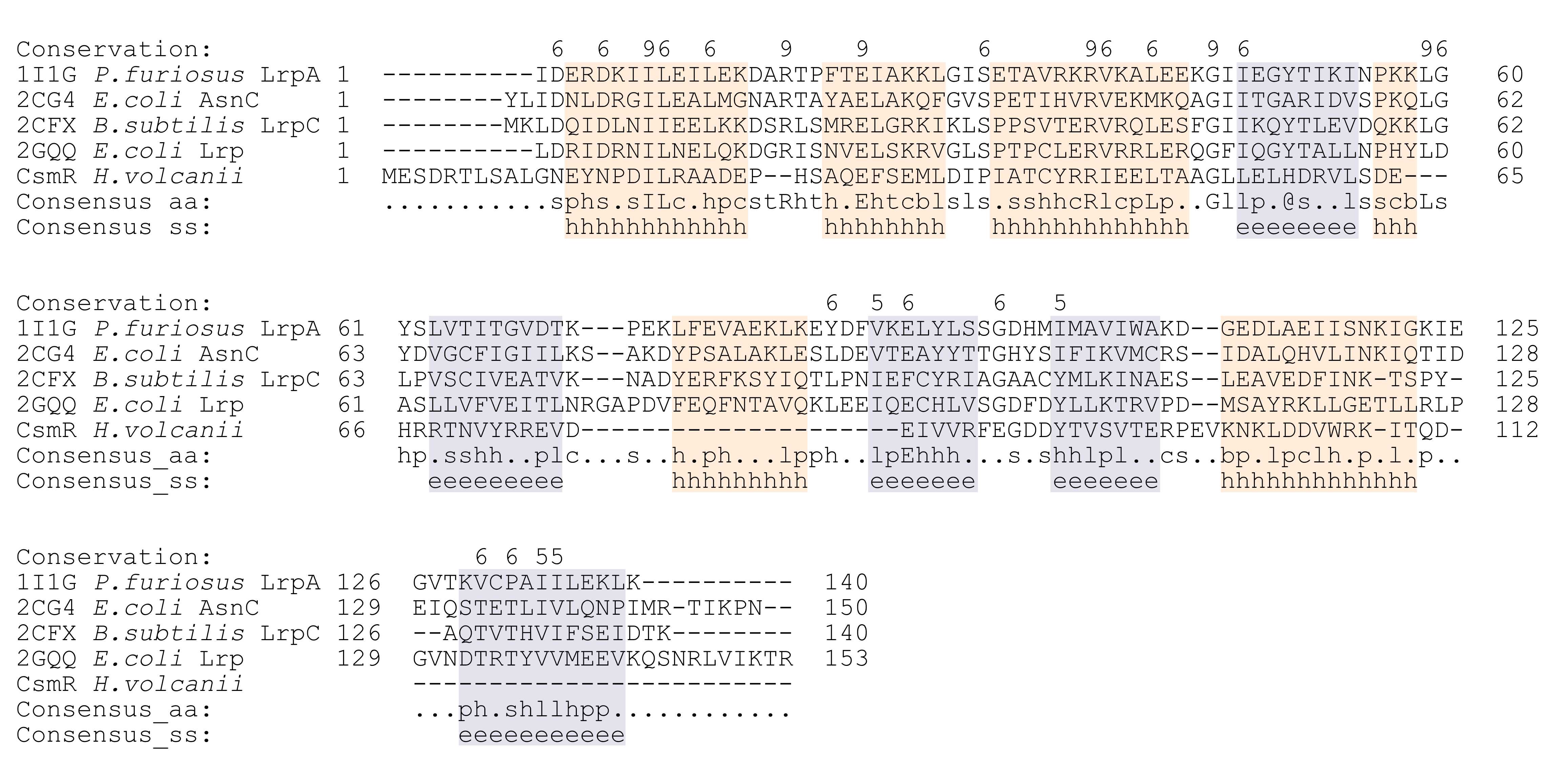

Supplement: S10 Fig — CsmR was aligned with Pyrococcus furiosus LrpA (1I1G), Escherichia coli AsnC (2 CG4), Bacillus subtilis LrpC (2CFX), and E. coli Lrp (2GQQ) using a structure-guided PROMALS3D alignment. The strongest correspondence is concentrated in the predicted N-terminal DNA-binding region of CsmR, whereas the central/C-terminal part is shorter and more divergent. In addition, the C-terminal extension present in canonical Lrp/AsnC regulators is absent in CsmR. Colored boxes indicate predicted secondary-structure elements in the alignment (orange, α-helices; light blue, β-strands). (TIF) [file pgen.1012198.s022.tif]

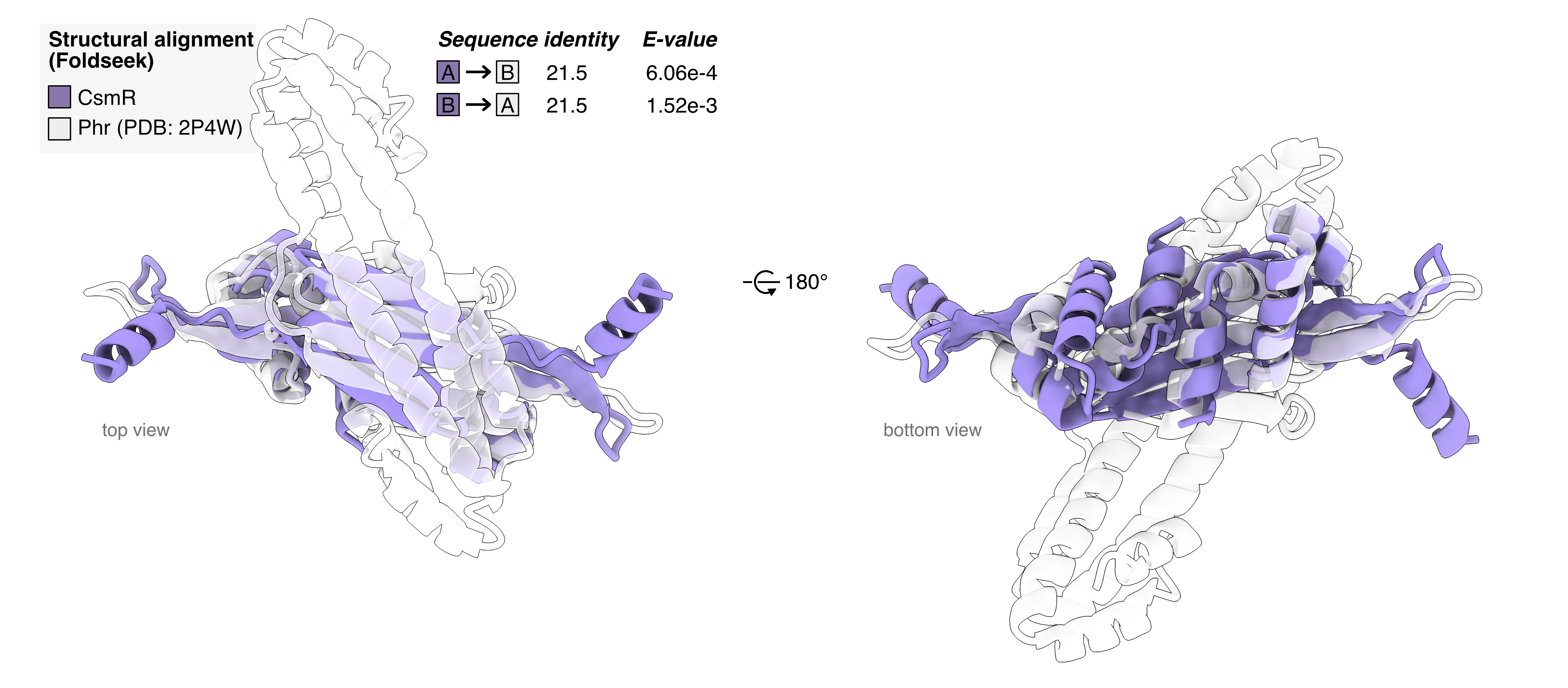

Supplement: S11 Fig — CsmR (purple) and Phr (gray) are shown in two orientations to highlight structural similarity. Despite low sequence identity (21.5%), structural alignment reveals a conserved core fold, particularly in the β-sandwich domain, suggesting a shared architectural framework. The E-values indicate statistically significant similarity. (TIFF) [file pgen.1012198.s023.tiff]

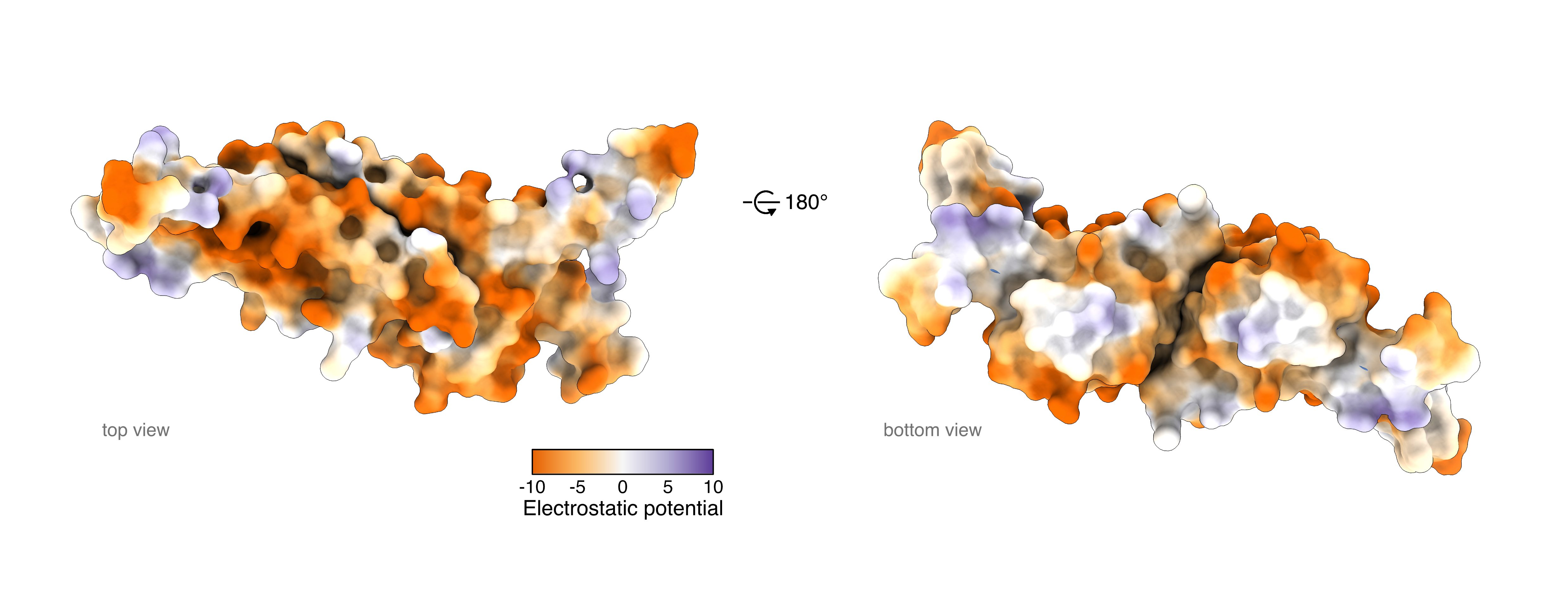

Supplement: S12 Fig — Electrostatic surface representation of CsmR shown in two orientations (top and bottom view). The color gradient represents the electrostatic potential, ranging from negatively charged regions (orange) to positively charged regions (purple). (TIFF) [file pgen.1012198.s024.tiff]

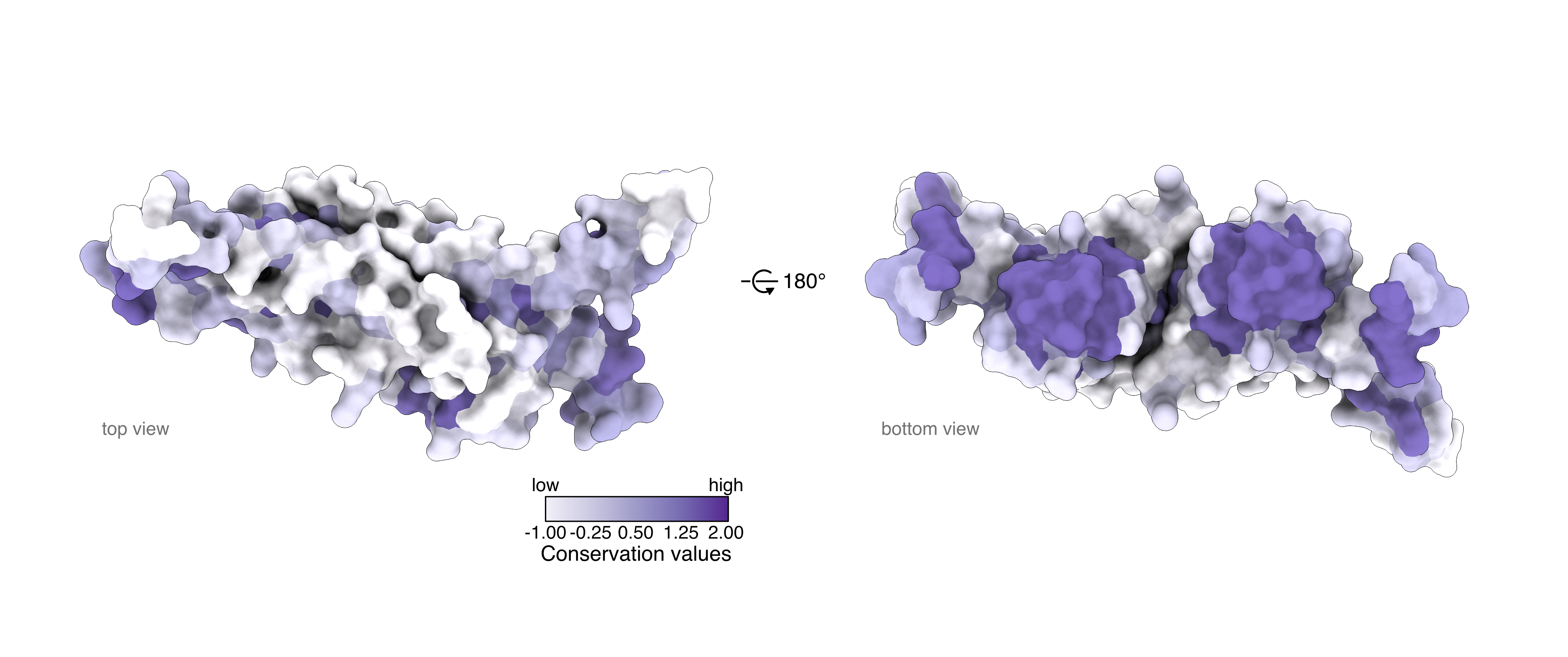

Supplement: S13 Fig — Surface representation of CsmR colored by sequence conservation across halobacterial homologs, based on entropy-based conservation scores from AL2CO. Highly conserved residues are shown in dark purple, while variable regions are in white. (TIFF) [file pgen.1012198.s025.tiff]

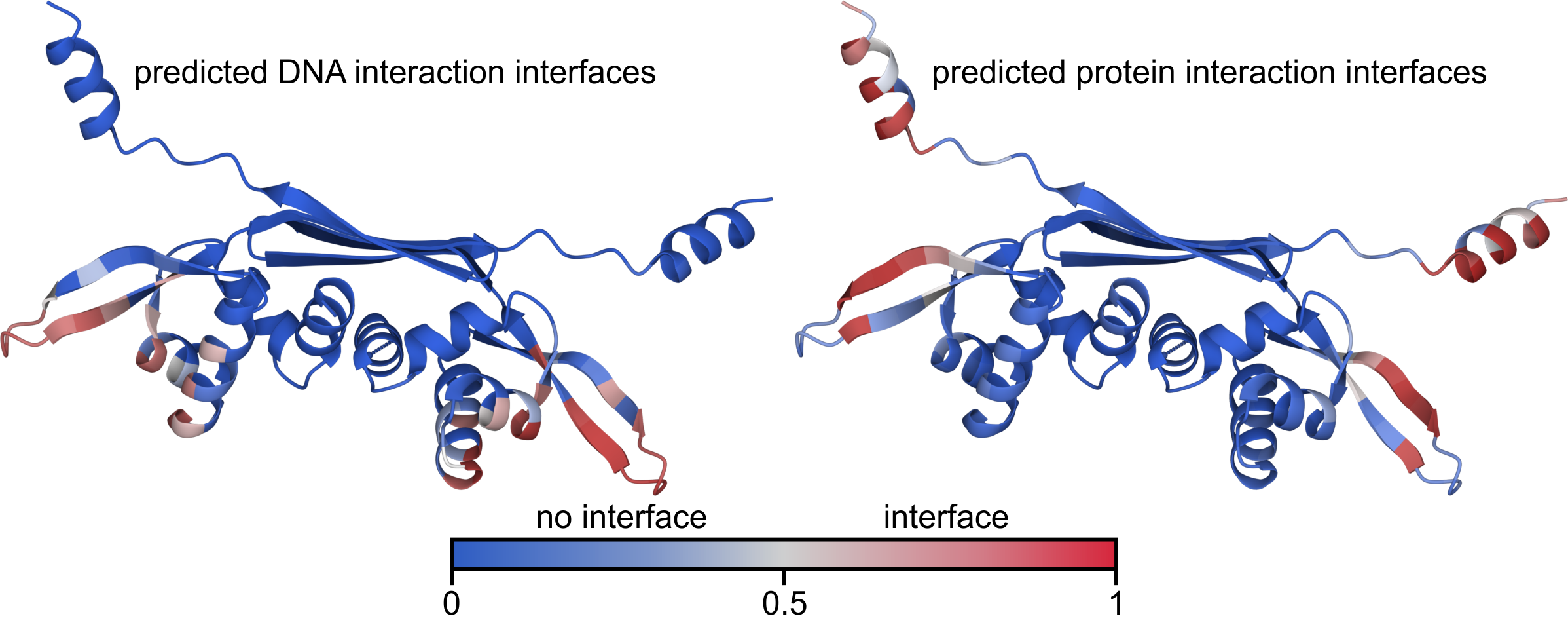

Supplement: S14 Fig — DNA-protein and protein-protein interface prediction of the CsmR dimer retrieved from Alphafold3 prediction. Prediction confidence is shown by a color gradient from blue, no interface, to red for interface. (TIF) [file pgen.1012198.s026.tif]

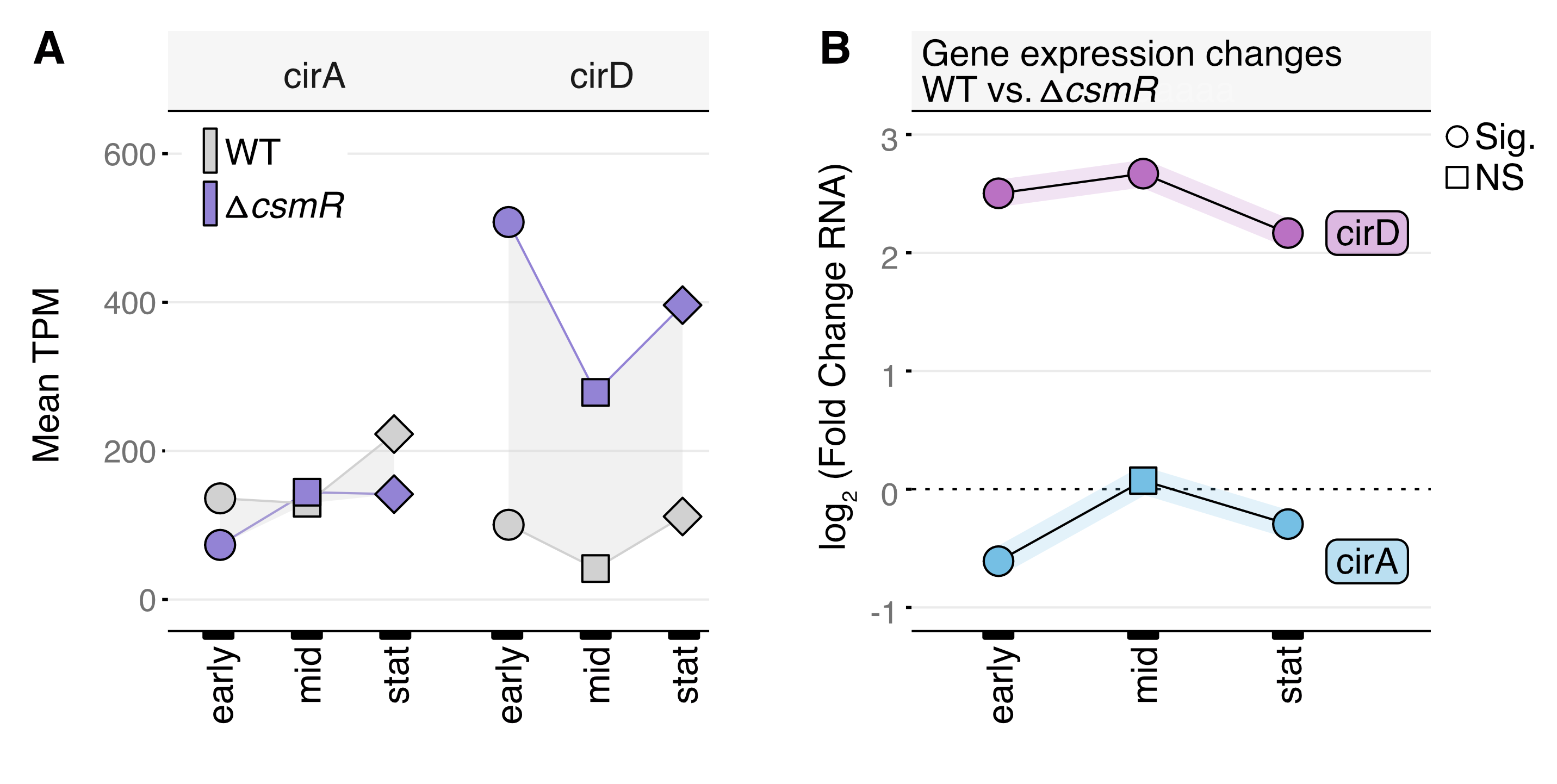

Supplement: S15 Fig — (A) Mean transcripts per million (TPM) values of cirA and cirD across early (circle), mid (square), and stationary (diamond) growth phases in WT (grey) and ∆csmR (purple) strains. (B) Log2 fold-change in RNA expression (∆csmR vs. WT) for cir genes across growth phases. Significant genes are depicted as circles, and non-significant genes as squares. Color indicates specific cir genes (cirA: light blue, cirD: purple). (TIFF) [file pgen.1012198.s027.tiff]

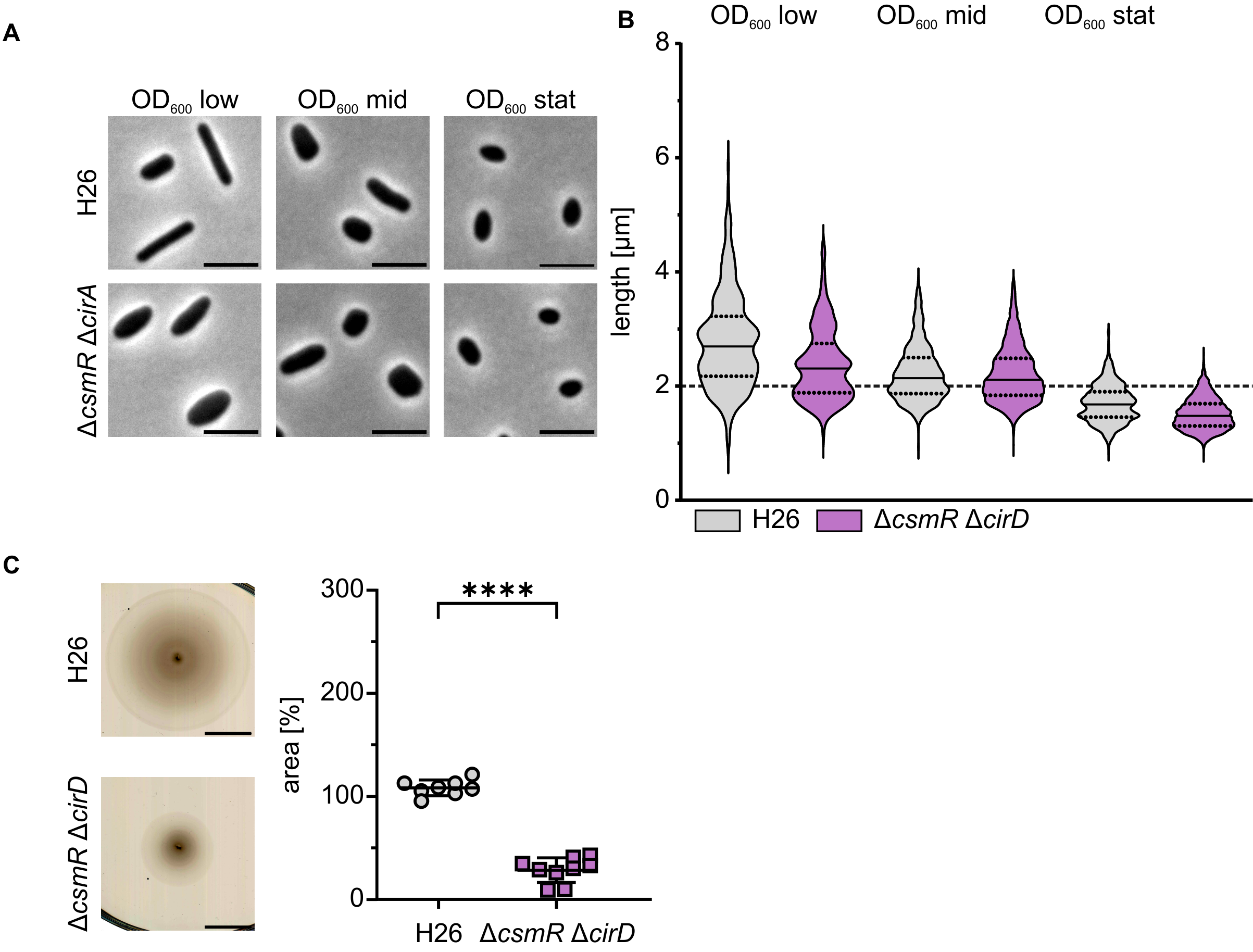

Supplement: S16 Fig — (A). Cell shape analysis of the wild type H26 + pTA1392 and the csmR cirD + pTA1392 double deletion strain at low, mid and stationary OD600. Scale bar 4 µm. (B) Cell shape was analyzed using MicrobeJ and the summarized results of three independent biological replicates per strain and OD600 value plotted as violin-plots. The median is indicated by the middle black line, dotted lines indicate the first and third quartile. For each condition more than 1000 cells were analyzed. The dotted line indicates the length below which cells are considered plate shaped. (C) Motility assay of H26 + pTA1392 and the csmR cirD + pTA1392 double deletion strain. Exemplary motility halos of both strains are shown. The area of the motility halos was measured and normalized to the average area of wild type halos showing significantly (p ≤ 0.0001) decreased motility in the double deletion strain compared to the H26 control. Samples were measured in biological and technical triplicates and all single data points per strain were plotted. The middle line indicates the mean and the upper and lower line the standard deviation. Scale bar 2 cm. (TIF) [file pgen.1012198.s028.tif]

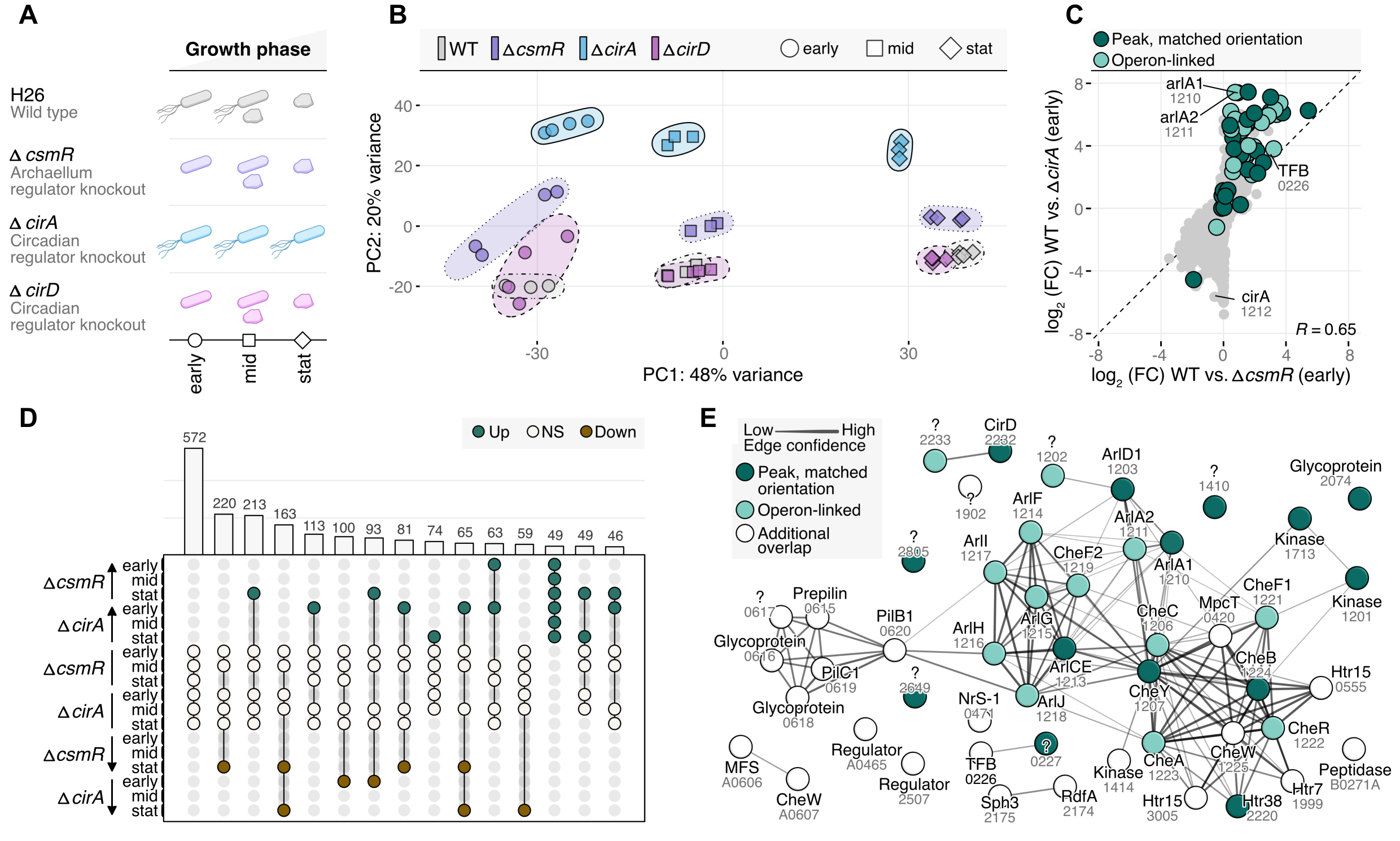

Supplement: S17 Fig — (A), Morphological changes in wild type (H26), ΔcsmR, ΔcirA, and ΔcirD strains during early, mid, and stationary growth phases. Wild type cells transition between motile rod-shaped and sessile plate-like morphologies, while ΔcsmR and ΔcirD exhibit growth phase-dependent shape changes, and ΔcirA remains rod-shaped across all phases. (B), Principal component analysis (PCA) of transcriptomic data from wild type and deletion strains, showing variance explained by principal components 1 (PC1; 48%) and 2 (PC2; 20%). Biological replicates are color-coded by strain and growth phase (circles: early, squares: mid, diamonds: stationary). (C), Pairwise comparison of fold changes in WT vs. ΔcirA and WT vs. ΔcsmR. Genes associated with CsmR ChIP-seq peaks are highlighted, with motif-containing peaks in matched orientation (dark green) and operon-linked genes (light green). Other genes are shown in grey. (D), UpSet plot showing the overlap of differentially regulated genes (padj < 0.1) across strains (∆csmR, ∆cirA) and growth phases (early, mid, stationary). Each row corresponds to a specific condition, and connected dots indicate gene sets shared between conditions. Bars represent the number of genes in each intersection. Colors indicate direction of regulation (up, down, or not significant). The analysis highlights a core set of genes consistently upregulated across all conditions. (E), STRING network analysis of overlapping genes, including archaella and chemotaxis components, revealing a highly interconnected regulatory network. High-confidence edges (confidence indicated by line width) indicate functional associations. (TIF) [file pgen.1012198.s029.tif]

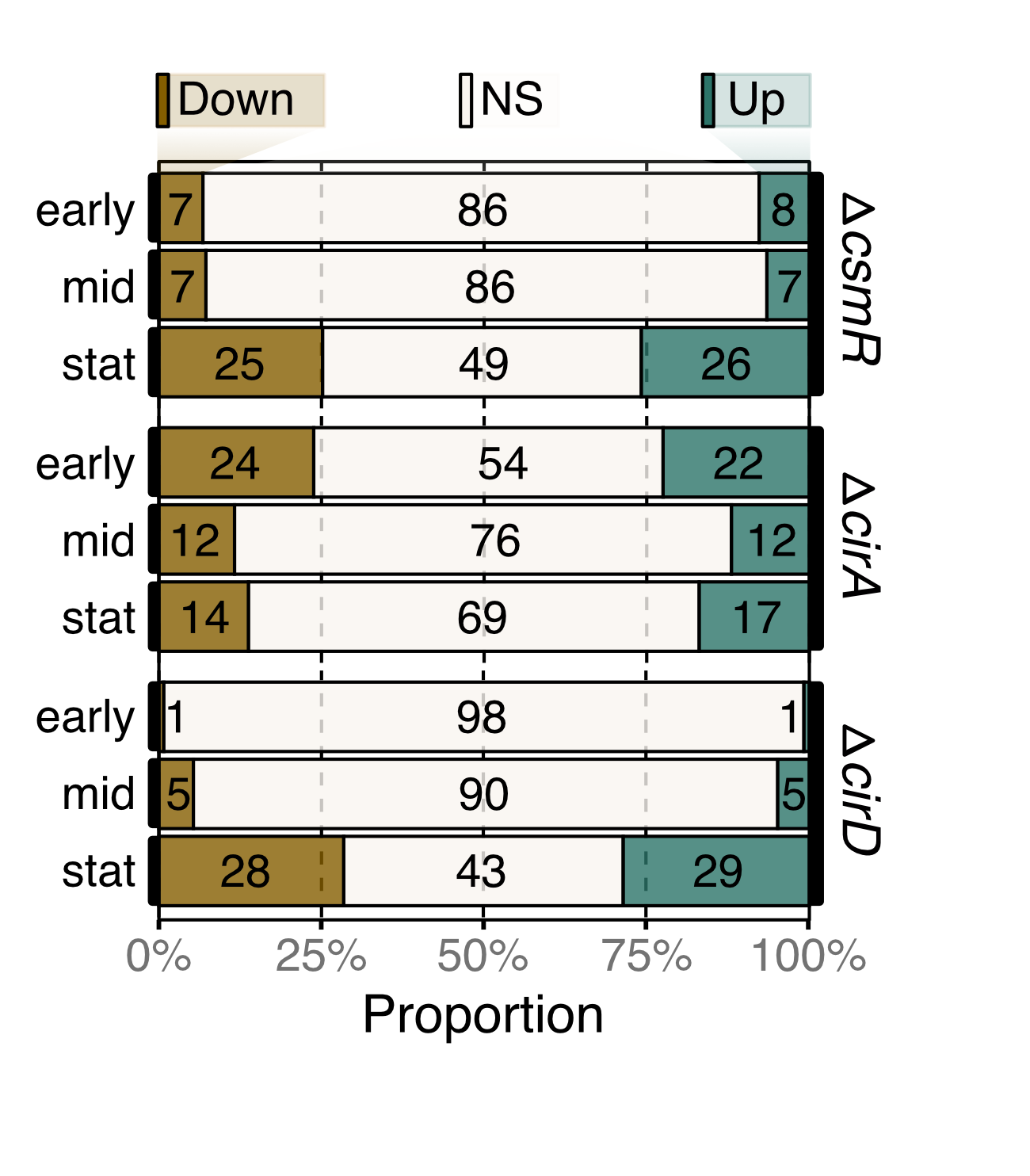

Supplement: S18 Fig — Proportions of differentially expressed genes (upregulated, downregulated, or non-significant) in ∆csmR, ∆cirA, and ∆cirD strains compared to wild type across early, mid, and stationary growth phases. Bar segments indicate the percentage of genes in each category, with upregulated genes in green, downregulated genes in brown, and non-significant genes in beige. (TIFF) [file pgen.1012198.s030.tiff]

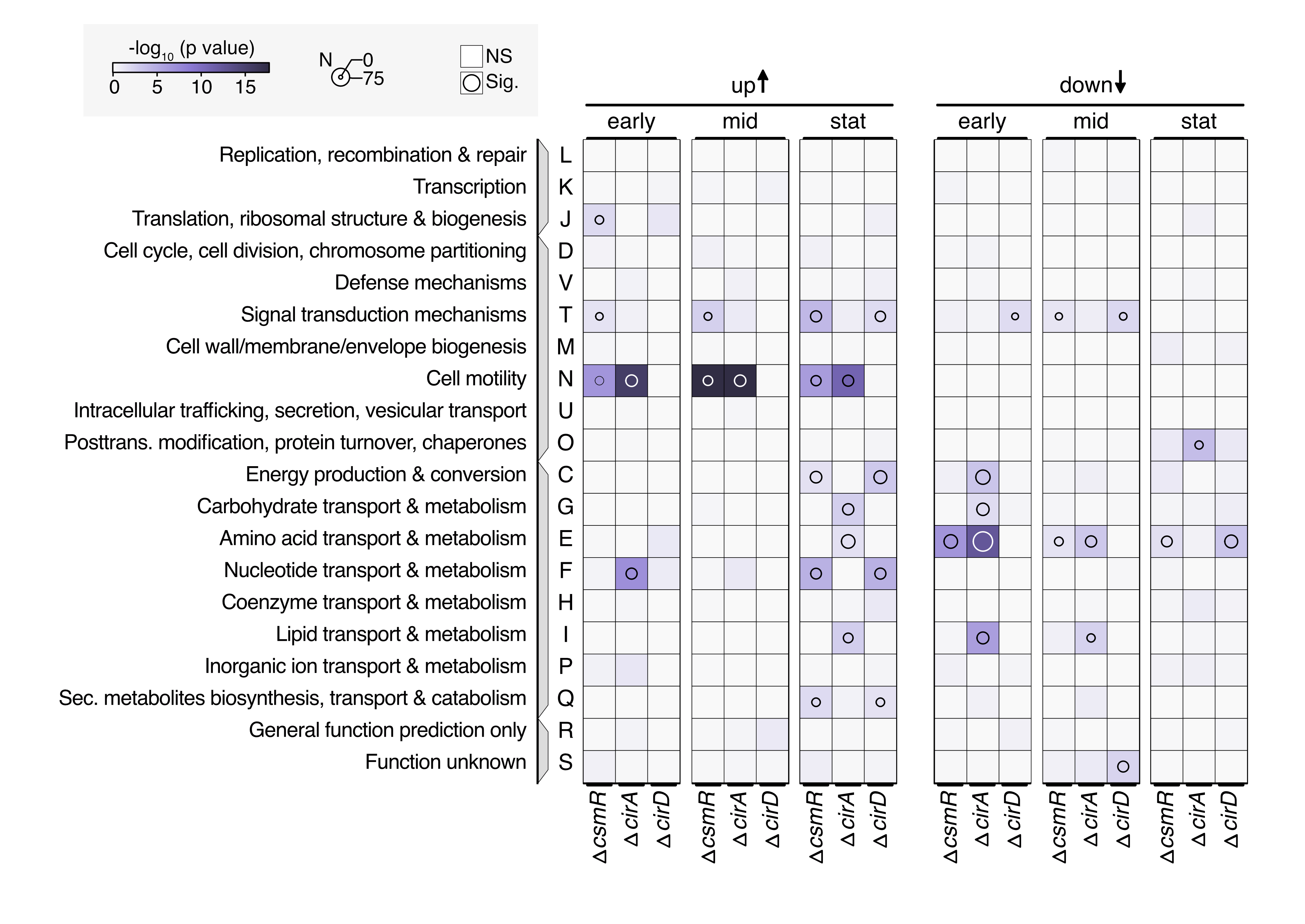

Supplement: S19 Fig — Gene set enrichment analysis of archaeal clusters of orthologous groups (arCOGs) across all deletion strains and growth phases. Circles indicate categories with raw overrepresentation P-value < 0.05, and color intensity reflects -log10(raw overrepresentation P-value). Separate analyses were performed for upregulated (padj < 0.05, log2FC >= 1) and downregulated (padj < 0.05, log2FC <= -1) genes across early, mid, and stationary growth conditions. (TIFF) [file pgen.1012198.s031.tiff]

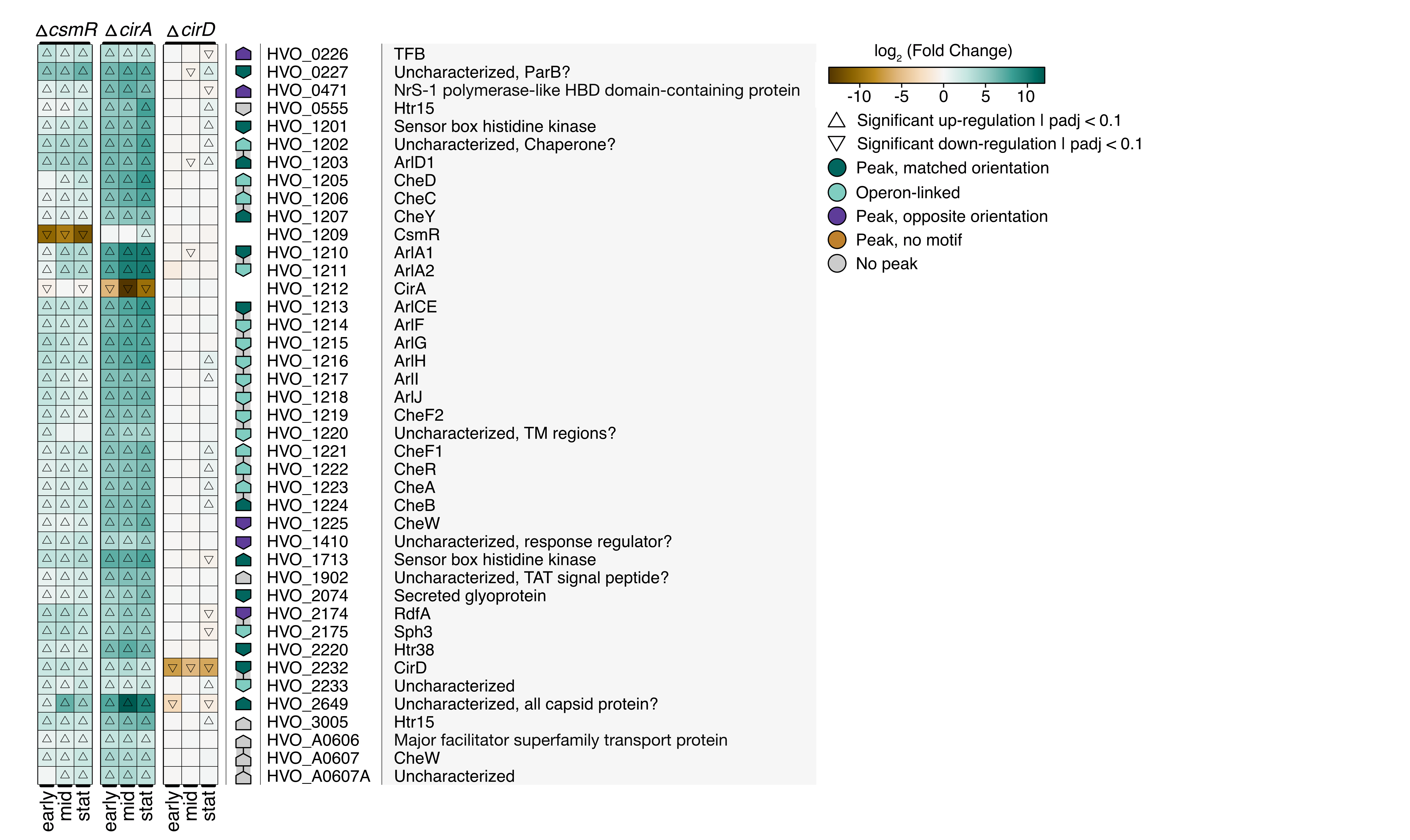

Supplement: S20 Fig — Log2 fold-change analysis (color-coded from brown to green) of genes in ΔcsmR, ΔcirA, and ΔcirD strains across early, mid, and stationary phases. Significant upregulation (up triangle) and downregulation (down triangle) are shown. Genes associated with CsmR peaks are highlighted and grouped based on binding characteristics (motif presence, strand orientation, and promoter distance. (TIF) [file pgen.1012198.s032.tif]

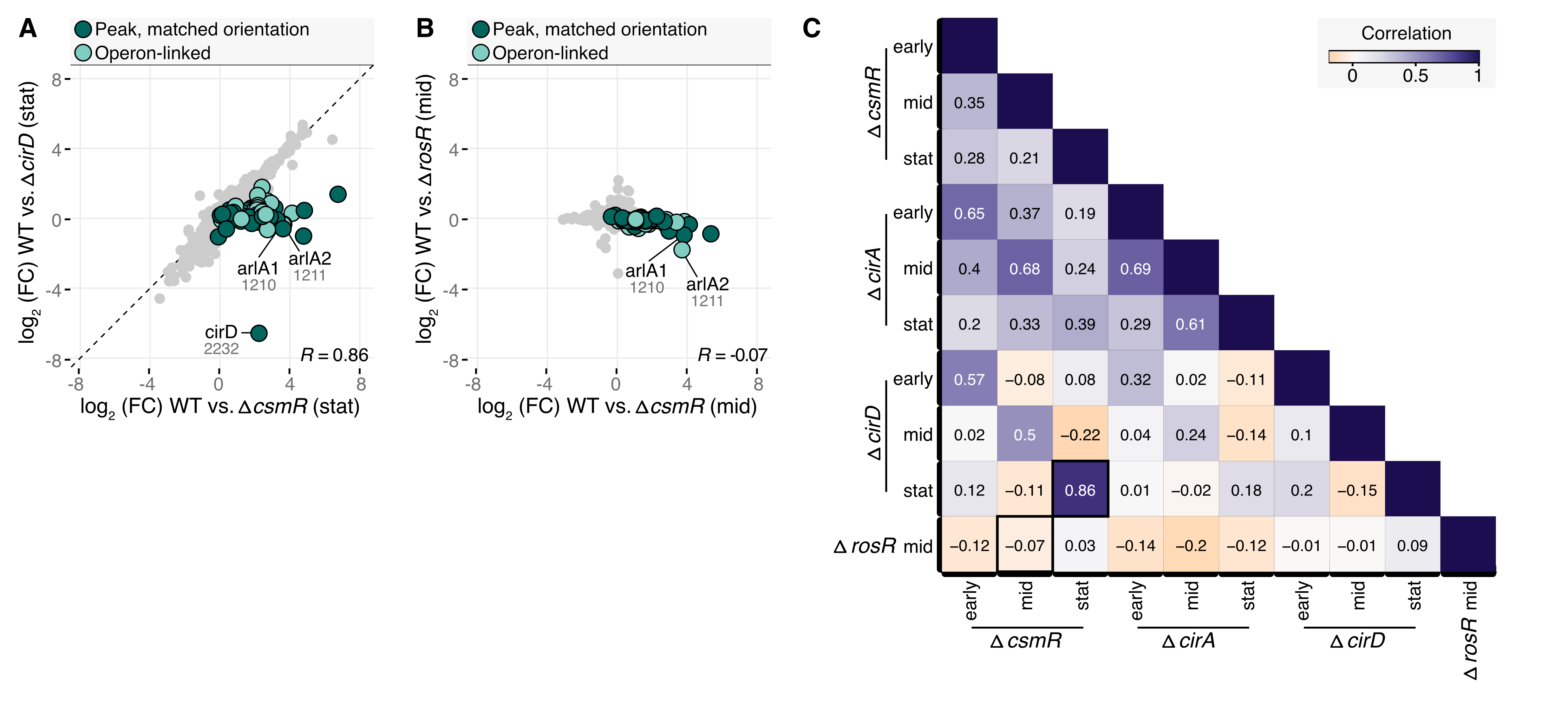

Supplement: S21 Fig — (A) Scatter plot comparing log2 fold changes (WT vs. ΔcsmR and WT vs. ΔcirD) during the stationary phase and (B) WT vs. ΔcsmR and WT vs. ΔrosR during the mid-phase. Pearsons’s correlation was calculated based on pairwise complete observations. Genes associated with CsmR ChIP-seq peaks are highlighted, with motif-containing peaks in matched orientation (dark green) and operon-linked genes (light green). Other genes are shown in grey. (C) Heatmap of correlation coefficients for log2 fold changes across strains (ΔcsmR, ΔcirA, ΔcirD, ΔrosR) and growth phases (early, mid, stationary). (TIF) [file pgen.1012198.s033.tif]

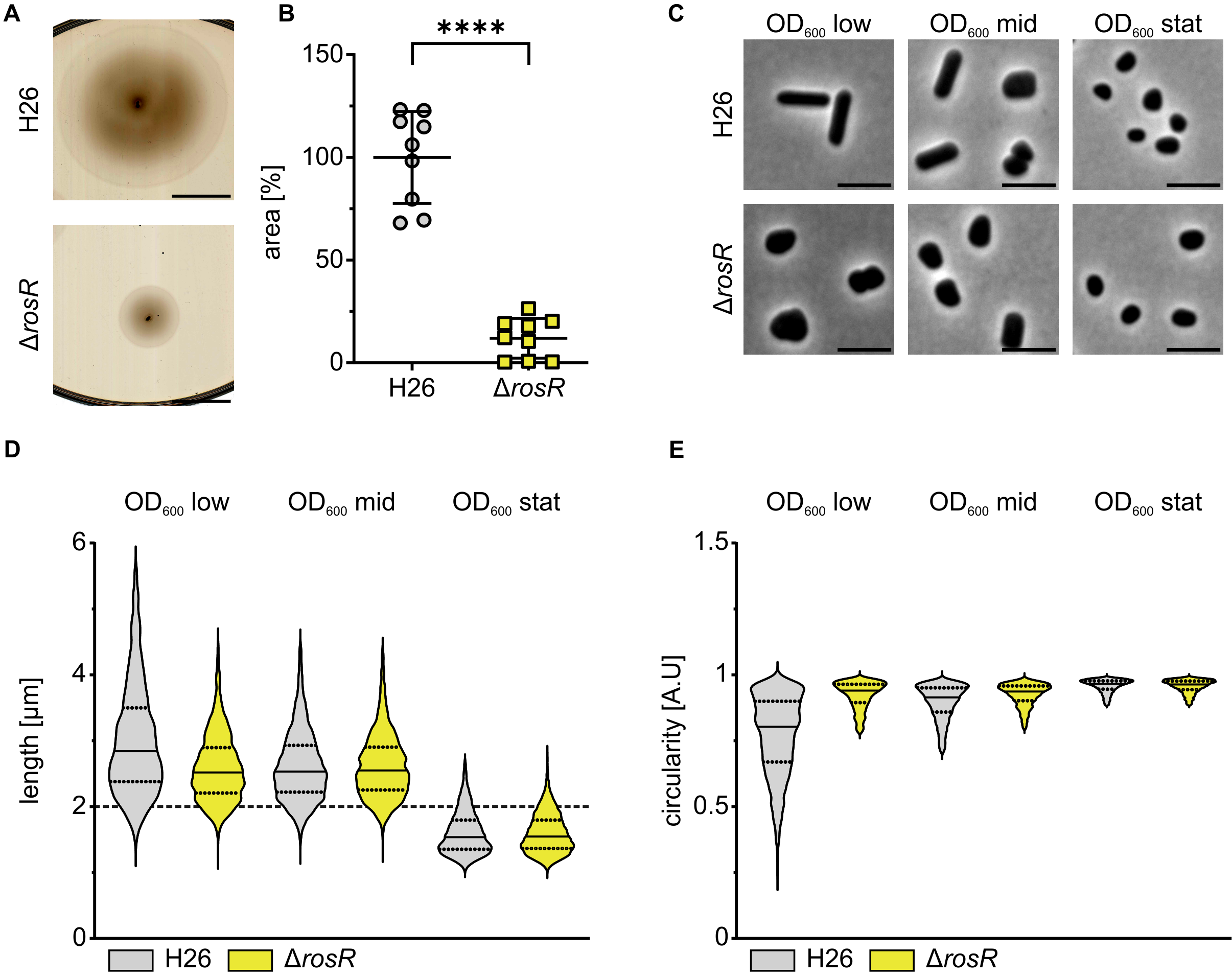

Supplement: S22 Fig — (A) Motility assay comparing wild type H26 + pTA1392 with the rosR + pTA1392 deletion strain. Exemplary motility halos of both strains are shown. Scale bar 2 cm. (B) The area of the motility halos was measured and normalized to the average area of wild type halos showing significantly (p ≤ 0.0001) decreased motility of the rosR deletion strain compared to H26. Samples were measured in biological and technical triplicates and all single data points per strain were plotted. The middle line indicates the mean and the upper and lower line the standard deviation. (C) Exemplary images of the wild type H26 + pTA1392 and the rosR + pTA1392 deletion strain at low, mid and stationary OD600. Deletion of rosR inhibits rod formation. Scale bar 4 µm. (D) Cell shape was analyzed using MicrobeJ and the summarized results of three independent biological replicates per strain and OD600 value plotted as violin-plots. The median is indicated by the middle black line, dotted lines indicate the first and third quartile. The dotted line indicates the length below which cells are considered plate shaped. (E) Cell circularity was analyzed using MicrobeJ and the summarized results of three independent biological replicates per strain and OD600 value plotted as violin-plots. The median is indicated by the middle black line, dotted lines indicate the first and third quartile. For each condition more than 1000 cells were analyzed. (TIF) [file pgen.1012198.s034.tif]

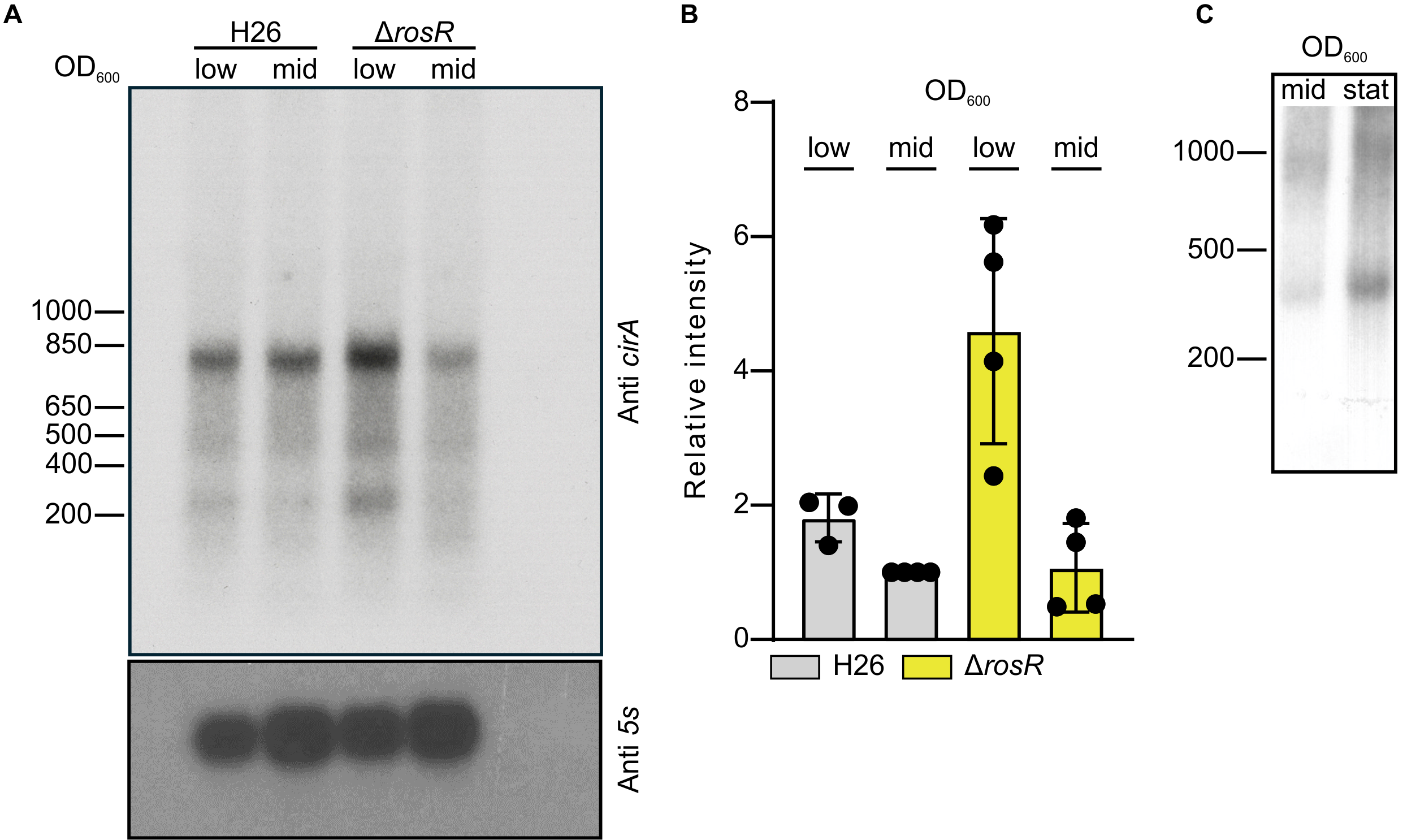

Supplement: S23 Fig — (A) Northern blot analysis using an anti-cirA probe on RNA extracted from the wild type H26 and the rosR deletion strain at low (OD600 0.02) and mid (OD600 0.2) growth. Anti 5S probe was used as loading control. (B) Quantification of the signal by the cirA probe normalized to the 5S signal. Plotted are the results from at least three independent experiments per strain and growth phase. (C) Detection of the hvo_1211s transcript using RNA extracted from H26 on a Northern blot with a size of approximately 400 nucleotides. (TIF) [file pgen.1012198.s035.tif]

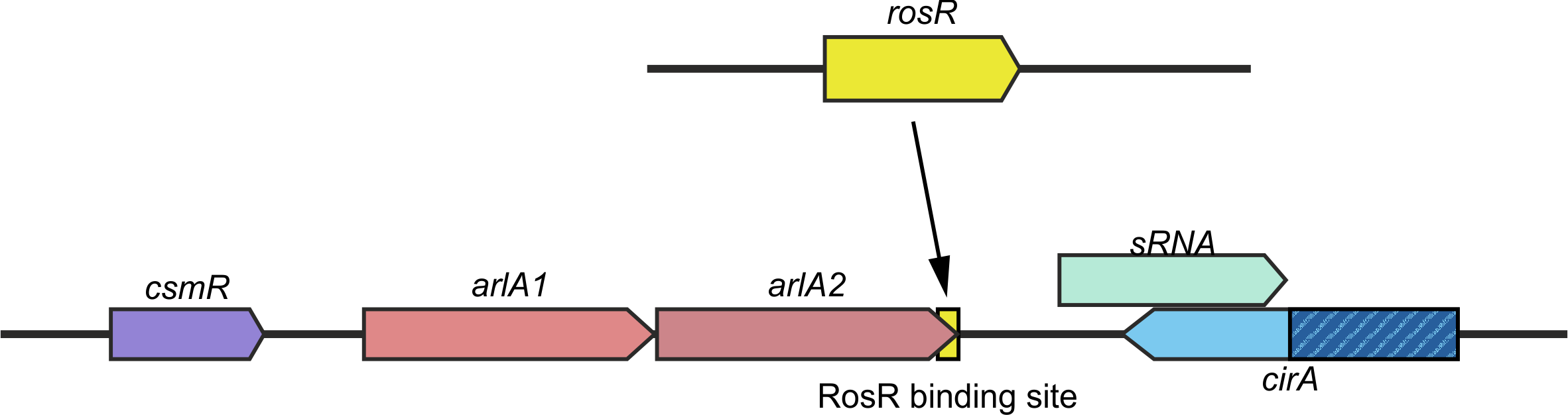

Supplement: S24 Fig — RosR binding to the 3’ end of arlA2 is indicated by a yellow box. The removed part of the partial cirA deletion is indicated in dark blue. (TIF) [file pgen.1012198.s036.tif]

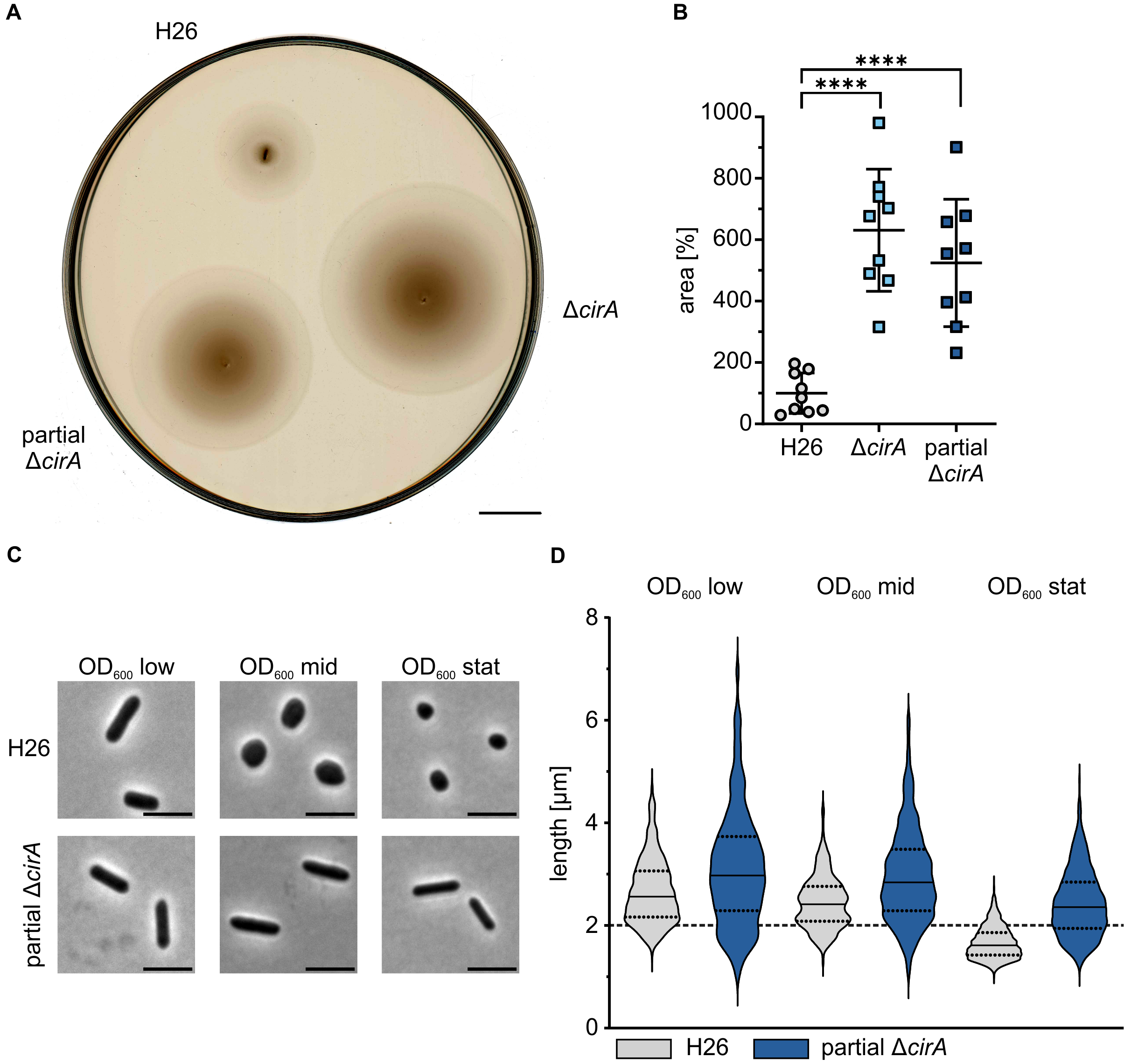

Supplement: S25 Fig — (A) Motility assay comparing wild type H26 + pTA1392 with the cirA + pTA1392 deletion strain and the partial cirA deletion strain. An exemplary motility plate is shown. (B) The area of the motility halos was measured and normalized to the average area of wild type halos showing significantly (p ≤ 0.0001) increased motility in the cirA and the partial cirA deletion strain deletion strain compared to the H26 control. Samples were measured in biological and technical triplicates and all single data points per strain were plotted. The middle line indicates the mean and the upper and lower line the standard deviation. (C) Cell shape analysis of the wild type H26 + pTA1392 and the partial cirA + pTA1392 deletion strain at low, mid and stationary OD600. The partial cirA deletion strain showed the same phenotype as the full ΔcirA strain. Cells stayed rod-shaped during the full growth cycle. Scale bar 4 µm. (D) Cell shape was analyzed using MicrobeJ and the summarized results of three independent biological replicates per strain and OD600 value plotted as violin-plots. The median is indicated by the middle black line, dotted lines indicate the first and third quartile. For each condition more than 1000 cells were analyzed. The dotted line indicates the length below which cells are considered plate shaped. (TIF) [file pgen.1012198.s037.tif]
