# Supplementary material for: CsmR controls both, motility and cell shape, in Haloferax volcanii
Source: PLoS Genet. 2026 Jun 12;22(6):e1012198. doi: 10.1371/journal.pgen.1012198 (PMC13286277; doi:10.1371/journal.pgen.1012198)
Supplement: Attachment — Submitted filename: CsmR.review.pdf [file pgen.1012198.s038.pdf]

Archaea are a diverse and ubiquitous domain of life found not only in extreme environments, but also in soils, oceans, and the human microbiome. Despite their broad ecological importance, the regulatory networks controlling fundamental behaviors such as motility and cell shape remain poorly understood. In *Haloferax volcanii*, motility depends on the archaellum, yet the transcriptional hierarchy governing archaellum biogenesis and function has remained unclear.

In this study, the authors identify CsmR as a regulator of archaellum-dependent motility. Deletion of *csmR* abolishes motility, whereas overexpression enhances it. Although deletion does not produce a clear morphological phenotype, overexpression increases the proportion of rod-shaped cells, suggesting that CsmR influences morphological state without demonstrating a direct mechanistic linkage between motility and cell shape. Intriguingly, transcriptomic analysis reveals that loss of CsmR results in upregulation of numerous motility and chemotaxis genes—as well as rod-determining factors—despite the non-motile phenotype, indicating a more complex regulatory architecture than simple activation or repression. The data further suggest interplay between CsmR, the KaiC-like regulator CirA, and the transcription factor RosR, pointing to layered transcriptional and post-transcriptional control of motility-associated pathways. Together, this work establishes CsmR as a regulator of archaeal motility and advances our understanding of transcriptional network complexity in haloarchaea.

Title

[Lines 1–2]

- While the manuscript convincingly demonstrates that CsmR controls motility, likely through direct transcriptional regulation, its role in cell shape is less well supported. A shape phenotype is observed only in overexpression strains, and the underlying mechanism of this is not thoroughly examined. The data do not demonstrate that motility and cell shape in *Hfx. volcanii* are mechanistically linked through CsmR; rather, they show that CsmR influences both processes to varying degrees. I recommend revising the title to better reflect this distinction, for example by replacing "linked" with "controlled" or "regulated."

Abstract

[Line 21]

- As noted above, consider replacing "links" when describing cell shape and motility/archaellum biogenesis in the context of CsmR.

[Line 26]

- The phrase "likely fine-tuning" overstates the data, as no direct evidence for CirA function is presented. "Potentially" would be more appropriate unless additional

discussion of KaiC-like protein mechanisms is incorporated. Currently, the known mechanistic roles of KaiC-like proteins are not discussed.

[Line 27]

- Similar to the title, terms such as "link" or "integrator" may overstate the findings. The data show that CsmR impacts these processes, but not that it mechanistically connects or integrates them.

Introduction

[Line 102]

- *"Conversely, Disk-Determining Factor A (DdfA) and Sph3 are critical for maintaining a disk-shaped morphology,…"* Sph3 is important for rod-formation as noted below.

[Line 109]

- The statement that CsmR plays a "critical role" in coordinating cell shape transitions appears too strong. The ΔcsmR strain shows no cell shape phenotype during normal growth and only exhibits a phenotype upon overexpression, suggesting that CsmR is not essential for normal cell shape determination. Less definitive language is recommended.

[Line 113]

- "Environmental factors" — the manuscript would benefit from clearer discussion of known environmental influences on cell shape transitions in *Hfx. volcanii*, as it is currently unclear how these intersect with shape and motility regulation.

General Comments

- The Introduction effectively contextualizes transcriptional regulation of archaellum biogenesis and summarizes known cell shape determinants in *Hfx. volcanii*. Given that motility is dependent on cell shape, a brief section outlining environmental or growth-related factors (e.g., cell density, quorum sensing) known to influence shape transitions would strengthen the conceptual framework.
- CirA is central to the study, yet background on prior work involving CirA in *H. volcanii* and on KaiC-like proteins more broadly is lacking. Including a concise overview would strengthen the foundation for the proposed mechanistic model.

Results

[Lines 131–132]

- The section title focuses on motility but does not reflect the inclusion of cell shape and lipid data. Consider expanding the title or subdividing this section.

[Line 141 / Figure 1 / Figure 1 legend]

- Based on the Methods, all strains in this figure—including H26 and ΔcsmR—harbor plasmids, not only the overexpression strains. This is not clearly indicated in the figure panels or legends and is only mentioned in the Results. Referring to these strains simply as wild-type or deletion variants may misrepresent their genetic background.
  - Importantly, plasmids containing the pHV2 origin (as used here) have been reported by the author's lab to significantly affect cell shape and motility. Thus, plasmid carriage is an important experimental variable.
  - The presence of plasmids should be clearly disclosed in the relevant figures and legends (Figure 1, Figure 5, and applicable supplemental figures). **Additionally, to rule out plasmid-mediated effects, it would strengthen the conclusions to include comparable cell shape and motility data for wild-type and ΔcsmR strains lacking plasmids.**
- Figure 1D (and similar quantifications elsewhere) lacks statistical analysis. Statistical comparisons should be included. It would also be helpful to define the length threshold used to classify cells as rods versus disks to aid interpretation.

[Line 148]

- For the TEM images shown here and elsewhere, how many biological replicates or independent samples are represented?

[Lines 164–179]

- The lipid composition analysis is well executed and consistent with existing literature. However, its placement disrupts the narrative flow. The transition from cell shape/morphology phenotypes to lipid composition is abrupt, and it is not clearly explained how lipid changes relate to the motility phenotypes or the lack of a cell-shape phenotype in ΔcsmR. Consider relocating this section for better cohesion.

[Line 202 / Figure 2C]

- CirA is identified as differentially regulated but is not highlighted in the plot, whereas CirD is. Given CirA's importance later in the manuscript, highlighting it here would improve consistency.

[Line 231]

- See discussion comments regarding use of "repressor" versus "activator" when describing CsmR at this stage.

[Line 272]

- Is the identified CsmR binding motif similar to motifs in databases such as JASPAR, particularly from other Lrp/AsnC family regulators?

[Line 301 / Figure 4C]

- HVO_1212 is labeled as ArlC in the figure, but HVO_1212 corresponds to CirA. ArlC/E should be HVO_1213.

[Line 328–330]

- The finding that CsmR appears dominant to CirA in this regulatory network is significant and may merit inclusion as a main figure rather than supplemental. Conversely, some panels in Figure 5 (e.g., 5H–5J) may be more appropriate for supplemental material. The cirA/csmR double deletion data could potentially be incorporated into a reorganized main figure.

[Line 347]

- A p-value cutoff of 0.1 is used for this analysis. Please clarify whether this is a standard threshold for this type of analysis.

[Line 352]

- "Kinases" is broad and encompasses multiple protein families. Consider specifying histidine kinases or more generally "signaling proteins."

[Line 355 / Figure 5I]

- The UpSet plot is difficult to interpret and does not clearly align with the claims in the text. Additional explanation may improve clarity, or alternatively, consider replacing it with a visualization similar to Figure 5H.

[Line 359]

- The manuscript alternates between "plate" and "disk" to describe late-growth morphology. Use consistent terminology.

General Comments on Results Section

- The Results are thorough, well described, and largely support the conclusions. However, the absence of growth curve data for the ΔcsmR strain is notable, particularly given the

number of supplemental figures and the known impact of growth phase on cell physiology.
- In the subsection "Posttranscriptional regulation of cirA by the small RNA hvo_1211s," no main figures are included for this section despite the central role of this regulatory interaction in the proposed model. Consider moving key data from this section into the main figures to better reflect its importance.

Discussion

[Line 416]

- See previous comments regarding the use of "linking" in this context. As noted earlier, the data support that CsmR influences both motility and cell shape, but do not demonstrate a direct mechanistic linkage between the two processes.

[Line 421]

- The statement, "This suggests that CsmR is not a simple activator but may act within a more complex regulatory network," appears somewhat inconsistent with the earlier description of CsmR as a potential repressor (line 231). Only later (around line 474) is it clarified that CsmR may function as a positive regulator within the broader CsmR/CirA system, and that the apparent repression observed in transcriptomic data may reflect increased CirA-mediated repression in the absence of CsmR.
- These shifts in interpretation—from repressor to positive regulator within a larger regulatory network—can be difficult to follow. I recommend consolidating discussion of CsmR's regulatory role (activator versus repressor) into a single, cohesive section (e.g., around line 474), where all relevant evidence is considered together. Framing earlier observations more cautiously and avoiding definitive mechanistic assertions based on individual datasets would improve clarity. As the authors themselves note, additional work is needed to determine whether CsmR functions primarily as an activator, repressor, or context-dependent regulator.

[Line 437]

- Speculation regarding a potential post-translational role for CirA would be more compelling if supported by additional background in the Introduction on the mechanistic roles of KaiC-like proteins. As currently written, this proposed link lacks sufficient contextual support and reads as speculative.

[Lines 455–467]

- The statement, "The discovery of key shape-determining factors in *H. volcanii*, including RdfA and DdfA, further underscores the tight coordination between morphology and motility [31]," is not entirely clear in context. Based on the information provided earlier

regarding RdfA and DdfA, it is difficult to discern how this statement connects to the surrounding discussion, which shifts toward regulation of pili and archaella rather than cell shape directly. These concepts would benefit from clearer integration or separation to improve logical flow.

- More broadly, the role of CsmR in cell shape determination remains underdeveloped in the Discussion, despite several assertions throughout the manuscript that CsmR contributes to this process. Additional discussion addressing why the overexpression strain—but not the knockout—displays a shape phenotype would strengthen the proposed model. Integrating this with observed changes in RdfA and Sph3 expression may help clarify the authors' claims regarding CsmR's involvement in cell shape regulation.

[Lines 496–499; 518–521; Figure 6]

- Both in the text and in the proposed model, the authors suggest an intersection between the CsmR/CirA regulatory network and quorum sensing (QS)-driven cell shape and motility transitions in *H. volcanii*. While this is a plausible and interesting hypothesis, the manuscript does not present direct data demonstrating how CsmR, CirA, or RosR interface with the QS system.
- Without more explicit evidence or expanded discussion, inclusion of QS within the current model (Figure 6) may overextend the conclusions. I recommend either providing additional discussion to clearly delineate this as a speculative extension of the model or revising the figure to limit it to interactions directly supported by the data presented.
