# Supplementary material for: CsmR controls both, motility and cell shape, in Haloferax volcanii
Source: PLoS Genet. 2026 Jun 12;22(6):e1012198. doi: 10.1371/journal.pgen.1012198 (PMC13286277; doi:10.1371/journal.pgen.1012198)
Supplement: Attachment — Submitted filename: Response to Reviewers.docx [file pgen.1012198.s039.docx]

**Reviewers' comments:**
 **Reviewer #1:**

Archaea are a diverse and ubiquitous domain of life found not only in extreme environments, but also in soils, oceans, and the human microbiome. Despite their broad ecological importance, the regulatory networks controlling fundamental behaviors such as motility and cell shape remain poorly understood. In Haloferax volcanii, motility depends on the archaellum, yet the transcriptional hierarchy governing archaellum biogenesis and function has remained unclear. In this study, the authors identify CsmR as a regulator of archaellum-dependent motility. Deletion of csmR abolishes motility, whereas overexpression enhances it. Although deletion does not produce a clear morphological phenotype, overexpression increases the proportion of rod-shaped cells, suggesting that CsmR influences morphological state without demonstrating a direct mechanistic linkage between motility and cell shape. Intriguingly, transcriptomic analysis reveals that loss of CsmR results in upregulation of numerous motility and chemotaxis genes—as well as rod-determining factors—despite the non-motile phenotype, indicating a more complex regulatory architecture than simple activation or repression. The data further suggest interplay between CsmR, the KaiC-like regulator CirA, and the transcription factor RosR, pointing to layered transcriptional and post-transcriptional control of motility-associated pathways. Together, this work establishes CsmR as a regulator of archaeal motility and advances our understanding of transcriptional network complexity in haloarchaea.

**Title**

[Lines 1–2]

• While the manuscript convincingly demonstrates that CsmR controls motility, likely through direct transcriptional regulation, its role in cell shape is less well supported. A shape phenotype is observed only in overexpression strains, and the underlying mechanism of this is not thoroughly examined. The data do not demonstrate that motility and cell shape in Hfx. volcanii are mechanistically linked through CsmR; rather, they show that CsmR influences both processes to varying degrees. I recommend revising the title to better reflect this distinction, for example by replacing “linked” with “controlled” or “regulated.”

We changed the title accordingly

**Abstract**

[Line 21]

• As noted above, consider replacing “links” when describing cell shape and motility/archaellum biogenesis in the context of CsmR.

We adapted the manuscript

[Line 26]

• The phrase “likely fine-tuning” overstates the data, as no direct evidence for CirA function is presented. “Potentially” would be more appropriate unless additional discussion of KaiC-like protein mechanisms is incorporated. Currently, the known mechanistic roles of KaiC-like proteins are not discussed.

We added this to the manuscript

[Line 27]

• Similar to the title, terms such as “link” or “integrator” may overstate the findings. The data show that CsmR impacts these processes, but not that it mechanistically connects or integrates them.

We adapted the manuscript

**Introduction**

[Line 102]

• “Conversely, Disk-Determining Factor A (DdfA) and Sph3 are critical for maintaining a disk-shaped morphology,…” Sph3 is important for rod-formation as noted below.

Sph3 is important for formation of plate shaped cells as its deletion forms rods only. We corrected the manuscript.

[Line 109]

• The statement that CsmR plays a “critical role” in coordinating cell shape transitions appears too strong. The ∆csmR strain shows no cell shape phenotype during normal growth and only exhibits a phenotype upon overexpression, suggesting that CsmR is not essential for normal cell shape determination. Less definitive language is recommended.

In the experiments you recommended using cells without plasmids, we observed that the Δ*csmR* cells without plasmids were more elongated than the H26 cells. Therefore, we repeated the analysis of the cell morphology of H26 and Δ*csmR* cells containing pTA1392 and obtained the same result as with the cells without plasmids. We apologize for the error and have corrected the text accordingly. Since there is a cell shape effect now in the deletion strains at early growth stages we decided to keep the wording.

[Line 113]

• “Environmental factors” — the manuscript would benefit from clearer discussion of known environmental influences on cell shape transitions in Hfx. volcanii, as it is currently unclear how these intersect with shape and motility regulation.

We thank the reviewer for this suggestion. We have added a dedicated paragraph to Section 5.2 consolidating the known environmental influences on cell shape and motility in *H. volcanii* — including temperature, salt concentration, nutrient availability, and quorum sensing — and discussing how these may intersect with the CsmR regulatory network. We acknowledge that the molecular connections between these environmental inputs and CsmR activity remain to be established and have framed this accordingly as an open question consistent with our broader discussion.

**General Comments**

• The Introduction effectively contextualizes transcriptional regulation of archaellum biogenesis and summarizes known cell shape determinants in Hfx. volcanii. Given that motility is dependent on cell shape, a brief section outlining environmental or growth- related factors (e.g., cell density, quorum sensing) known to influence shape transitions would strengthen the conceptual framework.

We adapted the manuscript

• CirA is central to the study, yet background on prior work involving CirA in H. Volcanii and on KaiC-like proteins more broadly is lacking. Including a concise overview would strengthen the foundation for the proposed mechanistic model.

We agree, and we expanded the introduction to include information on KaiC like proteins.

**Results**

[Lines 131–132]

• The section title focuses on motility but does not reflect the inclusion of cell shape and lipid data. Consider expanding the title or subdividing this section.

We adapted the manuscript and subdivided this section

[Line 141 / Figure 1 / Figure 1 legend]

• Based on the Methods, all strains in this figure—including H26 and ∆csmR—harbor plasmids, not only the overexpression strains. This is not clearly indicated in the figure panels or legends and is only mentioned in the Results. Referring to these strains simply as wild-type or deletion variants may misrepresent their genetic background.

o Importantly, plasmids containing the pHV2 origin (as used here) have been reported by the author’s lab to significantly affect cell shape and motility. Thus, plasmid carriage is an important experimental variable.

o The presence of plasmids should be clearly disclosed in the relevant figures and

legends (Figure 1, Figure 5, and applicable supplemental figures). Additionally, to rule out plasmid-mediated effects, it would strengthen the conclusions to include comparable cell shape and motility data for wild-type and ∆csmR strains lacking plasmids.

We use the empty plasmid to have cells grown more reliably and to have a more homogenous cell shape. However, as you suggested we performed cell shape and motility experiments with cells that do not contain a plasmid with the same result as for cells with plasmid. Results are summarized in Supplementary Figure 3.

We did not note the absence of plasmids in the figures because we believe it would distract the reader and rendering the figure more confusing; however, we have updated all figure captions to draw attention to the plasmids.

• Figure 1D (and similar quantifications elsewhere) lacks statistical analysis. Statistical comparisons should be included. It would also be helpful to define the length threshold used to classify cells as rods versus disks to aid interpretation.

We performed statistical analysis but do not want to include them into the figures in order to keep the figures clear and concise for the reader. Regarding the absence of significance annotations in the cell shape graphs, we would like to clarify our reasoning as detailed in the Materials and Methods section.

Given the large sample sizes in this study, Welch's t-test yields extremely high statistical power, such that virtually any difference between populations reaches statistical significance regardless of its biological magnitude. Under these conditions, displaying significance asterisks on plots would be misleading, as they would imply biological relevance that is not supported by the effect size data. We therefore deliberately omitted significance annotations from the cell shape plots and instead report full statistical results — including p-values and Cohen's d effect sizes — for all comparisons in Supplementary Table 4. This approach follows recommendations in the statistical literature for large-sample biological datasets (Sullivan & Feinn, 2012; Lakens, 2013).

Additionally, we added a dotted line to all cell shape plots that indicates the point below which cells are considered plate shaped only.

[Line 148]

• For the TEM images shown here and elsewhere, how many biological replicates or independent samples are represented?

We imaged three biological replicates per strain and added this information to the Material and Methods section

[Lines 164–179]

• The lipid composition analysis is well executed and consistent with existing literature. However, its placement disrupts the narrative flow. The transition from cell shape/morphology phenotypes to lipid composition is abrupt, and it is not clearly explained how lipid changes relate to the motility phenotypes or the lack of a cell-shape phenotype in ∆csmR. Consider relocating this section for better cohesion.

We included a separated section for the lipid composition analysis as you suggested

[Line 202 / Figure 2C]

• CirA is identified as differentially regulated but is not highlighted in the plot, whereas CirD is. Given CirA’s importance later in the manuscript, highlighting it here would improve consistency.

Added CirA highlight to Figure 2C

[Line 231]

• See discussion comments regarding use of “repressor” versus “activator” when describing CsmR at this stage.

We thank the reviewer for this observation. As described in our response to the related Discussion comment, we have revised the concluding sentence of Section 4.4 to avoid the premature assertion that CsmR acts as a direct transcriptional repressor based on RNA-seq data alone. The paradoxical upregulation of archaellum genes in ΔcsmR is now explicitly framed as reflecting indirect derepression through dysregulation of the CirA repressor axis, with the reader directed to Section 5.3 for the full mechanistic model. The term "repressor" is no longer used to describe CsmR at this stage of the manuscript

[Line 272]

• Is the identified CsmR binding motif similar to motifs in databases such as JASPAR, particularly from other Lrp/AsnC family regulators?

In the revised manuscript, the CsmR motif was derived directly from ChIP-seq peaks rather than inferred from promoter upstream regions. We compared the core motif to databases (including JASPAR) using Tomtom but did not detect significant similarity to known Lrp/AsnC family motifs or other transcription factors. Thus, although CsmR belongs to the Lrp/AsnC family, its binding motif appears to be distinct from previously described members. A corresponding sentence has been added to the manuscript.

“Comparison of the core CsmR motif to known transcription factor binding sites (Tomtom, MEME Suite; including JASPAR) did not reveal significant similarity to previously described Lrp/AsnC motifs.”

[Line 301 / Figure 4C]

• HVO_1212 is labeled as ArlC in the figure, but HVO_1212 corresponds to CirA. ArlC/E should be

The labeling was indeed incorrect (HVO_1212 corresponds to CirA, not ArlC). In the revised manuscript, this figure has been removed and replaced by the ChIP-seq–based analysis.

[Line 328–330]

• The finding that CsmR appears dominant to CirA in this regulatory network is significant and may merit inclusion as a main figure rather than supplemental. Conversely, some panels in Figure 5 (e.g., 5H–5J) may be more appropriate for supplemental material. The cirA/csmR double deletion data could potentially be incorporated into a reorganized main figure.

We added the cirA/csmR data to a main figure as you suggested

[Line 347]

• A p-value cutoff of 0.1 is used for this analysis. Please clarify whether this is a standard threshold for this type of analysis.

We used a more permissive adjusted p-value cutoff (padj < 0.1) in this analysis to capture genes showing consistent trends across growth phases. Given the dynamic nature of the dataset, this threshold allowed inclusion of biologically relevant trends that would be missed with more stringent cutoffs.

[Line 352]

• “Kinases” is broad and encompasses multiple protein families. Consider specifying histidine kinases or more generally “signaling proteins.”

We changed the manuscript accordingly.

[Line 355 / Figure 5I]

• The UpSet plot is difficult to interpret and does not clearly align with the claims in the text. Additional explanation may improve clarity, or alternatively, consider replacing it with a visualization similar to Figure 5H.

We agree that UpSet plots can be less intuitive at first glance. However, in this case it provides the most appropriate way to visualize the overlap of differentially expressed genes across multiple conditions (six in total), which cannot be effectively represented in pairwise scatterplots as in Fig. 5H. The key result-a core set of shared genes consistently upregulated across all conditions- is directly supported by this analysis.

To improve clarity, we have revised the figure legend.

New figure legend:
UpSet plot showing the overlap of differentially regulated genes (padj < 0.1) across strains (∆csmR, ∆cirA) and growth phases (early, mid, stationary). Each row corresponds to a specific condition, and connected dots indicate gene sets shared between conditions. Bars represent the number of genes in each intersection. Colors indicate direction of regulation (up, down, or not significant). The analysis highlights a core set of genes consistently upregulated across all conditions.

[Line 359]

• The manuscript alternates between “plate” and “disk” to describe late-growth morphology. Use consistent terminology.

Unified to plate shaped.

**General Comments on Results Section**

• The Results are thorough, well described, and largely support the conclusions. However, the absence of growth curve data for the ∆csmR strain is notable, particularly given the number of supplemental figures and the known impact of growth phase on cell physiology.

We added a growth curve of the wildtype H26 and the csmR deletion strain. The growth curve can be found in Supplementary Figure 3.

• In the subsection “Posttranscriptional regulation of cirA by the small RNA hvo_1211s,” no main figures are included for this section despite the central role of this regulatory interaction in the proposed model. Consider moving key data from this section into the main figures to better reflect its importance.

We moved the data about the regulatory RNA to Figure 6.

**Discussion**

[Line 416]

• See previous comments regarding the use of “linking” in this context. As noted earlier, the data support that CsmR influences both motility and cell shape, but do not demonstrate a direct mechanistic linkage between the two processes.

We adapted the manuscript accordingly.

[Line 421]

• The statement, “This suggests that CsmR is not a simple activator but may act within a more complex regulatory network,” appears somewhat inconsistent with the earlier description of CsmR as a potential repressor (line 231). Only later (around line 474) is it clarified that CsmR may function as a positive regulator within the broader CsmR/CirA system, and that the apparent repression observed in transcriptomic data may reflect increased CirA-mediated repression in the absence of CsmR.

We thank the reviewer for this careful reading and agree that the manuscript contained a narrative inconsistency that made the functional characterization of CsmR difficult to follow. We have addressed this inconsistency by revising the manuscript.

• These shifts in interpretation—from repressor to positive regulator within a larger regulatory network—can be difficult to follow. I recommend consolidating discussion of CsmR’s regulatory role (activator versus repressor) into a single, cohesive section (e.g., around line 474), where all relevant evidence is considered together. Framing earlier observations more cautiously and avoiding definitive mechanistic assertions based on individual datasets would improve clarity. As the authors themselves note, additional work is needed to determine whether CsmR functions primarily as an activator, repressor, or context-dependent regulator.

We agree that the shifting characterization of CsmR's regulatory role across different sections of the manuscript made the overall narrative difficult to follow, and we have revised the manuscript to address this systematically.

[Line 437]

• Speculation regarding a potential post-translational role for CirA would be more compelling if supported by additional background in the Introduction on the mechanistic roles of KaiC-like proteins. As currently written, this proposed link lacks sufficient contextual support and reads as speculative.

We agree and added more information to the introduction and the discussion part.

[Lines 455–467]

• The statement, “The discovery of key shape-determining factors in H. volcanii, including RdfA and DdfA, further underscores the tight coordination between morphology and motility [31],” is not entirely clear in context. Based on the information provided earlier regarding RdfA and DdfA, it is difficult to discern how this statement connects to the surrounding discussion, which shifts toward regulation of pili and archaella rather than cell shape directly. These concepts would benefit from clearer integration or separation to improve logical flow.

We thank the reviewer for this observation. We agree that the sentence was poorly integrated into the respective discussion section. We have revised the text to explicitly connect the shape-determining factors RdfA and Sph3 to our own transcriptomic data, which shows upregulation of rdfA and sph3 in ΔcsmR, thereby clarifying the relevance of these factors to the surrounding discussion of CsmR-mediated co-regulation of morphology and motility. DdfA has been removed from this context as it is not supported by our transcriptomic data.

• More broadly, the role of CsmR in cell shape determination remains underdeveloped in the Discussion, despite several assertions throughout the manuscript that CsmR contributes to this process. Additional discussion addressing why the overexpression strain—but not the knockout—displays a shape phenotype would strengthen the proposed model. Integrating this with observed changes in RdfA and Sph3 expression may help clarify the authors’ claims regarding CsmR’s involvement in cell shape regulation.

We thank the reviewer for this observation and agree that this aspect of the Discussion was underdeveloped. We have added a dedicated paragraph to Section 5.3 addressing the asymmetry between the Δ*csmR* and overexpression phenotypes. We noticed in our new cell shape data of the Δ*csmR* strain that cells are consistently more elongated than wildtype at early and mid-growth phases, demonstrating that CsmR contributes to cell shape determination even in the knockout, albeit more subtly than the complete shape lock observed under overexpression. The upregulation of rdfA and sph3 in our transcriptomic data is integrated into this discussion to provide a molecular explanation for both phenotypes within a single coherent dosage-dependent model.

[Lines 496–499; 518–521; Figure 6]

• Both in the text and in the proposed model, the authors suggest an intersection between the CsmR/CirA regulatory network and quorum sensing (QS)-driven cell shape and motility transitions in H. volcanii. While this is a plausible and interesting hypothesis, the manuscript does not present direct data demonstrating how CsmR, CirA, or RosR interface with the QS system.

We thank the reviewer for highlighting this important point. We agree that the proposed intersection between the CsmR/CirA network and the quorum sensing system is not directly supported by experimental data in this study. We have revised Section 5.3 to explicitly acknowledge that while the phenotypic overlap between CsmR overexpression, ΔcirA, and disruption of the quorum sensing pathway suggests a functional intersection, no direct molecular evidence for this connection has been demonstrated. This open question has been added to Section 5.4 as a priority for future investigation.

• Without more explicit evidence or expanded discussion, inclusion of QS within the current model (Figure 6) may overextend the conclusions. I recommend either providing additional discussion to clearly delineate this as a speculative extension of the model or revising the figure to limit it to interactions directly supported by the data presented.

This concern has been addressed in our response to the previous comment. We have revised section 5.3 to explicitly frame the proposed intersection between the CsmR/CirA network and the quorum sensing system as speculative, based on phenotypic overlap rather than direct experimental evidence. Section 5.4 presents this as an open question for future investigation. The model figure legend has been revised accordingly, and any direct visual connections between the regulatory network and the quorum sensing signal in Fig 7 have been replaced with dashed lines to indicate their speculative nature.

**Reviewer #2:**

The manuscript titled “Control of motility and cell shape of Haloferax volcanii is linked by a transcriptional regulator” investigates the transcriptional coordination of motility and cell-shape transitions in Haloferax volcanii by identifying and characterizing a novel transcription factor, CsmR. The authors employ a combination of genetic manipulations, motility assays, microscopy, transcriptional profiling, and computational structural predictions to demonstrate CsmR is required for motility, and functions as a major transcriptional regulator linking motility and cell shape in response to environmental cues across distinct growth phases.

The authors show that deletion of csmR abolishes motility and archaellum formation while over expression results in hyper motility and maintenance of rod-shaped cells. RNA-Seq and promoter motif analysis indicate a prominent role for CsmR in transcriptional regulation of archaellum formation, chemotaxis and cell-shape- associated genes, and structural predictions are consistent with CsmR functioning as a DNA-binding protein. Moreover, proposing a hierarchical regulation of motility and cell shape- with CsmR as the central regulator and CirA as a fine-tuner -is significant, and identification of HVO_1211s as regulatory RNA is exciting.

The experimental approaches are appropriate and data analyses are done well. The manuscript is written clearly mostly.

Taken together, this study addresses an important question regarding how environmental cues are transmitted to cellular machinery to elicit coordinated changes in motility, morphology, and physiology. However, the current data provide indirect and partial support for hierarchical regulation of motility, cell shape and environmental adaptation by CsmR, CirA and RosR. Below are my comments from major to minor, addressing these points would substantially strengthen the study and its contribution to the field.


1. The central conclusion of the manuscript is that CsmR functions as a transcriptional regulator that directly controls expression of motility- and cell-shape–associated genes. While RNA-seq, promoter motif analysis, and computational structural predictions support this a direct experimental evidence that CsmR binds to DNA or target promoters is missing. Demonstrating CsmR binds DNA using available methods, such as ChIP-qPCR, ChIP-seq, electron mobility shift assay (EMSA), or promoter-reporter assays with mutated motif would significantly strengthen the conclusion that CsmR directly regulates the listed target genes.

We agree that direct evidence of DNA binding is essential. In the revised manuscript, we addressed this by performing ChIP-seq, which provides in vivo evidence for CsmR binding across the genome. This analysis identified high-confidence binding sites and enabled the definition of a reproducible consensus motif. Importantly, many of these binding sites are located in promoter regions of differentially expressed genes, supporting direct regulation by CsmR.

2. Deletion of csmR leads to upregulation of motility and chemosensory genes while complete loss of motility is observed across growth phases. Despite promoter motif enrichment and structural predictions suggest that CsmR may directly regulate these genes, the manuscript does not clearly distinguish direct transcriptional effects from indirect or compensatory responses might be arising from disrupted motility or signaling. Clarify to distinguish CsmR primarily acts as a direct regulator or whether the observed transcriptional changes is a compensatory response would be significant and important for the mechanistic understanding.

We thank the reviewer for this important point. We agree that distinguishing direct transcriptional regulation by CsmR from indirect or compensatory transcriptional responses is critical for mechanistic interpretation, and we appreciate the opportunity to clarify this.

Our ChIP-seq analysis directly addresses this concern. By integrating ChIP-seq binding data with RNA-seq differential expression data, we were able to classify CsmR target genes into distinct categories based on the presence of the consensus binding motif (TATCA(N₄)TGATA), strand orientation, and promoter-proximal distance. Genes associated with motif-containing, strand-consistent, promoter-proximal CsmR peaks showed the strongest and most consistent upregulation in ΔcsmR across all growth phases, establishing them as high-confidence direct transcriptional targets. This includes the entire archaellum operon and chemotaxis-associated genes. Genes without detectable CsmR binding showed little or no transcriptional change, arguing against a broad compensatory response as the primary driver of the observed upregulation.

Furthermore, CsmR transcript levels remained stable across all growth phases and conditions, indicating that its activity is not primarily regulated at the transcriptional level, which is consistent with a direct and constitutive role in promoter occupancy rather than an indirect regulatory cascade. Taken together, the ChIP-seq data provide strong evidence that the transcriptional upregulation of archaellum and chemotaxis genes in ΔcsmR reflects direct derepression of CsmR-bound promoters rather than a compensatory response to disrupted motility or signaling. We have clarified this distinction more explicitly in Section 5.3 of the revised Discussion.

3. The manuscript introduces CsmR as a novel transcriptional regulator of motility and cell shape in Haloferax volcanii, but its conservation among closely related haloarchaeal species is not explored. A basic homology analysis (e.g., BLAST-based identification of CsmR homologs), together with examination of synteny (e.g., proximity to archaellum or chemotaxis genes), would be helpful in determining if CsmR represents a conserved regulatory feature within haloarchaea or a species-specific adaptation. This analysis would provide valuable evolutionary context and contribute to the impact of the study.

We added a Taxonomic profile of arCOG03067 from the EggNOG database (6.0.5) and provide a Gene Cooccurrence plot of csmR together with the archaellin genes based on the STRING database showing that they all cooccur in the Stenosarchaea group. Information can be found in Supplementary Figure 1.

4. CsmR has been implied many times as an integrator of environmental or metabolic signals, largely based on its classification within the Lrp/AsnC family and comparisons to bacterial regulators and prior work on TrmB/TbsP. However, no evidence for metabolic regulation of CsmR is presented, I suggest rephrasing such sentences (for example, lines 111-113) to clearly distinguish possible roles from demonstrated functions.

We thank the reviewer for this important clarification. We agree that describing CsmR as an integrator of metabolic signals goes beyond what our data directly demonstrate, and we have revised the manuscript accordingly. We have rephrased lines 111–113 to clearly distinguish demonstrated functions from speculative roles, removing the implication of direct metabolic sensing.

However, we would like to retain the term 'environmental' in this context, as we argue it is justified by our findings. Archaellum-driven motility in *H. volcanii* is itself an adaptive response to environmental conditions: motile rod-shaped cells are capable of moving away from unfavorable conditions, while the transition to non-motile disk-shaped cells is triggered by environmental cues such as increasing population density and quorum sensing signals (Schiller et al., 2024; Chatterjee 2025). Since CsmR impacts both archaellum biogenesis and the rod-to-disk shape transition, it sits at the nexus of a pathway whose physiological output — motility — is inherently environmental in nature. We therefore propose that describing CsmR as a regulator that links cell shape and motility to environmental adaptation is well-supported by the data, even in the absence of direct evidence for metabolic sensing. We have revised the relevant sentences to make this distinction explicit, framing CsmR as a regulator of environmentally relevant behaviors rather than a direct sensor of metabolic or environmental signals.

5. The manuscript looks at transcriptional changes at OD600 = 0.02, 0.2, and 2.0, corresponding to early, mid-exponential, and stationary phases, respectively. Additional clarification on the rationale for selecting OD600 = 2.0 as the stationary time point would be helpful since at this density cells may also be experiencing stress responses that could interfere with interpretation of physiological adaptation related specifically to motility and cell shape. Providing a growth curve and/or brief justification for these OD cutoffs would help.

We added a growth curve of the wildtype H26 and the csmR deletion strain. The growth curve can be found in Supplementary Figure 3.

An optical density of 2 is at the transition phase from exponential to stationary phase. We chose this OD to avoid side effects caused by stress that might appear at later points during stationary phase.

6. HVO_1211s is described as a regulatory RNA and refers to it as an sRNA, while also suggesting that it stabilizes cirA mRNA through extensive sequence overlap. It would be helpful to clarify the mechanistic basis for this proposed stabilizing effect and to more clearly define how HVO_1211s should be classified.

We have replaced 'sRNA' with 'regulatory RNA' throughout the manuscript, as this broader term more accurately reflects the current state of knowledge regarding HVO_1211s, particularly given its proposed stabilizing function through extensive sequence overlap with the *cirA* mRNA. Classical sRNAs typically act through short imperfect base-pairing to repress target mRNAs, whereas the extensive complementarity between HVO_1211s and cirA is more reminiscent of a cis-encoded antisense RNA mechanism, in which near-perfect overlap can protect the target transcript from degradation rather than promoting it. We acknowledge that the precise mechanistic basis remains to be experimentally validated and have framed this accordingly as a proposed mechanism requiring further investigation

7. Different adjusted P-value cutoffs (0.05 to 0.1) are used in RNASeq analyses but the rationale for using these different thresholds is not always stated. Justification for the choice of adjusted P-value cutoffs, especially where padj < 0.1 is used to define gene sets for downstream analyses, would be important. In addition, at line ~263 (arCOG enrichment analysis), results are described using “P-value < 0.05” without specifying whether this is raw P-value or adjusted value.

We agree that the rationale for the different adjusted P-value thresholds was not sufficiently explained. In the revised manuscript, we now distinguish clearly between thresholds used for primary differential expression analyses and those used for exploratory downstream analyses. Differentially expressed genes were generally defined using padj < 0.05. For the shared ΔcsmR/ΔcirA overlap analysis, we used padj < 0.1 to define a broader candidate set for exploratory downstream interpretation, and this rationale is now stated explicitly.

We also clarified the arCOG enrichment analysis. In this case, the enrichment analysis was used to summarize broad functional trends among RNA-seq-defined gene sets, and the reported values refer to raw overrepresentation P-values from goseq rather than multiple-testing adjusted category-level values. We now state this explicitly in the Methods, Results, and figure legends and describe these enrichment results as exploratory.

8. OD measurements of cells for northern blot analysis (Section 6.11, line ~740), are at 650nm however OD600 is used elsewhere in manuscript to define growth phases and sampling points. What is the rationale for using OD650? It would be more consistent to use a single wavelength in the manuscript, or explicitly state how OD650 values relate to OD600. This would be important for clarity and reproducibility.

The difference in wavelength comes from different measurement standards in the labs. OD 650 measurements were only used when the cells were used to prepare RNA to determine the size of the sRNA via Northern-Blotting. The anti-cirA Northern-blots were made with RNA extracted from cells that were harvested at OD 0.02 and 0.2 measured at 600 nm like for the rest of this study.

**Reviewer #3:**

This interesting manuscript reports on the identification of a regulator of motility and cell shape in archaea. Until now such a main regulator was not yet identified in haloarchaea. This regulator has connections with different other regulators involved in cell shape and motility that were discovered in recent years. Therefore, this is a very timely manuscript and also important for the community.
The introduction well written, required information given and it is coming quickly to the point. The quality of the figures in this manuscript is very high. The paper contains a very high density of good quality data. The paper has a high number of (supplementary) figures. The regulatory network seems quite complex and the data points towards a multi-layered regulatory network. This makes the manuscript at times difficult to follow.
I have several suggestions to make the manuscript easier to read for a broader audience.

**Introduction/abstract**
Line 20: to make it more accessible for a broader audience: add short explanation of EarA. For non-archaeal readers this might not be evident.
We added a short explanation

Line 27: archaellum gene expression ?

We adapted the manuscript

Line 28: Generalizing CsmR's role to all Haloarchaea lacks supporting evidence, as no comparative analysis is presented for ortholog presence, synteny near archaellin genes, or conservation in other Haloferax species or haloarchaea (e.g., Hfx. mediterranei, Hbt. salinarum, where multiple Lrp/AsnC regulators exist but vary in function). In order to justify the broad claims, add a bioinformatics survey (e.g. BLAST and synteny plots) of CsmR-like genes across haloarchaea. Otherwise, limit the survey to H. volcanii.
We provide a Gene Cooccurrence plot in Supplementary Figure 1 of *csmR* together with the archaellin genes based on the STRING database showing that they all cooccur in the Stenosarchaea group

**Results**
Line 137: It is difficult to assess whether the cells lack archaella, as the entire cell cannot be seen in the micrographs.

We focused on the cell pole area were archaella are assembled in Haloferax volcanii. We provided uncropped images in Supplementary Figure 4.

Furthermore, line 160 states that the aim was to test whether the csmR deletion leads to cell shape defects. Were the shapes not visible in the electron micrographs?

Phase contrast microscopy enables the extraction of cell shape parameters of thousands of cells making the claims more robust than observing individual cells with the TEM.

Line 165: the authors write: ‘To see if those morphological changes impact the composition of membrane lipid types the abundance of several lipid species present in….. ‘At the end of this paragraph they conclude ‘Hence, it is unlikely that the observed changes in cell morphology during growth are caused by an altered lipid composition.’. This analysis of the cell lipid composition comes a bit abrupt and it is not completely clear what the purpose of this analysis is and what the causative order is. Please make this clearer in the text.

Since Haloferax undergoes the transition from rod to plate shaped cells which necessitates remodeling of the cell membrane we were curious if this process also has an impact on the lipid composition.
In the revised version of the manuscript, we made added a dedicated section for the lipid composition analysis to separate it from the csmR phenotypic analysis so it can stand for itself.

Line 169: PG maybe write out in full first time
added

Fig1 : complementation with CsmR in the deletion of the CsmR strain leads to hypermotility, likely due to the strong xylose promoter. Is it possible to use less xylose and see then only partial complementation or is this promoter still quite leaky?

We made motility plates with 0, 4- and 8-mM Xylose. The results are summarized in Supplementary Figure 5.

Figure 1A: The absence of scale bars from the motility halo images prevents quantitative size comparisons across conditions. Please add them with specified units.

We added 2 cm scale bars to all motility plates

Figure 1D: Graph lacks a statistical analysis to support the significance claims. Furthermore, the assertion that csmR overexpression in H26 stabilizes rod shapes throughout growth phases is undermined by length distributions that resemble mixed populations during the mid-growth phase. I recommend including statistics and clarifying this.

We performed statistical analysis but do not want to include them in the figures to keep the figures clear and concise for the reader. Regarding the absence of significance annotations in the cell shape graphs, we would like to clarify our reasoning as detailed in the Materials and Methods section.

Given the large sample sizes in this study, Welch's t-test yields extremely high statistical power, such that virtually any difference between populations reaches statistical significance regardless of its biological magnitude. Under these conditions, displaying significance asterisks on plots would be misleading, as they would imply biological relevance that is not supported by the effect size data. We therefore deliberately omitted significance annotations from the cell shape plots and instead report full statistical results — including p-values and Cohen's d effect sizes — for all comparisons in Supplementary Table 4. This approach follows recommendations in the statistical literature for large-sample biological datasets (Sullivan & Feinn, 2012; Lakens, 2013).

Fig2 C: are similar figures for mid and stationary phase available or are they looking similar and are therefore not shown?

Similar patterns were observed for mid and stationary phase and are shown more comprehensively in Supplementary Figure 19 and 20.

Fig 3D: I do not understand the different circles. The size circles indicate the nr of affected genes, but then at the same time they also indicate the significance or not. How to distinguish between these two features? Please add some information to the figure legend.

Color intensity (white to dark purple) indicates -log10(raw overrepresentation P-value), while circles denote categories with raw overrepresentation P-value < 0.05. Circle size reflects the total number of differentially regulated genes assigned to each category. These enrichment results are intended as exploratory.

Figure 5B, Figure S9 D&F, Figure S14 D&E, Figure S17 D&E: Please provide statistical analysis of cell length measurements.

See previous answer

Figure 5C, Figure S9 A&B, Figure S14A: Please provide scale bars in motility halo images.

We added scale bars to all motility plates

Line 268: two times upstream in the title. Maybe remove one

Done

Line 275: ‘half of the genes….’ How many of these 61 genes of which the upstream region was analyzed, are functionally linked with motility/chemotaxis/cell shape ect? It is mentioned that of the 21 with the motif, half of the genes are functionally linked with motility/chemotaxis/cells shape. Is this an over or underrepresentation? Please add this information, otherwise the statement doesn’t say much.

This analysis has been removed and replaced by the ChIP-seq–based analysis in the revised manuscript.

While in silico evidence (AlphaFold3 β-sandwich core, pLDDT>90; sequence homology) supports predicted Lrp/AsnC family membership for CsmR, firm claims that it encodes a functional transcriptional regulator might overstate the data. RNA-seq reveals transcriptional changes, but lacks proof of direct DNA binding/regulation. The standard method for proving these claims would be EMSA/footprinting on target promoters, including autoregulation; ideally ChIP-seq for direct binding proof. As a reader, one might wonder why not EMSAs were attempted. I assume that this is because purification and DNA interaction studies with such a halophilic protein are not possible to perform. This would be a good location to outline the experimental challenges of this model system and justify why only in silico methods were used.

We agree that direct evidence for DNA binding is important. In the revised manuscript, we addressed this by performing ChIP-seq, which provides in vivo evidence for CsmR binding across the genome and supports its role as a transcriptional regulator. This analysis identified high-confidence binding sites and a reproducible consensus motif.

Recommended revisions are also replaced definitive phrasing (e.g., "master regulator") with "putative regulator" or "modulates".

We thank the reviewer for this suggestion. However, in light of new ChIP-seq data generated since the initial submission, we are confident in retaining strong regulatory language for CsmR. Our ChIP-seq results demonstrate direct, genome-wide binding of CsmR to the promoter regions of archaellum and chemotaxis genes, providing evidence that CsmR functions as a central transcriptional regulator of archaellum biogenesis rather than a peripheral or modulatory factor.

The authors use several structural prediction tools to study the possibility of CsmR DNA binding capabilities. I would further recommend to perform a structure-based multiple sequence alignment of CsmR with characterised Lrp/AsnC family members (e.g. E. coli Lrp/AsnC and B. subtilis LrpC). This should highlight the conserved residues in the N-terminal HTH DNA-binding domain and the C-terminal oligomerisation/effector pocket, providing robust support for the predicted family membership alongside AlphaFold3.

We have performed a structure-based multiple sequence alignment of CsmR with characterized Lrp/AsnC family members. This analysis has been added to the manuscript in Supplementary Figure 10 and provides the structural context requested, complementing our AlphaFold3 predictions and further supporting the classification of CsmR as a functional Lrp/AsnC-type regulator

Fig 5I: can some more information be added to the legend? I find it difficult to understand the figure.

We have expanded the figure legend of the UpSet plot to more clearly describe how intersections between conditions are represented and how the plot should be interpreted.

**Material & Methods**
Line 661: A paired t-test is appropriate only when mutant and wild-type (WT) measurements are directly paired within the same experimental unit, such as on identical plates or from matched biological replicates. However, the figure legends (Fig. 1, line 144; Fig. 5, line 364, Fig S9, Fig. S14, Fig. S17, Fig. S18) describe motility halo areas normalised to the average WT halo area across replicates, treating the groups as independent. I recommend revising to an unpaired (independent) two-tailed t-test.
We thanking the reviewer for pointing this us. Actually, we wrongly described the statistical test used in this case and performed an unpaired Welch’s t-test for all data from motility plates. We corrected the manuscript.

Line 662: When harvesting different volumes of culture (1 L, 20 mL or 3 mL) for RNA sequencing, there is a risk of distorting gene expression profiles due to physiological artefacts such as nutrient gradients and oxygen limitation that affect transcription independently of cell density. From the M&M I cannot extract if different container types for cultivation also lead to different processing times. e.g. whether centrifugation or filtration is used, and how long pellets are left to settle.
Please provide full details on harvesting method and pellet processing times. I understand that there are practical considerations and experimental constraints. Therefore, it would be good to describe the different treatment in the M&M and also (maybe in the discussion) mention that these might also impact gene expression.

We thank the reviewer for highlighting this important methodological point. We agree that differences in harvesting volumes and vessel types could in principle introduce physiological artefacts, and we appreciate the opportunity to clarify our approach.

To ensure comparable oxygen availability and minimize volume-to-surface-area artefacts across conditions, cultures were grown in vessels scaled proportionally to the culture volume: the 1 L culture was grown in a 5 L flask, the 20 mL culture in a 100 mL flask, and the 3 mL culture in a 15 mL tube. This was done deliberately to provide equivalent aeration across all conditions and limit condition-specific physiological side effects. These details have been added to the Materials and Methods section.

**Discussion/model/ abstract:** I like the division of the discussion in a summary, general discussion and model good. However, I find it still hard to follow the model. What is now the hypothesis that the authors have that CsmR knock-out leads to a non-motile phenotype, while at the same time leading to increased expression of archaellum genes?

We thank the reviewer for this constructive feedback. We have revised Section 5.3 to open with a single signposting sentence that explicitly states the central paradox and its resolution upfront, before the detailed mechanistic discussion unfolds.


In the model it is implied that archaellation and cell shape are necessary coupled together. Is this really the case? In different haloarchaea there are reports of round cells that can also still be archaellated. Should this not be separated in the model?

We thank the reviewer for this insightful comment. The reviewer is correct that archaellation and cell shape are not obligatorily coupled across haloarchaea, and we appreciate the opportunity to clarify this distinction in our model.

Indeed, reports from other haloarchaeal species demonstrate that round or plate-shaped cells can retain archaellum filaments, indicating that cell shape and archaellation are not universally interdependent processes. In *H. volcanii* specifically, however, the two phenomena are functionally linked under standard growth conditions: plate-shaped cells, while retaining assembled archaellum motors, lack filaments and are non-motile, whereas rod-shaped cells are archaellated and motile (Li et al., 2019). Our model is therefore intended to reflect the regulatory logic specific to *H. volcanii* rather than a universal principle across haloarchaea. We tried to make that clearer at the beginning of the discussion.

The authors discuss quorum sensing. Does the way that they sample cells also result in differences in quorum sensing between the samples, for example when the samples are concentrated in the process of harvesting them?

We thank the reviewer for this thoughtful point. The concern is valid in principle: concentrating cells during harvesting could theoretically alter the local concentration of quorum sensing signals, potentially triggering transcriptional responses that do not reflect the original growth condition. However, we believe the impact on our experimental conclusions is minimal for the following reasons.

In *H. volcanii*, the characterized quorum sensing signal accumulates as a function of population density in the growth medium (Chatterjee et al., 2025). Since cells were harvested by centrifugation upon reaching the target optical density, the quorum sensing signal concentration experienced by the cells during growth reflects the actual population density at the time of harvest. The subsequent concentration of cells during pelleting occurs on a timescale of 10 – 20 min during which cells were kept at 4°C and arresting all transcriptional activity. We therefore consider it unlikely that artificial signal accumulation during harvesting significantly altered the transcriptional profiles captured in our RNA-seq data.
