# Supplementary material for: CsmR controls both, motility and cell shape, in Haloferax volcanii
Source: PLoS Genet. 2026 Jun 12;22(6):e1012198. doi: 10.1371/journal.pgen.1012198 (PMC13286277; doi:10.1371/journal.pgen.1012198)
Supplement: Attachment — Submitted filename: Response to the reviewers.docx [file pgen.1012198.s040.docx]

Response to the reviewers

Reviewer #2: One minor revision is needed in Supplementary figure1: the gene cooccurance plot could use a legend to aid the reader in interpreting the figure.

Response: Thanks for bringing this to our attention. We have changed the legend to:

The sunburst chart in (A) depicts the taxonomic distribution of *csmR*-encoding species across the domain Archaea. Concentric rings represent successively finer taxonomic levels, moving outward from the domain level (center) through phylum, class, and order. Data were retrieved from the EggNOG database [5]. The angular size of each sector is proportional to the number of species within that taxon harboring a *csmR* ortholog. Colors distinguish major archaeal lineages: pink/red shading denotes Euryarchaeota (predominantly Halobacteria), and green shading denotes the TACK superphylum (predominantly Thaumarchaeota/Nitrosopumilaceae). Percentage values on the outermost ring indicate the fraction of all *csmR*-containing species assigned to each order. The predominance of red/pink sectors reflects that *csmR* is found almost exclusively in Halobacteria, with a secondary occurrence in Thaumarchaeota (Nitrosopumilaceae, 11%).

(B) shows a gene co-occurrence plot of *csmR* with the archaellin genes *arlA1* and *arlA2* based on the STRING database [6] (Version 12.0). The phylogenetic tree on the left (based on the STRING taxonomy) shows major archaeal lineages with Bacteria and Eukaryota as outgroups. Colored squares at internal tree nodes indicate taxonomic groupings (tan/orange = Archaea; gray = Bacteria/Eukaryota). The gene locus diagram (top right) illustrates the genomic neighborhood of *csmR* in *H. volcanii*: *csmR* (purple arrow), *hvo_1208* (yellow arrow), and *arlA1* and *arlA2* (red arrows). For each taxon row, the colored squares to the right of the tree show the predicted functional co-occurrence score (as computed by STRING) for each gene relative to *csmR*: dark red indicates a high confidence score, pale pink a lower confidence score, and an empty cell indicates no predicted co-occurrence. Squares split diagonally with a black triangle indicate that the gene is present in the STRING reference genome(s) for that taxon, with the colored portion still reflecting the co-occurrence score. The two gray columns on the far right demarcate the higher-level taxonomic groupings “Archaea” and “Euryarchaeota” within the STRING taxonomy and serve as a visual reference for the broader phylogenetic context. Together, the co-occurrence pattern shows that *csmR* most strongly co-occurs with *arlA1* and *arlA2* in Halobacteria, consistent with a predicted role in regulating archaellin-based motility.
