# Supplementary material for: CsmR controls both, motility and cell shape, in Haloferax volcanii
Source: PLoS Genet. 2026 Jun 12;22(6):e1012198. doi: 10.1371/journal.pgen.1012198 (PMC13286277; doi:10.1371/journal.pgen.1012198)
Supplement: Peer review history

# Peer review: Decision Letter 0

**DOI:** 10.1371/journal.pgen.1012198.r001

**Authors:** David Hendrixson

22 Feb 2026

PGENETICS-D-26-00078

Control of motility and cell shape of Haloferax volcanii is linked by a transcriptional regulator

PLOS Genetics

Dear Dr. Albers,

Thank you for submitting your manuscript to PLOS Genetics. After careful consideration, we feel that it has merit but does not fully meet PLOS Genetics's publication criteria as it currently stands. Therefore, we invite you to submit a revised version of the manuscript that addresses the points raised during the review process.

Please submit your revised manuscript by Apr 23 2026 11:59PM. If you will need more time than this to complete your revisions, please reply to this message or contact the journal office at plosgenetics@plos.org. When you're ready to submit your revision, log on to https://www.editorialmanager.com/pgenetics/ and select the 'Submissions Needing Revision' folder to locate your manuscript file.

Please include the following items when submitting your revised manuscript:

* A letter that responds to each point raised by the editor and reviewer(s). You should upload this letter as a separate file labeled 'Response to Reviewers'. This file does not need to include responses to any formatting updates and technical items listed in the 'Journal Requirements' section below.

* A marked-up copy of your manuscript that highlights changes made to the original version. You should upload this as a separate file labeled 'Revised Manuscript with Track Changes'.

* An unmarked version of your revised paper without tracked changes. You should upload this as a separate file labeled 'Manuscript'.

If you would like to make changes to your financial disclosure, competing interests statement, or data availability statement, please make these updates within the submission form at the time of resubmission. Guidelines for resubmitting your figure files are available below the reviewer comments at the end of this letter.

We look forward to receiving your revised manuscript.

Kind regards,

David R Hendrixson, PhD

Academic Editor

PLOS Genetics

Danielle Garsin

Section Editor

PLOS Genetics

Aimée Dudley

Editor-in-Chief

PLOS Genetics

Anne Goriely

Editor-in-Chief

PLOS Genetics

**Additional Editor Comments:**

Three expert reviewers have supplied comments for your manuscript. As you can see, all reviewers expressed many positive qualities about the work, but also provided a number of comments they felt were important for improving the manuscript. Some consistent concerns involved better resolution of whether CsmR directly regulates expression of investigated motility and rod genes, along with concerns about the nature of its connection to cell morphology. There are also a number of other comments to address to improve the quality of the work. Please consider addressing many of these comments if you would like to submit a revised manuscript.

**Journal Requirements:**

If the reviewer comments include a recommendation to cite specific previously published works, please review and evaluate these publications to determine whether they are relevant and should be cited. There is no requirement to cite these works unless the editor has indicated otherwise.

1) Please ensure that the CRediT author contributions listed for every co-author are completed accurately and in full.

At this stage, the following Authors/Authors require contributions: Phillip Nußbaum, Felix Grüneberger, Felix Neuschütz, Kevin Chou, Shamphavi Sivabalasarma, Alexander Eulitz, Anna-Lena Sailer, Katharina Vogl, Marten Exterkate, Wei He, Anita Marchfelder, Dina Grohmann, and Sonja-Verena Albers. Please ensure that the full contributions of each author are acknowledged in the "Add/Edit/Remove Authors" section of our submission form.

The list of CRediT author contributions may be found here: https://journals.plos.org/plosgenetics/s/authorship#loc-author-contributions

2) We do not publish any copyright or trademark symbols that usually accompany proprietary names, eg ©,  ®, or TM  (e.g. next to drug or reagent names). Therefore please remove all instances of trademark/copyright symbols throughout the text, including:

- ® on pages: 17, 20, and 22

- TM on page: 21.

3) Please upload all main figures as separate Figure files in .tif or .eps format. For more information about how to convert and format your figure files please see our guidelines:

https://journals.plos.org/plosgenetics/s/figures

4) We have noticed that you have uploaded Supporting Information files, but you have not included a list of legends. Please add a full list of legends for your Supporting Information files after the references list.

5) Some material included in your submission may be copyrighted. According to PLOSu2019s copyright policy, authors who use figures or other material (e.g., graphics, clipart, maps) from another author or copyright holder must demonstrate or obtain permission to publish this material under the Creative Commons Attribution 4.0 International (CC BY 4.0) License used by PLOS journals. Please closely review the details of PLOSu2019s copyright requirements here: PLOS Licenses and Copyright. If you need to request permissions from a copyright holder, you may use PLOS's Copyright Content Permission form.

Please respond directly to this email and provide any known details concerning your material's license terms and permissions required for reuse, even if you have not yet obtained copyright permissions or are unsure of your material's copyright compatibility. Once you have responded and addressed all other outstanding technical requirements, you may resubmit your manuscript within Editorial Manager.

Potential Copyright Issues:

i) Figures 2A, and 5F. Please confirm whether you drew the images / clip-art within the figure panels by hand. If you did not draw the images, please provide (a) a link to the source of the images or icons and their license / terms of use; or (b) written permission from the copyright holder to publish the images or icons under our CC BY 4.0 license. Alternatively, you may replace the images with open source alternatives. See these open source resources you may use to replace images / clip-art:

- https://commons.wikimedia.org

- https://openclipart.org/.

ii) We note that Figure 6 is created through BioRender. Please confirm that you hold a Premium account and provide a pdf copy of the CC BY 4.0 Licence as provided by BioRender. For instructions on how to generate a CC BY 4.0 license for your figure, please see the guidelines here: https://help.biorender.com/hc/en-gb/articles/21282341238045-Publishing-in-open-access-resources.

If you are using the free assets from BioRender, we are unable to publish these images as they are licenced under a stricter licence than CC BY 4.0. In this case we ask you to remove the BioRender images and replace them with open source alternatives.

See these open source resources you may use to replace images / clip-art:

- https://bioart.niaid.nih.gov/

- https://bioicons.com/

- https://healthicons.org/

- https://scidraw.io/

- https://reactome.org/icon-lib

- https://www.phylopic.org/images

- https://journals.plos.org/plosbiology/article?id=10.1371/journal.pbio.3002395

6) In the online submission form, you indicated that your data will be submitted to a repository upon acceptance. We strongly recommend all authors deposit their data before acceptance, as the process can be lengthy and hold up publication timelines. Please note that, though access restrictions are acceptable now, your entire minimal dataset will need to be made freely accessible if your manuscript is accepted for publication. This policy applies to all data except where public deposition would breach compliance with the protocol approved by your research ethics board. If you are unable to adhere to our open data policy, please kindly revise your statement to explain your reasoning and we will seek the editor's input on an exemption.

7) Please amend your detailed Financial Disclosure statement. This is published with the article. It must therefore be completed in full sentences and contain the exact wording you wish to be published.

1) State what role the funders took in the study. If the funders had no role in your study, please state: "The funders had no role in study design, data collection and analysis, decision to publish, or preparation of the manuscript."

2) If any authors received a salary from any of your funders, please state which authors and which funders..

If you did not receive any funding for this study, please simply state: u201cThe authors received no specific funding for this work.u201d

**Reviewers' comments:**

Reviewer's Responses to Questions

**Comments to the Authors:**

**Please note here if the review is uploaded as an attachment.**

Reviewer #1: attachment

Reviewer #2: The manuscript titled “Control of motility and cell shape of Haloferax volcanii is linked by a transcriptional regulator” investigates the transcriptional coordination of motility and cell-shape transitions in Haloferax volcanii by identifying and characterizing a novel transcription factor, CsmR. The authors employ a combination of genetic manipulations, motility assays, microscopy, transcriptional profiling, and computational structural predictions to demonstrate CsmR is required for motility, and functions as a major transcriptional regulator linking motility and cell shape in response to environmental cues across distinct growth phases.

The authors show that deletion of csmR abolishes motility and archaellum formation while over expression results in hyper motility and maintenance of rod-shaped cells. RNA-Seq and promoter motif analysis indicate a prominent role for CsmR in transcriptional regulation of archaellum formation, cemotaxis and cell-shape- associated genes, and structural predictions are consistent with CsmR functioning as a DNA-binding protein. Moreover, proposing a hierarchical regulation of motility and cell shape- with CsmR as the central regulator and CirA as a fine-tuner -is significant, and identification of HVO_1211s as regulatory RNA is exciting.

The experimental approaches are appropriate and data analyses are done well. The manuscript is written clearly mostly.

Taken together, this study addresses an important question regarding how environmental cues are transmitted to cellular machinery to elicit coordinated changes in motility, morphology, and physiology. However, the current data provide indirect and partial support for hierarchical regulation of motility, cell shape and environmental adaptation by CsmR, CirA and RosR. Below are my comments from major to minor, addressing these points would substantially strengthen the study and its contribution to the field.

1. The central conclusion of the manuscript is that CsmR functions as a transcriptional regulator that directly controls expression of motility- and cell-shape–associated genes. While RNA-seq, promoter motif analysis, and computational structural predictions support this a direct experimental evidence that CsmR binds to DNA or target promoters is missing. Demonstrating CsmR binds DNA using available methods, such as ChIP-qPCR, ChIP-seq, electron mobility shift assay (EMSA), or promoter-reporter assays with mutated motif would significantly strengthen the conclusion that CsmR directly regulates the listed target genes.

2. Deletion of csmR leads to upregulation of motility and chemosensory genes while complete loss of motility is observed across growth phases. Despite promoter motif enrichment and structural predictions suggest that CsmR may directly regulate these genes, the manuscript does not clearly distinguish direct transcriptional effects from indirect or compensatory responses might be arising from disrupted motility or signaling. Clarify to distinguish CsmR primarily acts as a direct regulator or whether the observed transcriptional changes is a compensatory response would be significant and important for the mechanistic understanding.

3. The manuscript introduces CsmR as a novel transcriptional regulator of motility and cell shape in Haloferax volcanii, but its conservation among closely related haloarchaeal species is not explored. A basic homology analysis (e.g., BLAST-based identification of CsmR homologs), together with examination of synteny (e.g., proximity to archaellum or chemotaxis genes), would be helpful in determining if CsmR represents a conserved regulatory feature within haloarchaea or a species-specific adaptation. This analysis would provide valuable evolutionary context and contribute to the impact of the study.

4. CsmR has been implied many times as an integrator of environmental or metabolic signals, largely based on its classification within the Lrp/AsnC family and comparisons to bacterial regulators and prior work on TrmB/TbsP. However, no evidence for metabolic regulation of CsmR is presented, I suggest rephrasing such sentences (for example, lines 111-113 ) to clearly distinguish possible roles from demonstrated functions.

5. The manuscript looks at transcriptional changes at OD600 = 0.02, 0.2, and 2.0, corresponding to early, mid-exponential, and stationary phases, respectively. Additional clarification on the rationale for selecting OD600 = 2.0 as the stationary time point would be helpful since at this density cells may also be experiencing stress responses that could interfere with interpretation of physiological adaptation related specifically to motility and cell shape. Providing a growth curve and/or brief justification for these OD cutoffs would help.

6. HVO_1211s is described as a regulatory RNA and refers to it as an sRNA, while also suggesting that it stabilizes cirA mRNA through extensive sequence overlap. It would be helpful to clarify the mechanistic basis for this proposed stabilizing effect and to more clearly define how HVO_1211s should be classified.

7. Different adjusted P-value cutoffs (0.05 to 0.1) are used in RNASeq analyses but the rationale for using these different thresholds is not always stated. Justification for the choice of adjusted P-value cutoffs, especially where padj < 0.1 is used to define gene sets for downstream analyses, would be important. In addition, at line ~263 (arCOG enrichment analysis), results are described using “P-value < 0.05” without specifying whether this is raw P-value or adjusted value.

8. OD measurements of cells for northern blot analysis (Section 6.11, line ~740), are at 650nm however OD600 is used elsewhere in manuscript to define growth phases and sampling points. What is the rationale for using OD650? It would be more consistent to use a single wavelength in the manuscript, or explicitly state how OD650 values relate to OD600. This would be important for clarity and reproducibility.

Reviewer #3: This interesting manuscript reports on the identification of a regulator of motility and cell shape in archaea. Until now such a main regulator was not yet identified in haloarchaea. This regulator has connections with different other regulators involved in cell shape and motility that were discovered in recent years. Therefore, this is a very timely manuscript and also important for the community.

The introduction well written, required information given and it is coming quickly to the point. The quality of the figures in this manuscript is very high. The paper contains a very high density of good quality data. The paper has a high number of (supplementary) figures. The regulatory network seems quite complex and the data points towards a multi-layered regulatory network. This makes the manuscript at times difficult to follow.

I have several suggestions to make the manuscript easier to read for a broader audience.

Introduction/abstract

Line 20: to make it more accessible for a broader audience: add short explanation of EarA. For non-archaeal readers this might not be evident.

Line 27: archaellum gene expression ?

Line 28: Generalizing CsmR's role to all Haloarchaea lacks supporting evidence, as no comparative analysis is presented for ortholog presence, synteny near archaellin genes, or conservation in other Haloferax species or haloarchaea (e.g., Hfx. mediterranei, Hbt. salinarum, where multiple Lrp/AsnC regulators exist but vary in function). In order to justify the broad claims, add a bioinformatics survey (e.g. BLAST and synteny plots) of CsmR-like genes across haloarchaea. Otherwise, limit the survey to H. volcanii.

Results

Line 137: It is difficult to assess whether the cells lack archaella, as the entire cell cannot be seen in the micrographs. Furthermore, line 160 states that the aim was to test whether the csmR deletion leads to cell shape defects. Were the shapes not visible in the electron micrographs?

Line 165: the authors write : ‘To see if those morphological changes impact the composition of membrane lipid types the abundance of several lipid species present in….. ‘At the end of this paragraph they conclude ‘Hence, it is unlikely that the observed changes in cell morphology during growth are caused by an altered lipid composition.’. This analysis of the cell lipid composition comes a bit abrupt and it is not completely clear what the purpose of this analysis is and what the causative order is. Please make this clearer in the text.

Line 169: PG maybe write out in full first time

Fig1 : complementation with CsmR in the deletion of the CsmR strain leads to hypermotility, likely due to the strong xylose promoter. Is it possible to use less xylose and see then only partial complementation or is this promoter still quite leaky?

Figure 1A: The absence of scale bars from the motility halo images prevents quantitative size comparisons across conditions. Please add them with specified units.

Figure 1D: Graph lacks a statistical analysis to support the significance claims. Furthermore, the assertion that csmR overexpression in H26 stabilises rod shapes throughout growth phases is undermined by length distributions that resemble mixed populations during the mid-growth phase. I recommend including statistics and clarifying this.

Fig2 C: are similar figures for mid and stationary phase available or are they looking similar and are therefore not shown?

Fig 3D: I do not understand the different circles. The size circles indicate the nr of affected genes, but then at the same time they also indicate the significance or not. How to distinguish between these two features? Please add some information to the figure legend.

Figure 5B, Figure S9 D&F, Figure S14 D&E, Figure S17 D&E: Please provide statistical analysis of cell length measurements.

Figure 5C, Figure S9 A&B, Figure S14A: Please provide scale bars in motility halo images.

Line 268: two times upstream in the title. Maybe remove one

Line 275: ‘half of the genes….’ How many of these 61 genes of which the upstream region was analyzed, are functionally linked with motility/chemotaxis/cell shape ect? It is mentioned that of the 21 with the motif, half of the genes are functionally linked with motility/chemotaxis/cells shape. Is this an over or underrepresentation? Please add this information, otherwise the statement doesn’t say much.

While in silico evidence (AlphaFold3 β-sandwich core, pLDDT>90; sequence homology) supports predicted Lrp/AsnC family membership for CsmR, firm claims that it encodes a functional transcriptional regulator might overstate the data. RNA-seq reveals transcriptional changes, but lacks proof of direct DNA binding/regulation. The standard method for proving these claims would be EMSA/footprinting on target promoters, including autoregulation; ideally ChIP-seq for direct binding proof. As a reader, one might wonder why not EMSAs were attempted. I assume that this is because purification and DNA interaction studies with such a halophilic protein are not possible to perform. This would be a good location to outline the experimental challenges of this model system and justify why only in silico methods were used.

Recommended revisions is also replace definitive phrasing (e.g., "master regulator") with "putative regulator" or "modulates".

The authors use several structural prediction tools to study the possibility of Csmr DNA binding capabilities. I would further recommend to perform a structure-based multiple sequence alignment of CsmR with characterised Lrp/AsnC family members (e.g. E. coli Lrp/AsnC and B. subtilis LrpC). This should highlight the conserved residues in the N-terminal HTH DNA-binding domain and the C-terminal oligomerisation/effector pocket, providing robust support for the predicted family membership alongside AlphaFold3.

Fig 5I: can some more information be added to the legend? I find it difficult to understand the figure.

Material & Methods

Line 661: A paired t-test is appropriate only when mutant and wild-type (WT) measurements are directly paired within the same experimental unit, such as on identical plates or from matched biological replicates. However, the figure legends (Fig. 1, line 144; Fig. 5, line 364, Fig S9, Fig. S14, Fig. S17, Fig. S18) describe motility halo areas normalised to the average WT halo area across replicates, treating the groups as independent. I recommend revising to an unpaired (independent) two-tailed t-test.

Line 662: When harvesting different volumes of culture (1 L, 20 mL or 3 mL) for RNA sequencing, there is a risk of distorting gene expression profiles due to physiological artefacts such as nutrient gradients and oxygen limitation that affect transcription independently of cell density. From the M&M I cannot extract if different container types for cultivation also lead to different processing times. e.g. whether centrifugation or filtration is used, and how long pellets are left to settle.

Please provide full details on harvesting method and pellet processing times. I understand that there are practical considerations and experimental constraints. Therefore, it would be good to describe the different treatment in the M&M and also (maybe in the discussion) mention that these might also impact gene expression.

Discussion/model/ abstract: I like the division of the discussion in a summary, general discussion and model good. However, I find it still hard to follow the model. What is now the hypothesis that the authors have that CsmR knock-out leads to a non-motile phenotype, while at the same time leading to increased expression of archaellum genes?

In the model it is implied that archaellation and cell shape are necessary coupled together. Is this really the case? In different haloarchaea there are reports of round cells that can also still be archaellated. Should this not be separated in the model?

The authors discuss quorum sensing. Does the way that they sample cells also result in differences in quorum sensing between the samples, for example when the samples are concentrated in the process of harvesting them?

**********

**Have all data underlying the figures and results presented in the manuscript been provided?**

Large-scale datasets should be made available via a public repository as described in the *PLOS Genetics*   data availability policy, and numerical data that underlies graphs or summary statistics should be provided in spreadsheet form as supporting information.

Reviewer #1: Yes

Reviewer #2: Yes

Reviewer #3: None

**********

PLOS authors have the option to publish the peer review history of their article (what does this mean?). If published, this will include your full peer review and any attached files.

If you choose “no”, your identity will remain anonymous but your review may still be made public.

**Do you want your identity to be public for this peer review?**   For information about this choice, including consent withdrawal, please see our Privacy Policy.

Reviewer #1: No

Reviewer #2: No

Reviewer #3: No

[NOTE: If reviewer comments were submitted as an attachment file, they will be attached to this email and accessible via the submission site. Please log into your account, locate the manuscript record, and check for the action link "View Attachments". If this link does not appear, there are no attachment files.]

**Figure resubmission:**

While revising your submission, we strongly recommend that you use PLOS’s NAAS tool (https://ngplosjournals.pagemajik.ai/artanalysis) to test your figure files. NAAS can convert your figure files to the TIFF file type and meet basic requirements (such as print size, resolution), or provide you with a report on issues that do not meet our requirements and that NAAS cannot fix.

After uploading your figures to PLOS’s NAAS tool - https://ngplosjournals.pagemajik.ai/artanalysis, NAAS will process the files provided and display the results in the "Uploaded Files" section of the page as the processing is complete. If the uploaded figures meet our requirements (or NAAS is able to fix the files to meet our requirements), the figure will be marked as "fixed" above. If NAAS is unable to fix the files, a red "failed" label will appear above. When NAAS has confirmed that the figure files meet our requirements, please download the file via the download option, and include these NAAS processed figure files when submitting your revised manuscript

**Reproducibility:**

To enhance the reproducibility of your results, we recommend that authors of applicable studies deposit laboratory protocols in protocols.io, where a protocol can be assigned its own identifier (DOI) such that it can be cited independently in the future. Additionally, PLOS ONE offers an option to publish peer-reviewed clinical study protocols. Read more information on sharing protocols at https://plos.org/protocols?utm_medium=editorial-email&utm_source=authorletters&utm_campaign=protocols

Attachment Submitted filename: CsmR.review.pdf

# Author comment: Author response to Decision Letter 1

**DOI:** 10.1371/journal.pgen.1012198.r002

4 May 2026

Attachment Submitted filename: Response to Reviewers.docx

# Peer review: Decision Letter 1

**DOI:** 10.1371/journal.pgen.1012198.r003

**Authors:** David Hendrixson

29 May 2026

PGENETICS-D-26-00078R1

CsmR controls both, motility and cell shape, in Haloferax volcanii

PLOS Genetics

Dear Dr. Albers,

Thank you for submitting your manuscript to PLOS Genetics. After careful consideration, we feel that it has merit but does not fully meet PLOS Genetics's publication criteria as it currently stands. Therefore, we invite you to submit a revised version of the manuscript that addresses the points raised during the review process.

Please submit your revised manuscript within by Jun 28 2026 11:59PM. If you will need more time than this to complete your revisions, please reply to this message or contact the journal office at plosgenetics@plos.org. When you're ready to submit your revision, log on to https://www.editorialmanager.com/pgenetics/ and select the 'Submissions Needing Revision' folder to locate your manuscript file.

Please include the following items when submitting your revised manuscript:

* A letter that responds to each point raised by the editor and reviewer(s). You should upload this letter as a separate file labeled 'Response to Reviewers'. This file does not need to include responses to formatting updates and technical items listed in the 'Journal Requirements' section below.

* A marked-up copy of your manuscript that highlights changes made to the original version. You should upload this as a separate file labeled 'Revised Manuscript with Track Changes'.

* An unmarked version of your revised paper without tracked changes. You should upload this as a separate file labeled 'Manuscript'.

If you would like to make changes to your financial disclosure, competing interests statement, or data availability statement, please make these updates within the submission form at the time of resubmission. Guidelines for resubmitting your figure files are available below the reviewer comments at the end of this letter.

As the corresponding author, your ORCID iD is verified in the submission system and will appear in the published article. PLOS supports the use of ORCID, and we encourage all coauthors to register for an ORCID iD and use it as well. Please encourage your coauthors to verify their ORCID iD within the submission system before final acceptance, as unverified ORCID iDs will not appear in the published article. Only the individual author can complete the verification step; PLOS staff cannot verify ORCID iDs on behalf of authors.

We look forward to receiving your revised manuscript.

Kind regards,

David R Hendrixson, PhD

Academic Editor

PLOS Genetics

Danielle Garsin

Section Editor

PLOS Genetics

Aimée Dudley

Editor-in-Chief

PLOS Genetics

Anne Goriely

Editor-in-Chief

PLOS Genetics

**Additional Editor Comments:**

Two of the three original Reviewers agreed to review the revised manuscript. Both Reviewers gave very positive comments for the revised manuscript. There is one comment from Reviewer 2 that does need addressing. Therefore, the revised mansucript is being sent back to the authors for additional editing.

**Reviewers' comments:**

Reviewer's Responses to Questions

**Comments to the Authors:**

**Please note here if the review is uploaded as an attachment.**

Reviewer #2: One minor revision is needed in Supplementary figure1: the gene cooccurance plot could use a legend to aid the reader in interpreting the figure.

Other than that, the authors have adequately addressed all my prior comments, and substantially strengthened the manuscript. Particularly, the addition of ChIP-Seq data provides a significant support for direct regulation by CsmR and a critical contribution to the study. Also, the newly included phylogenetic/ conservation analyses present a valuable evolutionary context and broader relevance to the field.

Reviewer #3: I am very satisfied with how the manuscript has been revised. The chip-seq is a great addition to the paper. Moreover, all points I raised were addressed.

**********

**Have all data underlying the figures and results presented in the manuscript been provided?**

Large-scale datasets should be made available via a public repository as described in the *PLOS Genetics*   data availability policy, and numerical data that underlies graphs or summary statistics should be provided in spreadsheet form as supporting information.

Reviewer #2: Yes

Reviewer #3: Yes

**********

PLOS authors have the option to publish the peer review history of their article (what does this mean?). If published, this will include your full peer review and any attached files.

If you choose “no”, your identity will remain anonymous but your review may still be made public.

**Do you want your identity to be public for this peer review?**   For information about this choice, including consent withdrawal, please see our Privacy Policy.

Reviewer #2: No

Reviewer #3: No

[NOTE: If reviewer comments were submitted as an attachment file, they will be attached to this email and accessible via the submission site. Please log into your account, locate the manuscript record, and check for the action link "View Attachments". If this link does not appear, there are no attachment files.]

**Figure resubmission:**

-->While revising your submission, we strongly recommend that you use PLOS’s NAAS tool (https://ngplosjournals.pagemajik.ai/artanalysis) to test your figure files. NAAS can convert your figure files to the TIFF file type and meet basic requirements (such as print size, resolution), or provide you with a report on issues that do not meet our requirements and that NAAS cannot fix.

After uploading your figures to PLOS’s NAAS tool - https://ngplosjournals.pagemajik.ai/artanalysis, NAAS will process the files provided and display the results in the "Uploaded Files" section of the page as the processing is complete. If the uploaded figures meet our requirements (or NAAS is able to fix the files to meet our requirements), the figure will be marked as "fixed" above. If NAAS is unable to fix the files, a red "failed" label will appear above. When NAAS has confirmed that the figure files meet our requirements, please download the file via the download option, and include these NAAS processed figure files when submitting your revised manuscript.

**Reproducibility:**

To enhance the reproducibility of your results, we recommend that authors deposit laboratory protocols in protocols.io, where a protocol can be assigned its own identifier (DOI) such that it can be cited independently in the future. Additionally, PLOS ONE offers an option to publish peer-reviewed clinical study protocols. Read more information on sharing protocols at https://plos.org/protocols?utm_medium=editorial-email&utm_source=authorletters&utm_campaign=protocols

# Author comment: Author response to Decision Letter 2

**DOI:** 10.1371/journal.pgen.1012198.r004

1 Jun 2026

Attachment Submitted filename: Response to the reviewers.docx

# Editor report: Decision Letter 2

**DOI:** 10.1371/journal.pgen.1012198.r005

**Authors:** David Hendrixson

2 Jun 2026

Dear Dr Albers,

We are pleased to inform you that your manuscript entitled "CsmR controls both, motility and cell shape, in Haloferax volcanii" has been editorially accepted for publication in PLOS Genetics. Congratulations!

Before your submission can be formally accepted and sent to production you will need to complete our formatting changes, which you will receive in a follow up email. Please be aware that it may take several days for you to receive this email; during this time no action is required by you. Please note: the accept date on your published article will reflect the date of this provisional acceptance, but your manuscript will not be scheduled for publication until the required changes have been made.

Once your paper is formally accepted, an uncorrected proof of your manuscript will be published online ahead of the final version, unless you’ve already opted out via the online submission form. If, for any reason, you do not want an earlier version of your manuscript published online or are unsure if you have already indicated as such, please let the journal staff know immediately at plosgenetics@plos.org.

In the meantime, please log into Editorial Manager at https://www.editorialmanager.com/pgenetics/, click the "Update My Information" link at the top of the page, and update your user information to ensure an efficient production and billing process. Note that PLOS requires an ORCID iD for all corresponding authors. Therefore, please ensure that you have an ORCID iD and that it is validated in Editorial Manager. To do this, go to ‘Update my Information’ (in the upper left-hand corner of the main menu), and click on the Fetch/Validate link next to the ORCID field.  This will take you to the ORCID site and allow you to create a new iD or authenticate a pre-existing iD in Editorial Manager.

If you have a press-related query, or would like to know about making your underlying data available (as you will be aware, this is required for publication), please see the end of this email. If your institution or institutions have a press office, please notify them about your upcoming article at this point, to enable them to help maximise its impact. Inform journal staff as soon as possible if you are preparing a press release for your article and need a publication date.

Thank you again for supporting open-access publishing; we are looking forward to publishing your work in PLOS Genetics!

Yours sincerely,

David R Hendrixson, PhD

Academic Editor

PLOS Genetics

Danielle Garsin

Section Editor

PLOS Genetics

Aimée Dudley

Editor-in-Chief

PLOS Genetics

Anne Goriely

Editor-in-Chief

PLOS Genetics

www.plosgenetics.org

BlueSky: @plos.bsky.social

----------------------------------------------------

Comments from the reviewers (if applicable):

----------------------------------------------------

**Data Deposition**

If you have submitted a Research Article or Front Matter that has associated data that are not suitable for deposition in a subject-specific public repository (such as GenBank or ArrayExpress), one way to make that data available is to deposit it in the Dryad Digital Repository. As you may recall, we ask all authors to agree to make data available; this is one way to achieve that. A full list of recommended repositories can be found on our website.

The following link will take you to the Dryad record for your article, so you won't have to re‐enter its bibliographic information, and can upload your files directly:

http://datadryad.org/submit?journalID=pgenetics&manu=PGENETICS-D-26-00078R2

More information about depositing data in Dryad is available at http://www.datadryad.org/depositing. If you experience any difficulties in submitting your data, please contact help@datadryad.org for support.

Additionally, please be aware that our data availability policy requires that all numerical data underlying display items are included with the submission, and you will need to provide this before we can formally accept your manuscript, if not already present.

----------------------------------------------------

**Press Queries**

If you or your institution will be preparing press materials for this manuscript, or if you need to know your paper's publication date for media purposes, please inform the journal staff as soon as possible so that your submission can be scheduled accordingly. Your manuscript will remain under a strict press embargo until the publication date and time. This means an early version of your manuscript will not be published ahead of your final version. PLOS Genetics may also choose to issue a press release for your article. If there's anything the journal should know or you'd like more information, please get in touch via plosgenetics@plos.org.

# Editor report: Acceptance letter

**DOI:** 10.1371/journal.pgen.1012198.r006

**Authors:** David Hendrixson

PGENETICS-D-26-00078R2

CsmR controls both, motility and cell shape, in Haloferax volcanii

Dear Dr Albers,

We are pleased to inform you that your manuscript entitled "CsmR controls both, motility and cell shape, in Haloferax volcanii" has been formally accepted for publication in PLOS Genetics! Your manuscript is now with our production department and you will be notified of the publication date in due course.

The corresponding author will soon be receiving a typeset proof for review, to ensure errors have not been introduced during production. Please review the PDF proof of your manuscript carefully, as this is the last chance to correct any errors. Please note that major changes, or those which affect the scientific understanding of the work, will likely cause delays to the publication date of your manuscript.

Soon after your final files are uploaded, unless you have opted out or your manuscript is a front-matter piece, the early version of your manuscript will be published online. The date of the early version will be your article's publication date. The final article will be published to the same URL, and all versions of the paper will be accessible to readers.

For Research Articles, you will receive an invoice from PLOS for your publication fee after your manuscript has reached the completed accept phase. If you receive an email requesting payment before acceptance or for any other service, this may be a phishing scheme. Learn how to identify phishing emails and protect your accounts at https://explore.plos.org/phishing.

Thank you again for supporting PLOS Genetics and open-access publishing. We are looking forward to publishing your work!

With kind regards,

Zsofia Freund

PLOS Genetics

On behalf of:

The PLOS Genetics Team

Carlyle House, Carlyle Road, Cambridge CB4 3DN | United Kingdom

plosgenetics@plos.org | +44 (0) 1223-442823

plosgenetics.org | Twitter: @PLOSGenetics
